# New computational protein design methods for *de novo* small molecule binding sites

**DOI:** 10.1371/journal.pcbi.1008178

**Published:** 2020-10-05

**Authors:** James E. Lucas, Tanja Kortemme

**Affiliations:** 1 UC Berkeley–UCSF Graduate Program in Bioengineering, University of California San Francisco, San Francisco, CA, United States of America; 2 Department of Bioengineering and Therapeutic Sciences, University of California San Francisco, San Francisco, CA, United States of America; Penn State College of Medicine, UNITED STATES

## Abstract

Protein binding to small molecules is fundamental to many biological processes, yet it remains challenging to predictively design this functionality *de novo*. Current state-of-the-art computational design methods typically rely on existing small molecule binding sites or protein scaffolds with existing shape complementarity for a target ligand. Here we introduce new methods that utilize pools of discrete contacts between protein side chains and defined small molecule ligand substructures (ligand fragments) observed in the Protein Data Bank. We use the Rosetta Molecular Modeling Suite to recombine protein side chains in these contact pools to generate hundreds of thousands of energetically favorable binding sites for a target ligand. These composite binding sites are built into existing scaffold proteins matching the intended binding site geometry with high accuracy. In addition, we apply pools of side chain rotamers interacting with the target ligand to augment Rosetta’s conventional design machinery and improve key metrics known to be predictive of design success. We demonstrate that our method reliably builds diverse binding sites into different scaffold proteins for a variety of target molecules. Our generalizable *de novo* ligand binding site design method provides a foundation for versatile design of protein to interface previously unattainable molecules for applications in medical diagnostics and synthetic biology.

## Author summary

Despite the variety of contexts where small molecule recognition by proteins is paramount, computational methods for designing this functionality are limited in scope. Current methods are limited by the availability of structurally characterized protein-ligand complexes and the inability of design methods to incorporate appropriate residues at the protein-ligand interface that predictably yield a functional binding site. In this work we introduce a new set of methods that combines information from the Protein Data Bank (PDB) with the Rosetta Macromolecular Modeling Suite to improve our ability to design new binding sites for arbitrary small molecules. We demonstrate that highly represented protein contacts in the PDB with fragments that compose the target ligand can be identified and recombined to form hundreds of thousands of new binding sites for ligands where less than a dozen characterized complexes exist. Commonly observed protein residue–ligand contacts can also be applied in simulations to optimize the sequences of protein-ligand interfaces, resulting in improved sequence recovery and design metrics predictive of success. We hope that the methods introduced here will open protein design to new applications in small molecule sense and response systems.

This is a *PLOS Computational Biology* Methods paper.

## Introduction

Despite significant advances in *de novo* design of protein structures, innovations in algorithms and methodologies for the computational design of protein function have not kept pace [1,2]. In particular, it remains challenging to design proteins that bind new small molecule ligands [3,4]. The ability to design proteins with high-affinity interactions for defined small molecule targets is important for creating enzymes with novel substrates, receptors and transcription factors that sense and respond to unique inducer molecules, and binders that can recognize and sequester arbitrary ligands. On-demand design of proteins with defined small-molecule binding functionality would have many applications in bioremediation, synthetic biology, and medical diagnostics.

Various strategies have been developed and successfully applied to the computational design of ligand binding function [5,6]. PocketOptimizer [7] is a ligand binding pocket design framework consisting of several modules to sample, score, and solve for low-energy protein-ligand complexes. In a computational benchmark, PocketOptimizer correctly predicted mutations that impart higher affinity in small molecule ligand binding sites in 69% of cases [8]. The BBK* algorithm [9] as implemented in the OSPREY protein design software package [10] applies an ensemble-based branch-and-bound approach to efficiently identify highest-affinity sequences for ligand-binding sites in a design problem containing up to 10^6^ sequences. The Erebus web server [11] uses geometric descriptions of atoms positioned in space relative to each other to identify protein structures in the PBD that possess a potential ligand binding site of interest.

Methods for ligand design have also been applied to experimental test cases. There are several examples of *de novo* designed helical bundles that bind various ligands [12], including a recent synthetic porphyrin binder that was engineered through simultaneous optimization of the binding site and a remote, well-packed core [13]. Another common avenue for designing binders with desired function is by changing the ligand specificity of an existing binder. This can be accomplished by transplanting binding sites of a close homolog [14]. This strategy was used to impart a spermidine binding pocket from the periplasmic binding protein PotD into its homolog PotF, which natively binds putrescine. In a different example, a vanillin-responsive biosensor [15] was engineered by applying the Phoenix Match [16] algorithm to place vanillin conformers into the existing binding site of the tetR-family repressor qacR, followed by binding site design with the FASTER optimization [17] algorithm. QacR variants were experimentally validated in an *in vitro* transcription-translation system where two designs possessed sensitivity to vanillin. Design methods in the Rosetta Macromolecular Modeling Suite [18] such as the RosettaMatch application [19] have been used to reengineer proteins to bind digoxigenin [20], fentanyl [21], and 17a-hydroxylprogesterone [4] and to create a new binding site for the metabolic intermediate farnesyl pyrophosphate in a protein-protein interface to build synthetic sense/response systems [22]. The Rotamer Interaction Field algorithm was used to place a binding site for (Z)-4-(3,5- difluoro-4-hydroxybenzylidene)-1,2-dimethyl-1H-imidazol-5(4H)-one (DFHBI) into the cavity of a *de novo* designed beta-barrel [23].

Common design protocols to introduce a new ligand binding function into a protein [24] begin with the specification of the binding site, where amino acid side chains and conformations are geometrically defined to make key interactions with the target ligand. These binding site definitions are typically derived based on chemical intuition or taken from an existing protein in complex with the target ligand present in the Protein Data Bank (PDB). In a second step, the target ligand and its defined contacts are placed into a new protein (termed “scaffold”) using geometric matching, such as in the RosettaMatch application [19]. Given a set of geometric constraints defining side chain interactions with a target ligand, RosettaMatch attempts to find backbone positions in a scaffold protein where side chains can be placed to satisfy all protein-ligand contact constraints and places the ligand within the protein subject to these constraints. In a third step, sequence positions surrounding any successfully matched binding site are redesigned to accommodate the newly introduced ligand and defined side chains. The resulting design models are ranked based on predicted protein stability and interaction energy with the ligand, in addition to a range of design filters, and predictions are selected to be experimentally validated.

Despite previously demonstrated success of these protocols [20,22], there are still several limitations that prevent generalizable *de novo* design of ligand binding sites. First, binding site geometries need to be predefined for a target ligand. Even when binding site definitions can be derived from existing complexes for the target ligand in the PDB, there may only be a few examples (if any) and these might be limited to low-affinity binding interactions. Second, geometric matching algorithms are typically only capable of incorporating binding site definitions composed of 3–5 residues due to the complexity of finding backbone geometries in scaffold proteins that satisfy all user-defined constraints. To find solutions, it is necessary to relax these constraints at the expense of deviating significantly from the geometries in the binding site definition. Third, binding site definitions matched into a scaffold protein only constitute a part of the binding site and the remainder of the binding site environment needs to be optimized through algorithms that change sequence identity (design) or side chain conformations (rotamer packing) [25,26]. Common binding site design methods such as those implemented in Rosetta, including the recently developed Rotamer Interaction Field [23], rely on Rosetta’s energy function to introduce favorable interactions with the ligand. However, limitations in the energy function used for design may fail to capture important interactions with the target ligand, such as pi-cation and pi-pi interactions [27] or interactions with atom types that are commonly encountered in ligands but less well parameterized [28]. Finally, filtering steps after design typically eliminate a majority of design candidates because they possess poor shape complementarity, ligand burial, hydrogen bond satisfaction, and other metrics that are predictive of success.

Here we describe a new approach that uses protein-ligand contact observations in the PDB to address current shortcomings in defining and designing ligand binding sites. Our approach generates hundreds of thousands of new binding site definitions in an automated fashion for arbitrary target ligand conformations, regardless of whether a complete binding site definition can be derived from an existing complex in the PDB. These binding site definitions yield thousands of RosettaMatch solutions that agree well with originally defined geometries. We also introduce a new design method that recapitulates key interactions in ligand binding sites. Moreover, we show that the new design method improves several metrics in design that are predictive of success when designs are experimentally characterized. While we have incorporated these new methods in Rosetta, the principles are generally applicable to other design approaches. The methods introduced here will have broad utility to design binders for arbitrary small molecules, towards predictable design of small molecule sensors, inducible transcription factors, and other functional proteins.

## Results

**Fig 1:**
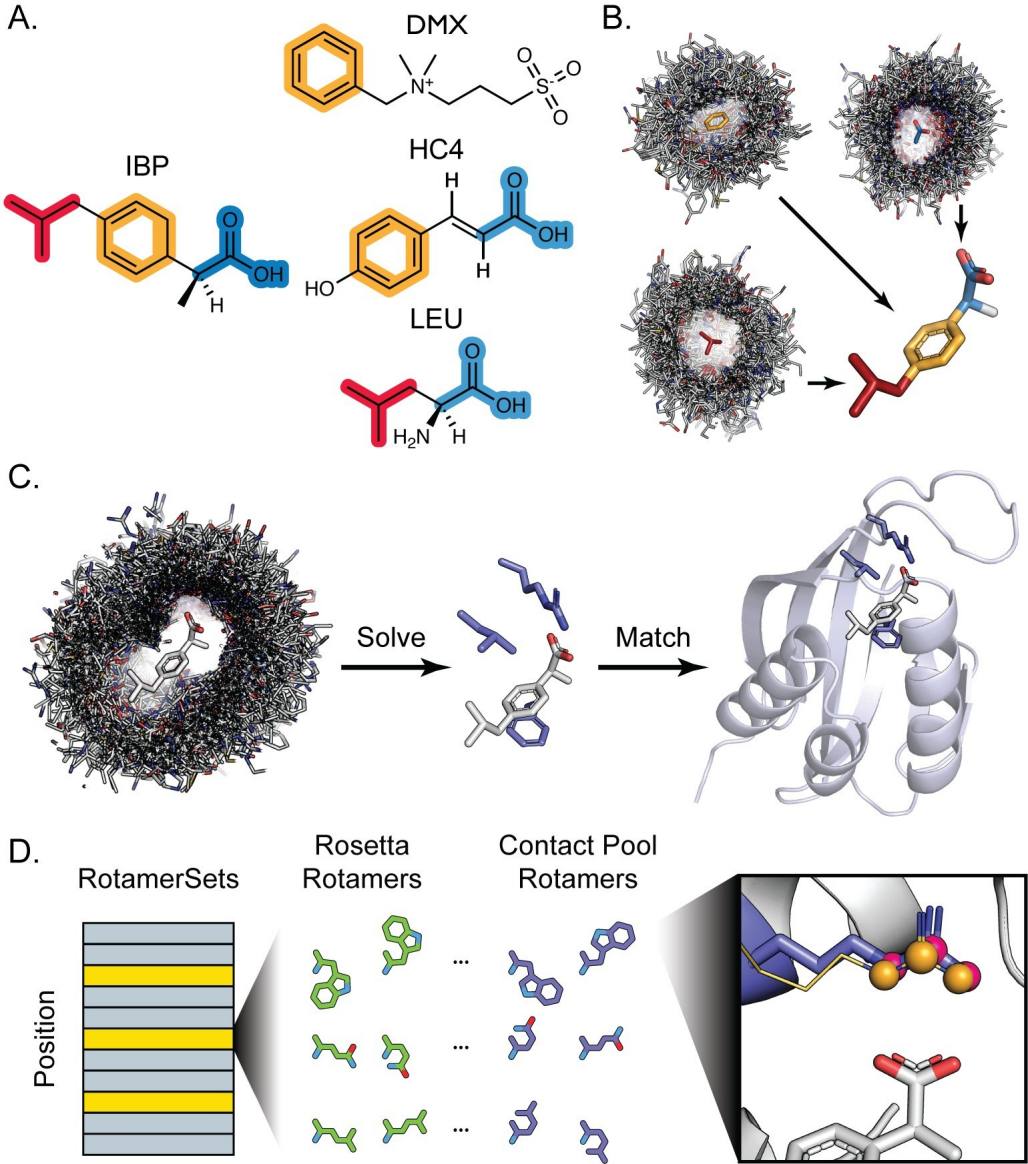
Protocol Overview. A. Target small molecules are decomposed into fragments (highlighted red, orange, and blue) that are present as a substructure in a wide range of molecules bound to proteins in the PDB. B. Protein complexes bound to substructure-containing molecules are systematically parsed to generate contact pools representing all contact modes with each fragment present in the PDB. These contacts are mapped onto the full target ligand (here ibuprofen) to create a conformer-specific contact pool for downstream steps. C. A simulated annealing protocol is used to assemble hundreds of thousands of three-residue composite binding sites from a target ligand contact pool. RosettaMatch is then used to find scaffold proteins that can geometrically accommodate a composite binding site solution. D. Sequence design is performed by Rosetta’s design machinery, where positions in the protein can be set to designable (yellow bars) or not designable (grey bars). Contact pools are used to supplement the set of rotamers generated by Rosetta for designable positions. Rotamers are built in the context of a potential ligand interface and any rotamers (purple sticks, pink spheres) that recapitulate an interaction in the contact pool (gold lines, orange spheres) are added to the standard RotamerSet (green) and provided a score bonus with the special_rot score term.

We sought to leverage the wealth of information in the PDB to address current shortcomings in existing protein-ligand interface design methods. While high-affinity protein-ligand complexes for small molecules of interest may be sparse or non-existent, our approach is built on the following: (i) Target ligands are decomposed into substructures for which there are many contacts in the PDB (Fig 1A), and (ii) these observed contacts with each ligand-derived substructure are used to assemble a pool of contacts for the target ligand (Fig 1B).

We reasoned that these contact pools should improve design of new ligand binding sites in three principal ways. First, reassembling “composite” binding sites by recombining protein-ligand substructure contacts should provide an automated method to generate a large number of potential binding site definitions (even for ligands for which no complete binding site definition exists in the PDB) that can be matched into scaffold proteins. Second, by dramatically increasing the number of binding site definitions used in the geometric matching step, we should increase the number of scaffold protein “hits” that can accommodate these geometries well. Third, contact pools should also be useful in the design step, by incorporating protein-ligand contacts that might otherwise be missed in conventional design. As a result, we reasoned that design with contact pools should increase key metrics such as protein-ligand shape complementarity in the design filtering step. To test these ideas, we first apply the contact pools of protein-ligand interactions to assemble millions of new binding site definitions that can be incorporated into protein scaffolds with RosettaMatch (Fig 1C). Second, we use contact pools to augment and inform existing design protocols (Fig 1D) [29]. In the following, we first describe the implementation of our protocol to assemble contact pools. We then show that the application of contact pools to inform design yields millions of strictly defined binding sites and improves key design metrics.

### Generation of contact pools

Our protocol produces a set of contact pools for an arbitrary number of potential ligand conformers that can be applied to either A) generate binding site definitions that can be used as constraints for the RosettaMatch application or B) generate rotamers to augment the conventional Rosetta design machinery (i.e. the Packer).

We define a “fragment” as a ligand substructure constituting a distinct chemical moiety (Fig 1A) that will form an interaction with a protein (Fig 1B). Fragments are composed of at least three atoms and are rigid substructures of the target ligand. While several automated small molecule fragmentation methods exist, these methods typically break bonds for retrosynthetic analysis in the context of fragment-based drug discovery [30,31]. The assembly of contact pools instead relies on the chemical intuition of the user to identify fragments that will mediate important interactions at the protein-ligand interface (e.g. hydrogen bond donors/acceptors, ring systems) but are not necessarily segmented by breakable bonds.

### The PDB is rich in protein-fragment contact information

**Fig 2:**
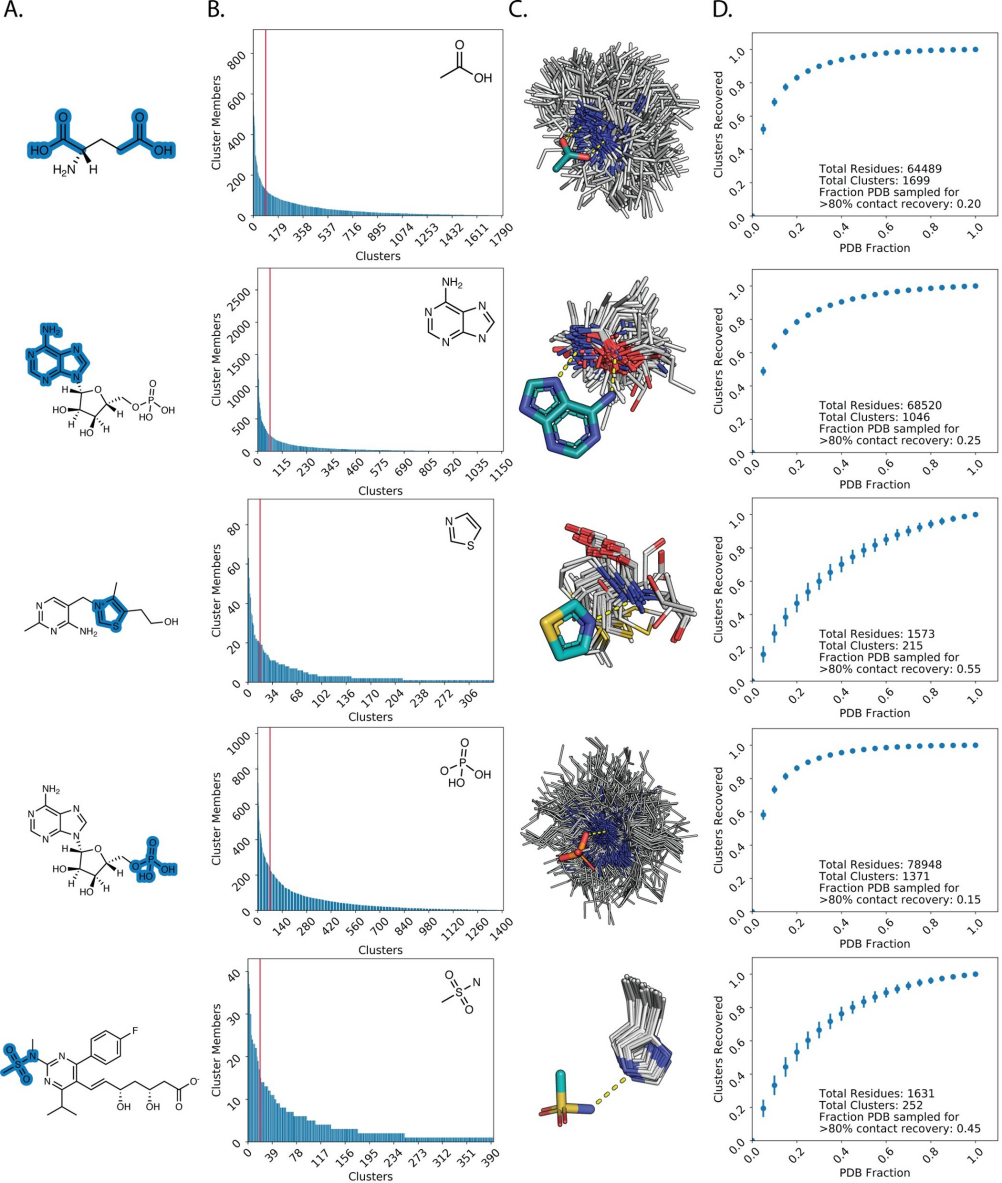
PDB sufficiently samples ligand-fragment contact space. A. Examples of fragment definitions (highlighted in blue) and context as a substructure of a small molecule. B. Clusters for each fragment are plotted in order of the number of members for each cluster (“contact mode”). Clusters to the left of the red vertical line represent the top 5% (high-occupancy) contact modes. C. Examples of high-occupancy clusters for fragments that illustrate expected favorable contact modes. White lines: clustered side chain contacts, teal: fragment carbons, orange: phosphorous, yellow: sulfur, red: oxygen, blue: nitrogen. Hydrogen bonding contacts are depicted as yellow dashed lines. D. Average and standard deviation for the number of contact modes recovered at given fractions of the PDB.

To demonstrate the number and diversity of unique types of contacts that may be observed with fragments in the PDB, we generated 34 fragments for a variety of chemical moieties that are found in common drugs, toxins, and metabolites (Fig 2A, S1 Table). All protein-ligand complexes in the PDB that contain these fragments were retrieved and transformed to superpose the ligand substructure onto a reference fragment. Only protein side chain contacts within 4Å of the ligand were kept. We defined a 16-dimensional feature vector for each protein residue encoding the type of chemical contact mediated between the residue and the fragment (see Methods). We then applied hierarchical agglomerative clustering to generate clusters of protein contacts within the contact pool that we define to mediate unique “contact modes” (i.e. clusters) with each ligand fragment.

Sorting clusters by occupancy revealed that fragments often possess a small number (<5% of all clusters) of preferred, high-occupancy contact modes with remaining clusters consisting of less prevalent contact modes (Fig 2B). This observation suggests that proteins in the PDB exhibit preferred modes of mediating interactions with the chemical environments that define each fragment. Indeed, visually inspecting clusters with the highest occupancies for various fragments revealed residue types mediating well-defined, favorable contact geometries that one would expect. For instance, the most common contact modes with the adenine fragment (second row from top) consist of bidentate hydrogen bonding interactions with the base mediated by side chain functional groups (e.g. asparagine/glutamine carboxyamide) as well as backbone carbonyl/amine functional groups (Fig 2C).

The preference for a select number of contact modes led us to investigate what proportion of the PDB would need to be sampled to recover a majority (>80%) of contact modes for each fragment. To address this question, we bootstrapped 1000 random samples for different proportions of the PDB and counted the number of unique contact modes recovered as defined by our previously generated clusters. Only a minority fraction of the PDB (typically <40%) was required to recover at least 80% of unique contact modes with each fragment (Fig 2D, S1 Fig).

### Using composite binding sites increases the number of high-quality matches

Our analysis of contact modes for ligand fragments in the PDB demonstrates the diversity of side chain identities and geometries that proteins use to mediate interactions with unique chemical moieties on the ligand. We next sought to use this diversity to expand the number of side chain compositions and geometries that could be used to define a ligand binding site for methods like RosettaMatch. Instead of using an existing binding site extracted from the PDB to define side chain interactions with a target ligand, we used protein-fragment contacts to create “composite” binding sites. We first fragmented the target ligand and generated a contact pool for each fragment using discrete side chain-fragment interactions in the PDB. We then transformed the orientation of all protein residues in each fragment contact pool relative to the source fragment in the target ligand to preserve observed contact geometries (Fig 1B). For the assembly of composite binding sites, we filtered these contacts using the fa_rep (Lennard-Jones, repulsive component), fa_atr (Lennard-Jones, attractive component), fa_elec (coulombic electrostatic potential), hbond_sc (side chain-side chain hydrogen bonds [32]), hbond_bb_sc (backbone-side chain hydrogen bonds), and fa_sol (Lazaridis-Karplus solvation [33]) two-body terms in the Rosetta REF2015 all-atom energy function [27,28]. This additional filtering step yields a collection of residue contacts with the target ligand that are not only observed in the PDB to interact with the target ligand, but are also determined to be energetically favorable by Rosetta, to serve as “hot spot” residues for composite binding sites [34]. These filtered pools contained on average 2,800 unique contacts for each ligand. This procedure can be repeated for different ligand conformers generated using a conformer search tool such as OpenEye Omega [35] or RDKit [36].

Given a contact pool for a ligand conformer, we applied a simulated annealing Monte Carlo protocol similar to the Rosetta side chain rotamer optimization method (Packer) to yield low-energy three-residue composite binding site solutions for the target ligand (Fig 1D) (here we illustrate the method with three-residue binding sites but larger sites could be used). Up to ten trajectories are attempted for each contact pool and the best 100,000 three-residue binding sites for each trajectory are recorded. The results from separate trajectories are consolidated and the 5,000 lowest-energy unique composite binding site solutions are used to generate constraints for RosettaMatch.

**Fig 3:**
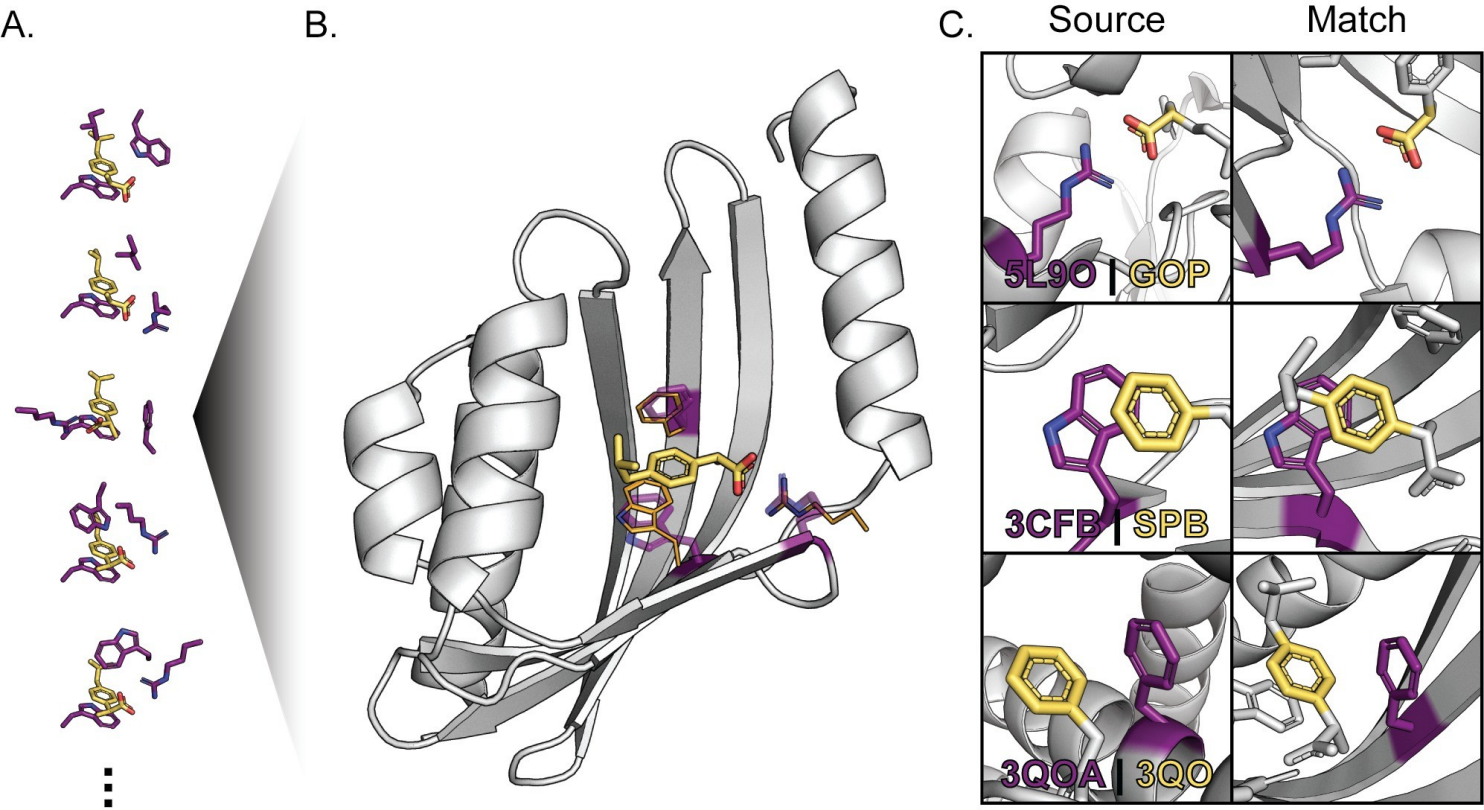
Discrete Protein-Fragment Contacts are Assembled into Composite Binding Sites. A. Composite binding sites are generated by combining discrete, observed contacts (purple side chains) with fragments that compose the target ligand (yellow) into low-energy configurations using a simulated annealing protocol. B. RosettaMatch finds scaffold proteins (grey) with existing cavities that can accommodate the target ligand with close agreement with contact residue geometries defined in composite binding site solutions (RosettaMatch residues: purple sticks, composite binding site definition: orange lines). C. Comparison of protein residue (purple) interactions with defined fragments (yellow) in the context of the protein-ligand complex that served as the source of a contact pool interaction (Source) and in the context of a match produced using RosettaMatch (Match). The source protein-ligand complex PDB ID and ligand chemical component identifier are provided for each source interaction.

Next, we used RosettaMatch to build composite binding sites into a monomer scaffold set consisting of 401 proteins that had been previously applied to successfully redesign a protein to bind a new ligand [20]. We use stringent RosettaMatch constraints (see Methods) to ensure that the match solutions found by RosettaMatch do not significantly deviate from defined constraint geometries (Fig 3). We generated 5,000 composite binding sites for eight ligands (S2 Table, S1 File) and compared the number of matches found to those found using constraints generated from existing binding sites for the corresponding ligands in the PDB (S2 File). In every case, using composite binding site solutions produces >50-fold more matches than conventional constraints derived from existing protein-ligand complexes (Table 1). Only 5000 of the lowest-scoring composite binding sites were used for this benchmark; many more potential match solutions can be found with the hundreds of thousands of composite binding site solutions generated with this method. Moreover, we find many more matches with composite binding sites that pass binding site energy, bump check, and designability quality filters (see Methods, “filtered matches” in Table 1) than when using PDB-derived constraints. Increasing the number of high-quality matches in this step is important since they serve as starting points for design, and, frequently, even good matches do not yield high-quality final design models because of difficulties generating good binding site environments in the design step. We note that our method can generate composite binding sites and large numbers of matches for ligands for which protein-ligand complexes are rare or do not exist in the PDB (e.g. chemical component identifiers ATZ, AFN in Table 1).

**Table 1:** Numbers of matches found comparing complete binding sites extracted from the PDB and composite binding sites generated by our method.

Chemical Component Identifier	Complete Binding Sites From PDB			Solved Constraints From Contact Pools		
	# Complete Binding Sites	Raw Matches	Filtered Matches	# Constraints	Raw Matches	Filtered Matches
38E	2	2141	55	5000	2430929	31430
AFN	0	0	0	5000	1560062	27806
ATZ	1	0	0	5000	3797641	22525
DOG	4	3175	94	5000	578060	5041
IBP	12	1267	83	5000	1550018	16206
IM4	2	0	0	5000	3605143	49679
LFN	2	329	53	5000	4575191	38794
NPS	7	122	20	5000	2473723	18229

Filtered match counts constitute the 25 best-scoring matches that pass quality filters for each set of matches that result from constraints for a single composite binding site and place the same residue identities at the same positions in a scaffold protein.

### Complementary rotamers improve binding site recapitulation

In addition to improving the numbers and quality of generated matches using composite binding sites, we hypothesized that contact pools could also be used to improve the design step. Since contact pools possess a wealth of information on how proteins in the PDB interact with different chemical moieties present on ligands, we sought to incorporate this information into Rosetta’s core design machinery (the Packer). We used the backbone-dependent Dunbrack library [26] to generate rotamers for protein positions at the ligand interface and added complementary rotamers that recapitulate interactions in the contact pool to the Packer RotamerSets (Fig 1C). These complementary rotamers were generated with additional χ-angle sampling for χ_1_ through χ_4_. For each rotamer built at a position within a binding site, a set of three contact atoms were defined based on contact geometry with the ligand. These atoms were compared to the same atoms for the same residue identities in the contact pool. If rotamer contact atoms achieved an RMSD of ≤1.5Å with any residue in the contact pool, it was added to the set of complementary rotamers for the current position. These additional rotamers are flagged as a “special_rot” variant [29]. An additional “special_rot” score term is enabled with a customizable bonus to bias incorporation of empirically determined residue-ligand contacts during design.

To determine an appropriate value for the special_rot score bonus, we used a native sequence recovery test [25,37–39] on a panel of protein-ligand complexes. We identified a total of 22 protein-ligand complexes from the BindingMOAD database [40] and generated contact pools for each unique ligand (S3 Table, S3 File) to create a set of empirically-determined rotamers to complement the rotamers generated by the Packer. These “complementary rotamers” were included in 5000 design trajectories for special_rot bonus values ranging from 0 (complementary rotamers are added to the Packer but no score bonus is applied) to -10 Rosetta Energy Units (REUs). For each complex, first and second shell contacts with the ligand as determined by the Rosetta Neighborhood and ClashBasedRepackShell selectors (see Methods) were subjected to design. Profile similarity (see Methods) and sequence recovery to the native complex sequence were calculated for designable positions in the benchmark complexes to determine whether complementary rotamers improve Rosetta’s ability to design the binding site environment, and if so, which special_rot score bonus value was optimal. For the purposes of the native sequence recovery benchmark, a design position is considered recovered if >50% of designs incorporate the residue identity observed in the original protein-ligand complex.

**Fig 4:**
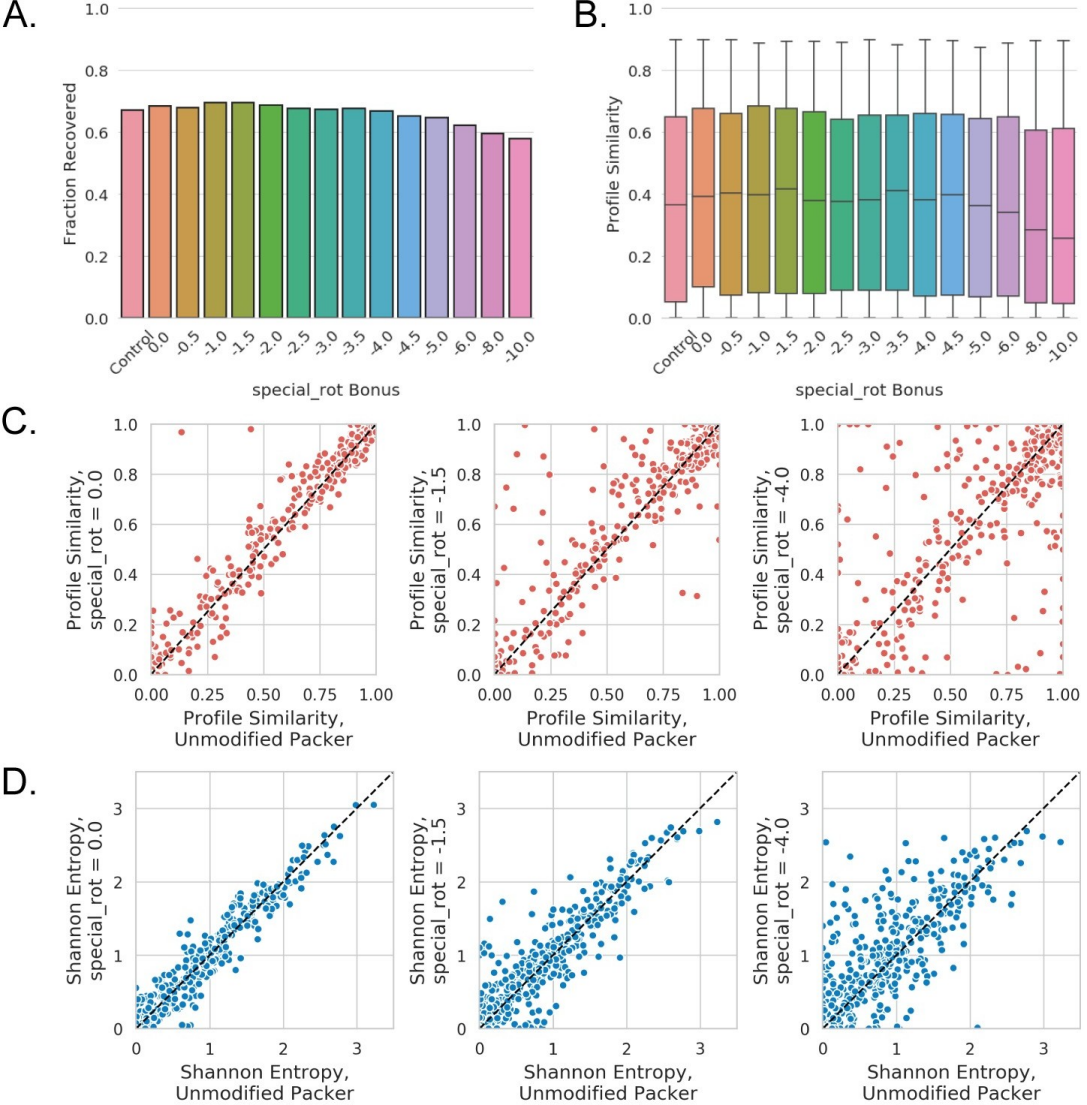
Complementary Rotamers Improve Binding Site Sequence Recovery. A. The fraction of designable positions in the benchmark complex set where the correct (wild-type) residue identity was incorporated at least 50% of the time (native sequence recovery) for a range of special_rot bonus values. Control indicates native sequence recovery when using the unmodified Packer. B. Box and whisker plots for profile similarities at designable positions in the benchmark set, where designable positions that achieve a profile similarity of >0.9 regardless of modifications to the Packer have been removed (plots with all positions are shown in S2 Fig). Box and whiskers denote quartiles, where whiskers indicate minimum and maximum values, boxes indicate 25^th^ and 75^th^ percentiles, respectively, and the line indicates the median value. C. Scatter plots showing profile similarity for individual designable positions at various special_rot bonus values (left: 0; middle: -1.5; right: -4.0) compared to the unmodified Packer. D. Scatter plots showing shannon entropy for individual designable positions at various special_rot bonus values (left: 0; middle: -1.5; right: -4.0) compared to the unmodified Packer.

The application of complementary rotamers with the special_rot score term improves, or at least matches, native sequence recovery over the unmodified Packer for special_rot bonus values up to -4.0 REU in the benchmark set (Fig 4A), with a special_rot bonus of -1.5 REU providing optimal sequence recovery in this test. When considering the median profile similarity for all 589 designable positions across the 22 complexes in our benchmark set as a metric, it initially appears that the application of complementary rotamers do not provide a significant improvement to design (S2 Fig). Median profile similarity remains constant with a special_rot bonus between -0.5 and -4.0 REU, and is comparable to profiles generated using Rosetta’s unmodified Packer as well as the addition of complementary rotamers without the application of the special_rot score term. However, a significant fraction of designable positions achieved high profile similarity (>0.9) regardless of modifications to the Packer. These positions were removed from subsequent analysis to investigate the impact of complementary rotamers. With these positions removed, it becomes evident that the addition of complementary rotamers can indeed provide a modest improvement in median profile similarity above a special_rot bonus of -5.0 REU (Fig 4B). As with sequence recovery, a special_rot bonus of -1.5 REU appears optimal for median profile similarity in this test. Beyond a special_rot bonus of -4.0 REU, the addition of the complementary rotamers becomes detrimental for both the native sequence recovery and profile similarity metrics (Fig 4A and 4B). This behavior is expected as the special_rot bonus begins to outweigh penalties otherwise incurred due to unfavorable physical interactions (e.g. steric clashes penalized by the repulsive score term fa_rep).

To investigate the per-position contributions of the complementary rotamers, we compared position-specific profile similarities for all designable positions in our benchmark set for three special_rot bonus values to results using the unmodified Packer (Fig 4C). The addition of complementary rotamers alone (with a special_rot bonus = 0) does not have a notable impact on profile similarity, although two positions achieve >0.9 profile similarities with the complementary rotamers while the unmodified Packer only achieves profile similarities <0.5 for the same positions (Fig 4C, left). The improvements provided by the complementary rotamers become apparent with a special_rot bonus of -1.5 REU, where 56 of 589 design positions achieve >0.1 improvement in profile similarity with the complementary rotamers as compared to the unmodified Packer (Fig 4C, middle). It is important to note that the complementary RotamerSet with a special_rot bonus of -1.5 REU provided an improvement in profile similarity to design positions without diminishing high-similarity positions in both Packer conditions. On the contrary, for special_rot bonus values of -4.0 REU and below, profile similarities deteriorate for positions originally recovered by the unmodified Packer (Fig 4C, right).

**Fig 5:**
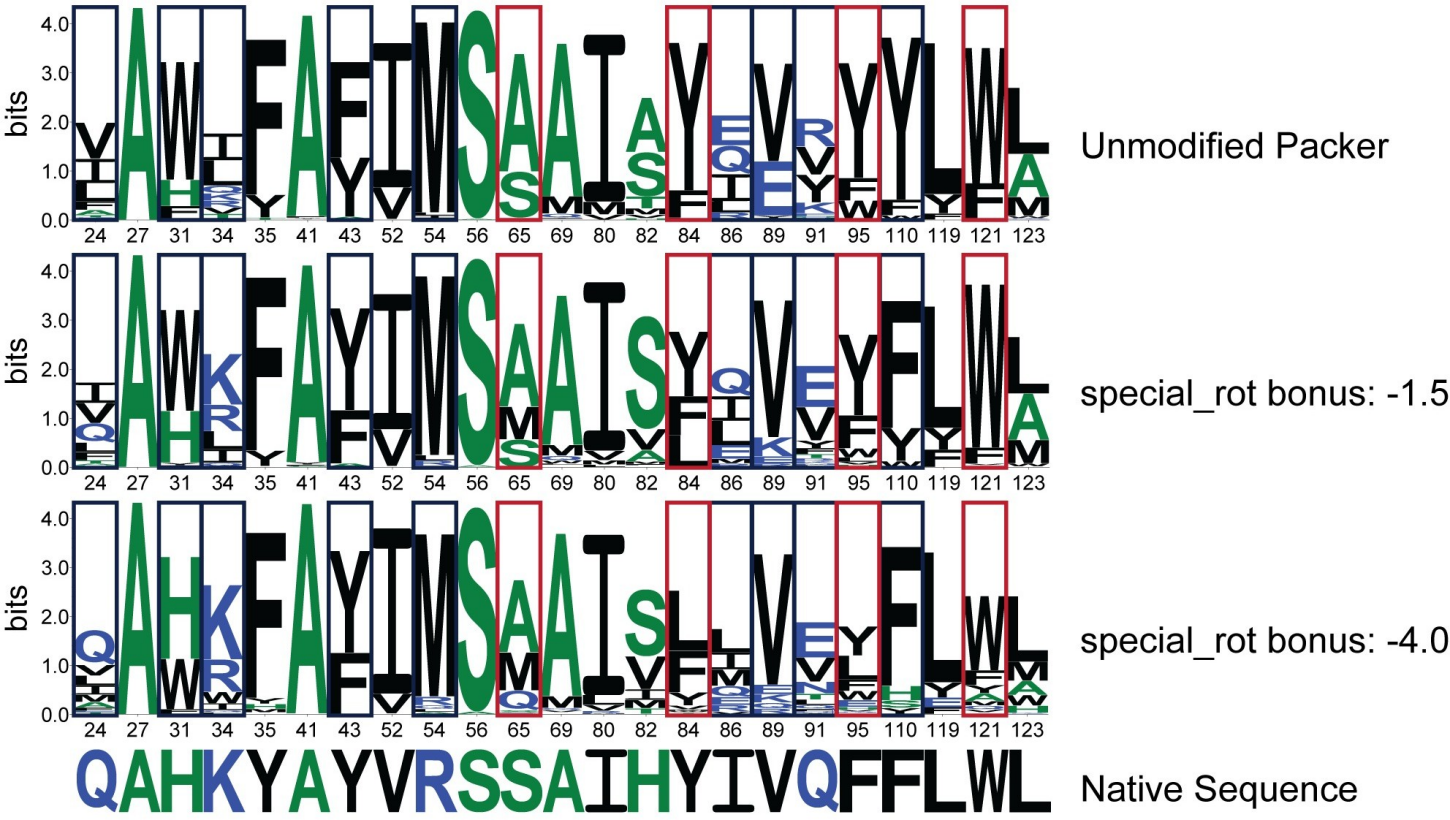
Sequence Logos for Recovery of Designable Binding Site Positions. Binding site recovery is shown for the engineered lipocalin DigA16 in complex with digitoxigenin (PDB ID: 1LNM, Ligand chemical component identifier: DTX). Sequence logos depict positional information content in bits, where residue identities are colored based on hydrophobicity (hydrophilic, blue; neutral, green; hydrophobic, black). Designable positions are in Rosetta numbering as in S4 Table. Design positions in navy blue boxes show improved native sequence recovery with the application of complementary rotamers. Specifically, positions with polar or charged residues show increased recovery with increased magnitude in the special_rot bonus. While the native residue identity of position 91 is not found, an isosteric residue is recovered with the application of complementary rotamers. Sequence recovery for positions in red boxes is negatively affected with increased special_rot bonus when compared to the unmodified Packer.

An additional benefit provided by complementary rotamers becomes evident when considering sequence entropy at designable positions: inclusion of complementary rotamers with a non-zero special_rot bonus leads to increased Shannon entropy for designable positions as compared to the unmodified Packer (Fig 4D), while maintaining comparable if not better median profile similarities up to a value of -4.0 REU for the special_rot bonus (Fig 4B). A key benefit of increased sequence entropy with complementary rotamers is demonstrated by the frequency and variety of polar and charged residues incorporated at several design positions (Fig 5, S1 Appendix). This behavior could lead to improvements over Rosetta’s known propensity to incorporate small, hydrophobic residues over side chains capable of mediating hydrogen bonds, leading to frequent issues with buried unsatisfied hydrogen bonding donors and acceptors and poor sequence recovery in polar binding sites [37].

### Composite binding sites and complementary rotamers applied to *De Novo* design

Finally, we sought to demonstrate the combined application of the methods outlined in this work toward improved design of protein-ligand interfaces *de novo*. We selected 5 ligands from our match comparison set and selected matches for these ligands for design that passed designability filters based on ligand burial, composite binding site energy, and potential hydrogen bond satisfaction with the ligand (S5 Table, S4 File). We then designed the binding site environment of the selected matches using complementary rotamers. While a special_rot bonus of -1.5 was found to provide the greatest benefit in terms of native sequence recovery and profile similarity in the binding site recovery benchmark (Fig 4A and 4B), we decided to also attempt complementary rotamers design with a special_rot bonus of -4.0. to investigate whether increased sequence entropy provided by a greater magnitude special_rot bonus (Fig 4D) would benefit design (and the benchmark metrics remained comparable to the unmodified Packer up to this bonus). We also used a special_rot bonus of -3.0 as a midpoint between the aforementioned conditions.

A total of 5000 trajectories were attempted for each design condition and outputs were passed through commonly applied Rosetta filters to determine the quality of designs. In particular, the quality of the designs was judged based on shape complementarity of the binding site with the ligand [41], packing within the binding site based on Rosetta’s Holes [42] filter, and ligand solvent accessible surface area (SASA). These metrics were selected as tightly packed binding sites with high shape complementarity to the ligand were previously demonstrated to be essential for high ligand binding affinity and specificity in *de novo* designed proteins [20].

The application of complementary rotamers improved key quality metrics when compared to design with the unmodified Packer for all special_rot bonus values attempted. Surprisingly, quality metrics improved even when the special_rot score bonus was set to zero (i.e. complementary rotamers were included but not favored beyond their Rosetta energies). This behavior is likely due to the increased degrees of sampling at the χ_1_ and χ_2_ torsions for complementary rotamers that were introduced to the Packer, resulting in finer sampling of rotamer conformations at the protein-ligand interface. The greatest benefit was imparted with a special_rot bonus of -3.0, where packing based on the Rosetta Holes score improved in 9/14 of design cases, ligand burial improved in 12/14 of design cases, and shape complementarity with the ligand improved in 11/14 of design cases (Table 2, S6 Table).

**Table 2:** Improved Design Metrics when Using Complementary Rotamers on Composite Binding Site Matches for 14 *De Novo* Ligand Binding Site Design Cases.

Special_rot bonus	Ligand SASA	RosettaHoles	Shape Complementarity
0	6	9	10
-1.5	7	8	11
-3.0	12	9	11
-4.0	12	8	9

Counts out of 14 cases of design on composite binding site matches where median metrics across 5000 designs that utilized composite RotamerSets with special_rot bonus values were improved as compared to the unmodified Packer. Ligand SASA and RosettaHoles are considered improved if the median decreased relative to the unmodified Packer. Shape complementarity is considered improved if the median increased relative to the unmodified packer.

**Fig 6:**
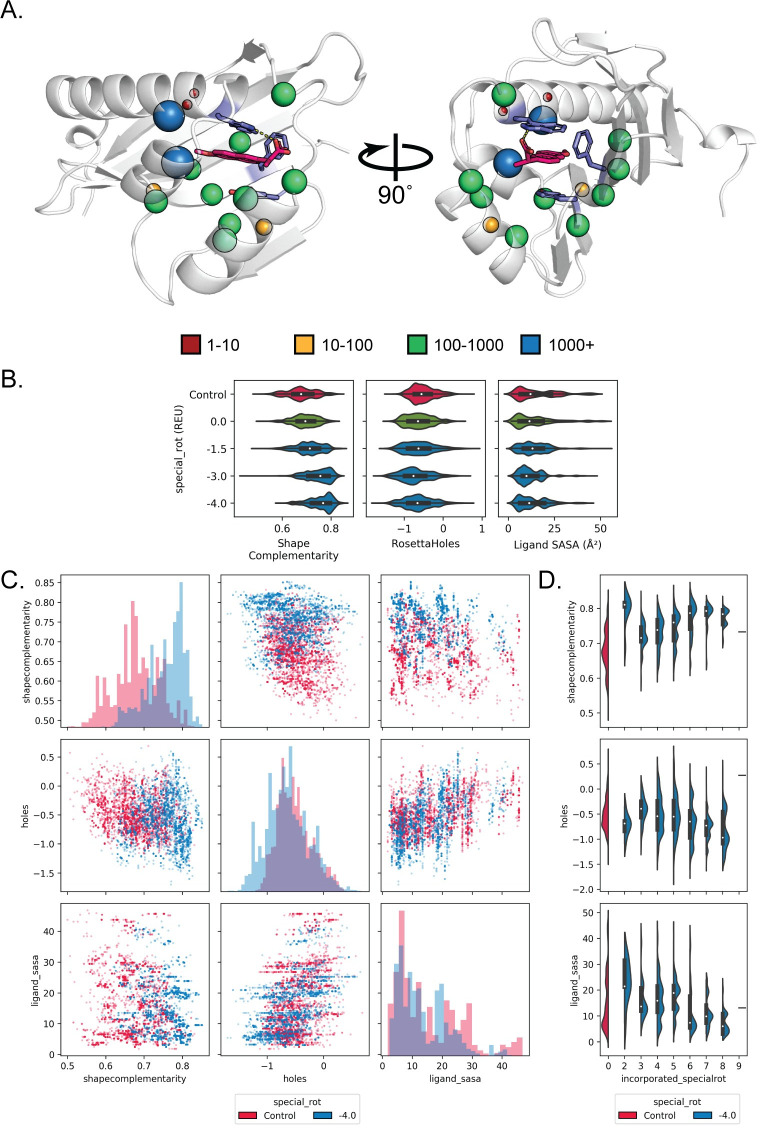
Composite binding sites combined with complementary rotamers improve design quality metrics for naproxen. A. Spheres indicate the number of complementary rotamers accepted per position to design the context of a composite binding site composed of tryptophan, tyrosine, and phenylalanine (purple) for naproxen (hot pink) placed in the scaffold protein PDBID 2RCD. B. Violin plots depict the distribution of values obtained for shape complementarity, RosettaHoles, and ligands SASA metrics after design using complementary rotamers with special_rot bonus values (blue; distributions after merely adding complementary rotamers with no special_rot bonus are shown in green). Control designs (red) were generated using the unmodified Packer. C. Scatterplots comparing different design metrics for individual designs (dots), showing simultaneous improvement in shape complementarity and binding site packing with the application of complementary rotamers (blue, special_rot bonus of -4.0 REU) compared to control simulations (red)). Histograms on the diagonal show the design metric distribution for control designs as compared to designs with complementary rotamers and a special_rot bonus of -4.0 REU. D. Violin plots showing the distributions of each design metric as a function of incorporated complementary rotamers.

**Fig 7:**
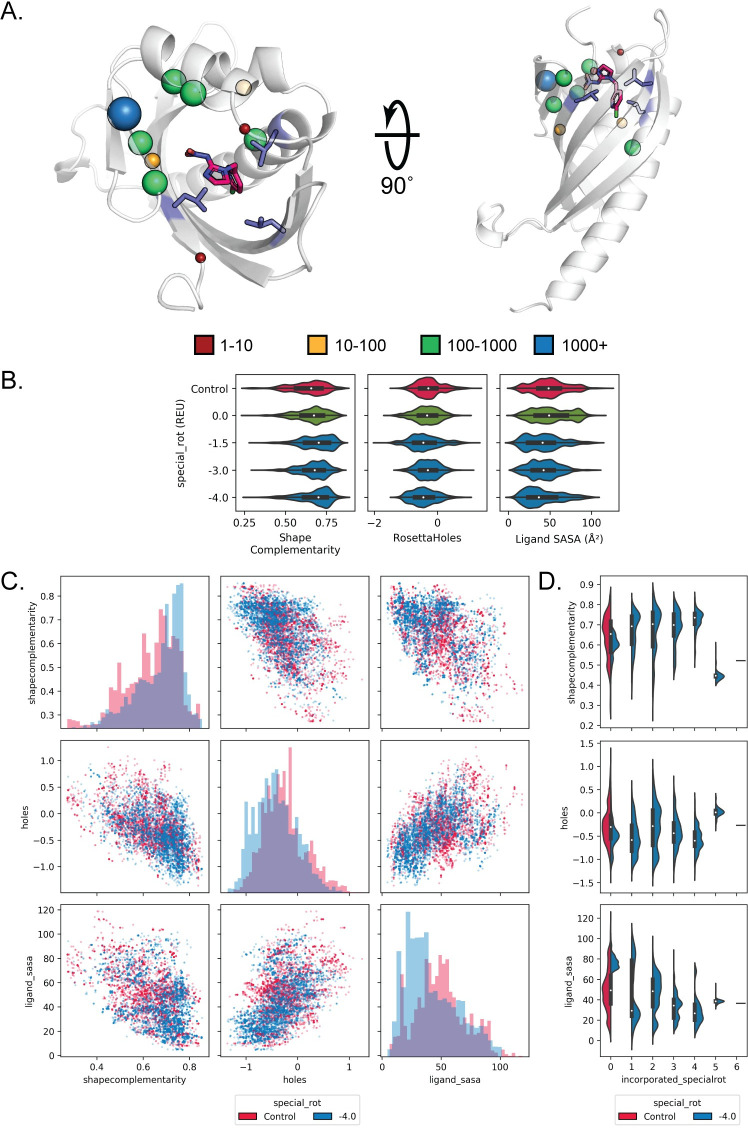
Composite binding sites combined with complementary rotamers improve design quality metrics for (E)-imidacloprid. A. Spheres indicate the number of complementary rotamers accepted per position to design the context of an all-leucine composite binding site (purple) for (E)-imidacloprid (hot pink) placed into the scaffold protein PDBID 1JKG. B. Plots and design metrics are as in Fig 6. Application of complementary rotamers improve shape complementarity and ligand SASA with increased special_rot bonus. C. Plots and design metrics are as in Fig 6. Designs show simultaneous improvement in shape complementarity, ligand SASA, and binding site packing with the application of complementary rotamers. D. Violin plots showing the distributions of each design metric as a function of incorporated complementary rotamers.

When we focused on design metrics for individual composite binding site matches, we found that complementary rotamers provided a considerable benefit. For instance, we attempted design on a composite binding site for naproxen consisting of tryptophan, tyrosine, and phenylalanine matched into the cavity of an NTF2-like protein (PDB ID: 2RCD) (Fig 6A). All design metrics improved compared to the unmodified packer when complementary rotamers with a special_rot bonus of -1.5 were applied (Fig 6B, shape complementarity increased and packing around the ligand improved with decreased Rosetta Holes scores and decreased ligand SASA values). In particular, increasing the special_rot bonus for complementary rotamers (more negative values) enriched for designs that possess high (>0.75) shape complementarity with naproxen. Moreover, application of complementary rotamers did not merely improve one design metric at the expense of other metrics (Fig 6C). For example, designs generated with complementary rotamers had both increased shape complementarity and decreased Rosetta Holes scores (blue dots in Fig 6C, top row middle plot), compared to designs generated by the unmodified Packer (red dots). Overall similar results were found in a composite binding site match for (E)-imidacloprid (Fig 7). Here, a composite binding site for (E)-imidacloprid consisting of three leucines was matched into the cavity of the TAP-p15 mRNA nuclear export factor (PDB ID: 1JKG). For both the imidacloprid and naproxen binding site design cases, design metrics typically improve with increasing numbers of complementary rotamers incorporated into the designs (Figs 6D and 7D). When taken together, these results demonstrate that applying complementary rotamers to design of composite binding site matches generates designs for *de novo* ligand binding sites that exhibit favorable metrics that have been previously shown to be predictive of design success.

## Discussion

In this work, we demonstrate new methods to improve the design of protein-ligand interfaces based on interactions observed in the PDB. By decomposing a small molecule into fragments, we leverage the wealth of structural information in the PDB to assemble pools of protein side chain interactions with fragments that compose the target ligand. We outline a new method to combine these discrete protein-fragment contacts into hundreds of thousands of composite binding sites for target ligands that are incorporated with high precision into scaffold proteins to nucleate a new binding site. We also outline a new method to bias the incorporation of side chain rotamers that reconstitute known interactions with ligand fragments during design. Finally, we demonstrate that the combination of these methods improves the quality of designs compared to Rosetta’s conventional design machinery as determined by commonly used design quality metrics.

The methods outlined in this work serve as a foundation for more complex strategies to design functional protein-ligand interactions. Protocols such as Rosetta’s FastDesign, which incorporates a small amount of backbone change into the design process by iterating between fixed-backbone design and fixed-sequence minimization steps, could include the complementary rotamers described here to improve protein-ligand interface design. Other flexible design methods such as CoupledMoves [43] or Backrub Ensembles [44] may benefit from the application of complementary rotamers to further increase design sequence diversity and incorporation of key protein-ligand interactions in a binding site. Similarly, complementary rotamers could be incorporated into other side chain design approaches [45–47].

While we were able to demonstrate effective application of protein-fragment interactions to the design of ligand binding sites, there are several classes of contacts that were not used productively. For instance, we noticed that 19.3% of fragment interactions in the contact pools generated for the ligands in this work were found to be mediated by backbone atoms (C, CA, O, N). This proportion agrees with the binding sites in the match comparison benchmark, where 17 of 93 contact constraints generated for existing complexes (see Methods) were mediated by a backbone atom. However, our composite binding site method focused on introducing productive side chain interactions with the ligand. In addition, complementary rotamers would not benefit from the addition of backbone interactions since they may not be introduced once the ligand is placed within a potential binding site context. It is also well known that water-mediated interactions are frequent in polar protein-small molecule interfaces [48,49] but are not considered here. Updating the method to better utilize these interactions could improve our ability to design functional protein-ligand interactions in the future.

We empirically found that three-residue composite binding site definitions provide a practical compromise between optimizing favorable interactions with a given ligand and maximizing the set of scaffolds that could in principle accommodate a binding site for the ligand. Two-residue binding site definitions could be matched into many scaffolds but only a small subset of these may yield well-packed binding sites with high shape complementarity to the ligand even after design. On the other hand, given the limited set of backbone conformations composed by the scaffold proteins in our benchmark, only few or no binding site definitions with more than 3 residues can be accommodated by a scaffold while fulfilling all constraints and passing all quality filters. We note, however, that our algorithm can in principle be applied to more than 3 residues (limited by the total surface area of the target ligand). Using larger composite binding sites could be advantageous if the number of available scaffold backbone geometries can be increased substantially for example by engineering families of *de novo* designed proteins with tunable shapes.

Design with complementary rotamers relies on a fixed bonus provided by the special_rot score term to bias incorporation of observed protein-fragment interactions. While a special_rot bonus of -1.5 was found to provide the most benefit in native sequence recovery and profile similarity across all complexes in our benchmark set, the most adequate bonus to maximize these metrics varied between individual complexes. In addition, we found several design positions in the binding site recovery benchmark where the application of complementary rotamers was detrimental to sequence recovery (see examples in Fig 5). More tunable methods of applying bonuses for each complementary rotamer incorporated during design may provide a more generalizable benefit across varying design contexts. For instance, the design centric guidance terms implemented in Rosetta could provide a ramping score that provides a significant bonus for the first complementary rotamer incorporated, then ramp down the bonus for each additional rotamer introduced [50]. These metrics could also be implemented to bias incorporation of complementary rotamers that satisfy certain user-defined constraints such as hydrogen bonding with the ligand.

The methods described in this work provide a new framework for the *de novo* design of small molecule binding sites into proteins and directly address key shortcomings in current state-of-the-art methods for protein-ligand interface design. We drastically expand the possible combinations of protein-ligand contacts that can be incorporated into scaffold proteins by recombining observed protein-fragment contacts into composite binding sites, paving the way for the computational design of new binding sites for targets for which no co-complex structure exists in the PDB. To complement shortcomings in Rosetta’s energy function with regards to ligands, we also apply contact pools to bias the design of protein-ligand interfaces toward contacts that are frequently observed to mediate interactions with defined ligand substructures in the PDB. When combined with Rosetta’s success in *de novo* protein designed protein structures, we provide a foundation for the complete *de novo* design of functional ligand-binding proteins. For instance, where most existing methods attempt to build a binding site into an existing scaffold, just as we have demonstrated with three-residue composite binding sites and RosettaMatch, composite binding sites of arbitrary size can instead serve as a scaffold to build functionalized *de novo* proteins from the inside-out. A significant challenge here is building secondary structure from the defined binding site residues to form a stable tertiary structure. The SEWING method [51,52] accomplishes this for metal-coordination sites by combining elements of existing proteins that fulfill geometric constraints imposed by the coordination center to design a functional chimeric protein. However, this method has yet to be used to model proteins with non-metal ligands. It would also be interesting to apply loop modeling and design methods [53] for precisely positioning functional residues or repositioning binding site residues that deviate from constraints.

While experimental characterization provides the ultimate test for the methods described here, we aimed to provide computational benchmarks that evaluate the performance of the method on a broad number of test cases (whereas experimental characterization would necessarily be limited to one or a few systems). We hope that disseminating this work will allow others to apply these methods to their own protein design problems. We envision that composite binding sites and complementary rotamers will pioneer the design of proteins tailor built for specific functions and will augment our ability to design catalysts, sensors, and inducible transcription factors that require complex understanding of how protein-ligand interactions mediate chemistry and allostery.

## Materials and methods

### Code availability

The collection, processing, and application of contact pools derived from observed protein-ligand fragment interactions in the PDB is implemented as a Python3 package called BindingSitesFromFragments (BSFF). BSFF builds upon the Rosetta Molecular Modeling Suite [18], PyRosetta (PyRosetta4.conda.linux.CentOS.python37.Release r240 2019.50+release.91b7a94) [54], ProDY [55], and RDKit [36]. Each step of the protocol is accessible through a command-line interface with minimal intervention by the user after generating the initial inputs. BSFF creates and automatically populates a directory tree named with a three-letter code (typically the ligand’s three character PDB identifier, referred to here as [/TGT]) in a user-defined location. All scripts and commands are included as part of the BSFF Repository (https://github.com/jaaamessszzz/BindingSitesFromFragments). Additional scripts for generating figures and submitting jobs are also available (https://github.com/jaaamessszzz/BindingSitesFromFragments-Utilities).

### Prepare input files

BSFF requires a Sybyl MOL2 representation of the target ligand as an initial input. For ligands with rotatable bonds, we used OpenEye Omega (version 2.5.1.4) [35] with default settings to generate a conformer library. A custom script (molfile_split_into_singles.py) uses Rosetta’s molfile_to_params.py to generate a Rosetta params file for each ligand conformer. PDB representations of each conformer are also generated by molfile_split_into_singles.py so that unique atom names are consistent across all ligand conformers. These files are written to [/TGT/Inputs/Rosetta_Inputs].

### Define fragments

Avogadro [56] is used to generate user-defined fragments (a subset of atoms from the target ligand) derived from a single PDB representation produced in the previous step. This step relies on the user’s chemical intuition to define fragments that represent local chemical substructures of the target ligand, which will be used to search the PDB to collect protein contacts with each defined fragment. Each ligand fragment is saved as a PDB file and must conserve the unique atom names found in the full PDB representation of the target ligand. Typically, 5–10 fragments consisting of 3–10 atoms each are generated for a target ligand. The PDB representations of each fragment should be saved as Fragment_X.pdb to [/TGT/Inputs/Fragment_Inputs], where each fragment should be enumerated (replacing X in Fragment_X.pdb with fragment index).

### Search for protein-fragment interactions

PubChem substructure search [57] is used to identify small molecules that possess user-defined fragments as substructures. For each fragment, the PDB representation generated in the previous step is imported into the PubChem Sketcher Tool and converted into a SMILES string. Atom query attributes are defined to restrict search results to small molecules that possess user-defined fragments as substructures with the same connectivity and local chemical environment (e.g. bond order, aromaticity) as the target molecule. Explicit hydrogens are not removed before searching. A molecular weight cutoff of 800Da is applied to the search results. The top 1,000,000 search results are downloaded as a CSV file (cid, inchikey) and saved as Fragment_X.csv to [/TGT/Inputs/Fragment_Inputs], where the CSV name should correspond to the input fragment PDB. Complete SMILES search queries for each fragment are to be enumerated and saved in Fragment_Inputs.csv found in [/TGT/Inputs/Fragment_Inputs].

For each fragment, an intersection of PubChem search results with a complete list of ligands represented in the PDB from LigandExpo [58] yields all fragment-containing small molecules that may be found in complex with a protein in the PDB. For each fragment-containing molecule, the PDB REST API returns all structures with a 90% sequence identity cutoff where the small molecule is in complex with a protein (all complexes containing DNA/RNA are excluded). Ligands and protein-ligand complexes for each fragment are stored under [/TGT/PDB_search_results.json].

Alternatively, the Chemical Component Search function available through the PDB can be used to populate [/TGT/PDB_search_results.json]. Using this function may find ligands that are missed by PubChem, but at the expense of limitations in the ability to define fragment chemical environments in the search query.

### Align

All protein-ligand complexes found in the previous step are processed to consolidate protein contacts in reference to each user-defined fragment. For each fragment-containing ligand in a protein-ligand complex, the structure is checked to verify that 1) the ligand does not possess multiple occupancies and 2) all ligand atoms are resolved. If the structure passes these quality checks, the fragment substructure in the ligand is identified using SMILES representations and the Maximum Common Substructure search as implemented by RDKit. For each substructure contained within a ligand, all protein atoms within 12Å of the fragment substructure (including the fragment) are extracted and transformed such that the identified substructure is superimposed onto the user-generated PDB representation of the fragment. This process produces an aligned ensemble of all protein contacts with the defined fragment in the PDB that pass all quality filters.

### Cluster

Agglomerative hierarchical clustering is performed for each fragment’s ensemble of protein contacts to identify highly represented protein-fragment contacts. A feature vector is generated for residues in the ensemble consisting of: spatial position relative to the fragment, contact distance, interaction chemistry, and residue identity (S7 Table). A filtering step removes hydrophobic residues (ACFGILMPVW) >4Å from any fragment atom or polar residues (DEHKNQRSTY) >3.5Å from any fragment atom. Residues are first clustered by protein-fragment interaction chemistry (Hamming distance, cutoff = 2, linkage = complete) followed by spatial distribution about the fragment (Cosine distance, cutoff = 1—cos(20*π/180), linkage = average) to generate clusters of residues that mediate similar interaction types with specific parts of the target fragment.

### Fragment contact analysis

Bootstrapping was applied to approximate the proportion of protein-fragment contact clusters (i.e. contact modes) that would be recovered given an observed fraction of the PDB. For a given fragment and sample size (ranging from 0% to 100% of the PDB at 5% intervals), 1000 samples were drawn from the PDB and the number of recovered clusters was recorded. Clusters were considered recovered if at least one contact in the cluster was sourced from a PDB in the sample. The average fraction and standard deviation of clusters recovered were reported for each fragment and sample size. Results for select fragments are shown in Fig 2 and for all considered fragments in S1 Fig.

### Contact pool assembly

All clustered protein-fragment contacts are positioned relative to their source fragments in the target ligand to produce an ensemble of aligned protein contacts with the full target ligand. For ligand conformers, side chains are transformed using three atoms each on the ligand and side chain to maintain the contact geometry observed in the PDB. Additional filters are applied to remove redundant contacts and remove contacts from structures with missing (zero occupancy) protein atoms or alternative protein conformations (residues with ALTLOC records). Contacts are considered redundant if a side chain has already been accepted with equivalent side chain contact atoms, equivalent ligand contact atom, side chain contact atoms RMSD ≤0.5Å, and Cα atom distance ≤1.5Å. Contact pools are limited to the best 5000 contacts as determined by Rosetta energy. Furthermore, the addition of hydrogen bond donors/acceptors (as determined by a negative hbond_sc score for the contact) to the contact pool are prioritized (up to 500 residues per donor/acceptor atom on the ligand) and remaining contacts are equally distributed about the ligand based on the ligand atom mediating contact with each residue. Alanine, glycine, proline, and cysteine residues were not added to contact pools in this work.

### Assembly of composite binding sites

For each target ligand conformer, a Monte Carlo protocol was applied to assemble discrete side chains in the contact ensemble into composite binding sites. A composite binding site is initialized with three random side chains from the contact ensemble and select two-body score terms in the Rosetta full-atom energy function (fa_rep, fa_atr, fa_elec, hbond_sc, fa_sol) are used to evaluate the overall energy of the binding site. During each move, a side chain in the current binding site is replaced with a random side chain from the contact ensemble. The new binding site is scored and the move is kept based on the Metropolis criterion, where the objective is to minimize the Rosetta energy of the composite binding site. The temperature is ramped down geometrically across seven steps. Up to ten trajectories are performed for each contact ensemble and the lowest energy 100,000 solutions discovered per trajectory are recorded.

### Selection of binding site definitions for existing complexes

Binding site definitions were generated for existing protein-ligand complexes in the Match comparison benchmark. These constraints consist of the three lowest-energy contacts in the binding site for the given complex in terms of the two-body Rosetta energies used to assemble contact pools (fa_rep, fa_atr, fa_elec, hbond_sc, fa_sol) and the hbond_bb_sc term to account for binding site backbone contacts with the ligand. Existing protein-ligand complexes were relaxed with the Rosetta FastRelax protocol with the full REF2015 energy function and starting coordinate constraints (FastRelax command line option -relax:constrain_relax_to_start_coords) before determining constraint contacts.

### Match binding sites into scaffolds

RosettaMatch [19] is used to find binding site solutions that may be accommodated by existing backbone geometries in a protein scaffold set. A monomeric scaffold set was previously assembled [20]. RosettaMatch constraint files are automatically generated for the best 5,000 binding site solutions across all ligand conformers in terms of objective value (Rosetta energy). Constraint files are generated for the RosettaMatch algorithm using a custom script that calculates six degrees of freedom (1 distance, 2 angles, 3 torsions) for three ligand atoms and three side chain atoms that mediate the ligand–protein side chain interaction. Each angle and torsion degree of freedom is sampled at ±5° in addition to the ideal value, while the distance is fixed at the ideal value. Additional flags for the RosettaMatch application are as follows: -ex1 -ex2 -extrachi_cutoff 0 -bump_tolerance 0.5 -euclid_bin_size 1 -euler_bin_size 10 -match:consolidate_matches -match_grouper SameRotamerComboGrouper -output_matches_per_group 1.

Successful matches must pass additional filters where 1) the binding site energy as calculated in the context of the scaffold < 0 Rosetta energy units (REU), 2) max fa_rep for any motif residue < 25 REU, and 3) max fa_sol for any motif residue < 5 REU. A custom “hydrogen bond satisfaction” filter is also applied to ensure that positions exist in the match scaffold where a side chain can be built to satisfy all potential hydrogen bond donor/acceptor atoms on the ligand. This filter generates rotamers (-ex1 -ex2 -extrachi_cutoff 0) for all positions within 10Å of the ligand. The filter reports which hydrogen bond donor/acceptor atoms on the ligand are satisfied for all contacts found where hbond_sc < 0, fa_rep < 10, and the sum of two-body terms used to solve for composite binding sites < 10.

### Selection of binding site recovery benchmark set

Protein-ligand complexes for the binding site recovery benchmark were identified using the BindingMOAD [40] with the following search criteria: binding class, ligand mass of 200–800 Da, <1uM dissociation constant (K_D_), and crystal structure of <2.5Å resolution. In addition to these criteria, complexes were further curated for binding sites composed of mainly side chain interactions, ligands with less than six rotatable bonds, and few waters and no metal coordination in the binding site. A set of 22 protein-ligand complexes was identified for the benchmark (S3 Table, S3 File). PDB ID 6M9B was used in place of BindingMOAD annotated streptavidin-biotin complexes.

### Generation of complementary rotamers

Complementary rotamers are generated per-position in a protein-ligand interface to augment the set of rotamers Rosetta’s Packer will use to design the binding site. Complementary rotamers are created in two steps. First, a new contact pool is assembled for the ligand placed into the scaffold in the protein-ligand complex. In addition to the aforementioned criteria for contact pool assembly, inverse rotamers [19] are generated for potential contacts in the context of the ligand binding site and are only added to the new contact pool if an inverse rotamer’s mainchain (C, CA, N) RMSD to any position in the protein backbone is 2Å or less. This process yields a contact pool for interactions that may potentially be recapitulated within the context of the protein-ligand interface. Second, rotamers are generated for designable positions in the protein-ligand interface with extra χ-angle sampling at two half-step standard deviations for χ_1_ and χ_2_ (-packing:ex1:level 4 -packing:ex2:level 4) and one standard deviation for χ_3_ and χ_4_ (-packing:ex3:level 1 -packing:ex4:level 1). Rotamers that recapitulate contact geometries observed in the target ligand contact pool with RMSD ≤ 1.5Å are added to Rosetta’s Packer RotamerSets. By default, up to 50 rotamers are added per residue identity to a position’s RotamerSet, where each rotamer is flagged with the SPECIAL_ROT VariantType. If more than 50 rotamers are found to recapitulate contact pool interactions for a given position and residue identity, only the best rotamers by Rosetta energy are added to the RotamerSet.

### Design with complementary rotamers

Protein-ligand complexes (e.g. matches, or existing complexes from the PDB) are passed to conventional Rosetta design methods to redesign the binding site environment with the aid of complementary rotamers. Matches are relaxed using the FastRelax protocol with starting coordinate constraints before generating complementary rotamers and sequence design. The binding site environment is designed using fixed backbone packing as implemented in Rosetta [25] where the Packer RotamerSets are augmented with complementary rotamers flagged with the SPECIAL_ROT VariantType. All protein positions within 10Å of the ligand with Cα-Cß vectors pointing toward the ligand (i.e. dot product of Cα-Cß vector and Cα-centroid vectors > 0) define a first shell of contacts that are set to designable (i.e., allowed to change residue identity). In addition, these positions are passed to the ClashBasedShellSelector to identify a second shell of residues that are also set to designable. All designable positions are again passed to the ClashBasedShellSelector to identify a third shell of residues that are set to repackable (i.e., allowed to change conformation while keeping the residue identity the same). All other positions in the protein are fixed during design. All cysteines, glycines, and prolines in the protein are fixed. The following flags were used for design: -ex1 -ex2 -extrachi_cutoff 0 -use_input_sc -run:preserve_header -extra_res_fa {params_path} -total_threads 1, where {params_path} is the path to the params file for the ligand in the complex. Up to 10,000 independent design simulations are performed for each match with the special_rot score term added to the default REF2015 energy function to bias incorporation of complementary rotamers during design. For *de novo* binder design, matched binding site residues are set to repack only and constraints as defined during matching are applied using the AddOrRemoveMatchCsts mover to maintain contact geometries with the ligand during design. Rosetta filters were used to compute shape complementarity [41], RosettaHoles score [42], and ligand solvent accessible surface area metrics. The Binding Strain, Residue Interaction Energy, PackStat, and BuriedUnsatHbonds filters were also computed, but were less informative (S2 Appendix). An additional entry for chlorine (radius of 1.75Å) was added to the shape complementarity atom radius database file [database/scoring/score_functions/sc/sc_radii.lib] for ligands that contain chlorine atoms.

### Profile similarity and sequence recovery

For the binding site recovery benchmark, profile similarity was calculated at each design position as previously described [43], where profile similarity is defined as 1—Jensen-Shannon Divergence [59]. Jensen-Shannon Divergence was calculated per position for design sequences against the native complex sequence taken from the PDB. For sequence recovery, design positions were considered recovered if >50% of design sequences incorporated the residue identity observed in the native complex.

## Supporting information

S1 File Top5000 RosettaMatch constraints for ligands in match comparison. RosettaMatch constraints (.cst) generated for the 5000 lowest-energy composite binding site solutions found for application ligands listed in S2 Table and applied in the Match comparison benchmark.
(GZ) Click here for additional data file.

S2 File Match constraints for existing complexes in match comparison. RosettaMatch constraints (.cst) generated for existing complexes in the PDB for ligands in the Match comparison benchmark.
(GZ) Click here for additional data file.

S3 File PDBs for binding site recovery benchmark. PDB files for the protein-ligand complexes used for the binding site sequence recovery benchmark. Files have been cleaned to remove residues with missing backbone atoms and all other extraneous small molecules. These complexes have been renumbered for compatibility with Rosetta, where position numbering corresponds to column “Designable_positions” in S4 Table.
(GZ) Click here for additional data file.

S4 File Composite binding site definition, RosettaMatch constraint file, and PDBs for matches carried forward through design. PDB files for matches that were selected for forward design, the RosettaMatch constraint files (.cst) that yielded the match, and corresponding source composite binding site definition (.pdb) that was used to generate the RosettaMatch constraint file.
(GZ) Click here for additional data file.

S1 Appendix Sequence logos for binding site benchmark complexes at various special_rot bonus values. Sequence logos generated for designable positions in existing complexes listed in S4 Table for various special_rot bonus values, where each logo represents 5000 design trajectories. A special_rot bonus of 0 indicates that complementary rotamers were added to the Packer, but received no score term bias. “Control” sequence logo was generated using the unmodified Packer. “Native” sequence logo shows the “correct” residue identity found in the native protein-ligand complex. Sequence logo positions are in Rosetta numbering corresponding to PDB files provided in S3 File.
(PDF)

S2 Appendix Plots for all design metrics measured during forward design. Design metric distributions measured for 5000 forward design trajectories of selected matches listed in S5 Table. Existing Rosetta filters were applied to calculate binding strain (bindingstrain), interaction energy between the protein and ligand (residueie), packing statistics (Packstat), shape complementarity between the protein and ligand (shapecomplementarity), packing with RosettaHoles (holes), and the number of buried unsatisfied hydrogen bond donors/acceptors (heavyburiedunsats) for each design. Ligand solvent accessible surface area in Å^2^ (ligand_sasa), number of hydrogen bonds made between the protein and ligand (hbond), and number of complementary rotamers incorporated during design (incorporated_specialrot) were also calculated.
(PDF)

S1 Table Statistics for fragments used to investigate contact diversity. Kekule structures and corresponding SMILES strings are provided for all fragments used to investigate the availability of protein-fragment contact information in the PDB. The total number of unique residue-fragment contacts and the number of unique clusters (i.e. contact modes) represented by these residue-fragment contacts are reported.
(DOCX)

S2 Table Application ligands. Full name and chemical component identifier for ligands used for the Match comparison benchmark and forward design with matched composited binding sites. All existing PDBs where a protein creates a contact interface with the ligand are provided.
(DOCX)

S3 Table BindingMOAD complexes used for binding site sequence recovery. PDB ID, PDB description, ligand chemical component identifier, and full ligand name for BindingMOAD protein-ligand complexes applied in the binding site recovery benchmark.
(DOCX)

S4 Table Binding site benchmark design details. Statistics for complementary rotamers generated and applied to the binding site recovery benchmark. Rosetta numbering and the corresponding PDB numbering for scaffold PDBs (as in S3 File) are provided. Here we report the number of designable positions as well as the number of complementary rotamers generated, accepted (i.e. passed Rosetta energy and RMSD filters, see Methods), and applied to design for each benchmark binding site. Only the best 50 rotamers per residue type per position were applied top design.
(DOCX)

S5 Table Forward design complexes design details. Statistics for complementary rotamers generated and applied to forward design of complexes generated with RosettaMatch for application ligands. Match scaffold PDB ID, scaffold description, complex designable positions in Rosetta numbering, ligand chemical component identifier, and constraint file that yielded the match (as in S4 File) are provided. We also report the number of designable positions as well as the number of complementary rotamers generated, accepted (i.e. passed Rosetta energy and RMSD filters, see Methods), and applied to design for each benchmark binding site. Only the best 50 rotamers per residue type per position were applied top design.
(DOCX)

S6 Table Counts for improved metrics across all attempted designs. Counts out of 14 composite binding site matches where median metrics across 5000 designs that utilized composite RotamerSets with special_rot bonus values were improved as compared to designs generated with unmodified Packer. PackStat and shape complementarity are considered improved if the median increased relative to the unmodified Packer. Ligand SASA, binding strain (bindingstrain), residue interaction energy (residueie), and RosettaHoles are considered improved if the median decreased relative to the unmodified packer. Number of hydrogen bonds made with the ligand (hbonds) is considered improved if the median count is more than the median count for the unmodified Packer. Heavy buried unsatisfied hydrogen bond donor/acceptor count (heavyburiedunsats) is considered improved if the median count is less than the median count for the unmodified Packer.
(DOCX)

S7 Table Feature vector components. Descriptions of individual feature vector components used to cluster residue-fragment interactions into unique contact modes using hierarchical agglomerative clustering.
(DOCX)

S1 Fig Fraction of PDB required for >80% contact recovery across all tested fragments. Counts for the fraction of the PDB required to achieve >80% contact recovery for all 34 tested fragments (as in S1 Table).
(PNG)

S2 Fig Profile similarity without >0.9 positions removed. Profile similarities as depicted in Fig 4B, except profile similarities for all designable positions are included.
(PNG)

## References

[1] Huang PS, Boyken SE, Baker D. The coming of age of de novo protein design. Nature. 2016 9 14;537(7620):320–7. 10.1038/nature19946 27629638

[2] Schreier B, Stumpp C, Wiesner S, Höcker B. Computational design of ligand binding is not a solved problem. Proc Natl Acad Sci U S A. 2009 11 3;106(44):18491–6. 10.1073/pnas.0907950106 19833875 PMC2773959

[3] Baker D. What has de novo protein design taught us about protein folding and biophysics? Protein Sci. 2019 4 1;28(4):678–83. 10.1002/pro.3588 30746840 PMC6423711

[4] Dou J, Doyle L, Greisen P, Schena A, Park H, Johnsson K, et al Sampling and energy evaluation challenges in ligand binding protein design. Protein Sci. 2017 12 1;26(12):2426–37. 10.1002/pro.3317 28980354 PMC5699494

[5] Lechner H, Ferruz N, Höcker B. Strategies for designing non-natural enzymes and binders Vol. 47, Current Opinion in Chemical Biology. Elsevier Ltd; 2018 p. 67–76. 10.1016/j.cbpa.2018.07.022 30248579

[6] Yang W, Lai L. Computational design of ligand-binding proteins Vol. 45, Current Opinion in Structural Biology. Elsevier Ltd; 2017 p. 67–73. 10.1016/j.sbi.2016.11.021 27951448

[7] Stiel AC, Nellen M, Höcker B. PocketOptimizer and the design of ligand binding sites In: Methods in Molecular Biology. Humana Press Inc.; 2016 p. 63–75. 10.1007/978-1-4939-3569-7_5 27094286

[8] Malisi C, Schumann M, Toussaint NC, Kageyama J, Kohlbacher O, Höcker B. Binding Pocket Optimization by Computational Protein Design. Levy YK, editor. PLoS One. 2012 12 27;7(12):e52505 10.1371/journal.pone.0052505 23300688 PMC3531388

[9] Barros EP, Schiffer JM, Vorobieva A, Dou J, Baker D, Amaro RE. Improving the Efficiency of Ligand-Binding Protein Design with Molecular Dynamics Simulations. J Chem Theory Comput. 2019 10 8;15(10):5703–15. 10.1021/acs.jctc.9b00483 31442033 PMC7532806

[10] Hallen MA, Martin JW, Ojewole A, Jou JD, Lowegard AU, Frenkel MS, et al OSPREY 3.0: Open-source protein redesign for you, with powerful new features. J Comput Chem. 2018 11 15;39(30):2494–507. 10.1002/jcc.25522 30368845 PMC6391056

[11] Shirvanyants D, Alexandrova AN, Dokholyan N V. Rigid substructure search. Bioinformatics. 2011; 10.1093/bioinformatics/btr129 PMC3138080 21460026

[12] Lombardi A, Pirro F, Maglio O, Chino M, DeGrado WF. De novo design of four-helix bundle metalloproteins: One scaffold, diverse reactivities. Acc Chem Res. 2019 4 11;52(5):1148–59. 10.1021/acs.accounts.8b00674 30973707 PMC7362765

[13] Polizzi NF, Wu Y, Lemmin T, Maxwell AM, Zhang SQ, Rawson J, et al De novo design of a hyperstable non-natural protein-ligand complex with sub-Å accuracy. Nat Chem. 2017 12 1;9(12):1157–64. 10.1038/nchem.2846 29168496 PMC5859929

[14] Scheib U, Shanmugaratnam S, Farías-Rico JA, Höcker B. Change in protein-ligand specificity through binding pocket grafting. J Struct Biol. 2014 2 1;185(2):186–92. 10.1016/j.jsb.2013.06.002 23792166

[15] De Los Santos ELC, Meyerowitz JT, Mayo SL, Murray RM. Engineering Transcriptional Regulator Effector Specificity Using Computational Design and in Vitro Rapid Prototyping: Developing a Vanillin Sensor. ACS Synth Biol. 2016 4 15;5(4):287–95. 10.1021/acssynbio.5b00090 26262913

[16] Lassila JK, Privett HK, Allen BD, Mayo SL. Combinatorial methods for small-molecule placement in computational enzyme design. Proc Natl Acad Sci U S A. 2006 11 7;103(45):16710–5. 10.1073/pnas.0607691103 17075051 PMC1636520

[17] Allen BD, Mayo SL. An efficient algorithm for multistate protein design based on faster. J Comput Chem. 2010 4 15;31(5):904–16. 10.1002/jcc.21375 19637210

[18] Leaver-Fay A, Tyka M, Lewis SM, Lange F, Thompson J, Jacak R, et al ROSETTA 3: An Object-Oriented Software Suite for the Simulation and Design of Macromolecules. Methods Enzymol Vol 487. 2011;487(11):545–74. 21187238 10.1016/B978-0-12-381270-4.00019-6 PMC4083816

[19] Zanghellini A, Jiang L, Wollacott AM, Cheng G, Meiler J, Althoff EA, et al New algorithms and an in silico benchmark for computational enzyme design. Protein Sci. 2006 12 1;15(12):2785–94. 10.1110/ps.062353106 17132862 PMC2242439

[20] Tinberg CE, Khare SD, Dou J, Doyle L, Nelson JW, Schena A, et al Computational design of ligand-binding proteins with high affinity and selectivity. Nature. 2013 9 4;501(7466):212–6. 10.1038/nature12443 24005320 PMC3898436

[21] Bick MJ, Greisen PJ, Morey KJ, Antunes MS, La D, Sankaran B, et al Computational design of environmental sensors for the potent opioid fentanyl. Elife. 2017 9 19;6. 10.7554/eLife.28909 PMC5655540 28925919

[22] Glasgow AA, Huang YM, Mandell DJ, Thompson M, Ritterson R, Loshbaugh AL, et al Computational design of a modular protein sense-response system. Science (80-). 2019 11 22;366(6468):1024–8. 10.1126/science.aax8780 31754004 PMC7343396

[23] Dou J, Vorobieva AA, Sheffler W, Doyle LA, Park H, Bick MJ, et al De novo design of a fluorescence-activating β-barrel. Nature. 2018 9 27;561(7724):485–91. 10.1038/s41586-018-0509-0 30209393 PMC6275156

[24] Yang W, Lai L. Computational design of ligand-binding proteins. Curr Opin Struct Biol. 2017 8 1;45:67–73. 10.1016/j.sbi.2016.11.021 27951448

[25] Kuhlman B, Baker D. Native protein sequences are close to optimal for their structures. Proc Natl Acad Sci U S A. 2000 9 12;97(19):10383–8. 10.1073/pnas.97.19.10383 10984534 PMC27033

[26] Shapovalov M V., Dunbrack RL. A smoothed backbone-dependent rotamer library for proteins derived from adaptive kernel density estimates and regressions. Structure. 2011 6 8;19(6):844–58. 10.1016/j.str.2011.03.019 21645855 PMC3118414

[27] Alford RF, Leaver-Fay A, Jeliazkov JR, O’Meara MJ, DiMaio FP, Park H, et al The Rosetta All-Atom Energy Function for Macromolecular Modeling and Design. J Chem Theory Comput. 2017 6 13;13(6):3031–48. 10.1021/acs.jctc.7b00125 28430426 PMC5717763

[28] Park H, Bradley P, Greisen P, Liu Y, Mulligan VK, Kim DE, et al Simultaneous Optimization of Biomolecular Energy Functions on Features from Small Molecules and Macromolecules. J Chem Theory Comput. 2016 12 13;12(12):6201–12. 10.1021/acs.jctc.6b00819 27766851 PMC5515585

[29] Thyme SB, Baker D, Bradley P. Improved modeling of side-chain-base interactions and plasticity in protein-dna interface design. J Mol Biol. 2012 6 8;419(3–4):255–74. 10.1016/j.jmb.2012.03.005 22426128 PMC3566986

[30] Lewell XQ, Judd DB, Watson SP, Hann MM. RECAP—Retrosynthetic Combinatorial Analysis Procedure: A powerful new technique for identifying privileged molecular fragments with useful applications in combinatorial chemistry. J Chem Inf Comput Sci. 1998;38(3):511–22. 10.1021/ci970429i 9611787

[31] Degen J, Wegscheid-Gerlach C, Zaliani A, Rarey M. On the art of compiling and using “drug-like” chemical fragment spaces. ChemMedChem. 2008 10 20;3(10):1503–7. 10.1002/cmdc.200800178 18792903

[32] Kortemme T, Morozov A V, Baker D. An orientation-dependent hydrogen bonding potential improves prediction of specificity and structure for proteins and protein-protein complexes. J Mol Biol. 2003 2 28;326(4):1239–59. 10.1016/s0022-2836(03)00021-4 12589766

[33] Lazaridis T, Karplus M. Effective energy function for proteins in solution. Proteins Struct Funct Bioinforma. 1999 5 1;35(2):133–52. 10.1002/(sici)1097-0134(19990501)35:2<133::aid-prot1>3.0.co;2-n 10223287

[34] DeLano WL. Unraveling hot spots in binding interfaces: Progress and challenges. Curr Opin Struct Biol. 2002 2 1;12(1):14–20. 10.1016/s0959-440x(02)00283-x 11839484

[35] Hawkins PCD, Skillman AG, Warren GL, Ellingson BA, Stahl MT. Conformer generation with OMEGA: Algorithm and validation using high quality structures from the protein databank and cambridge structural database. J Chem Inf Model. 2010 4 26;50(4):572–84. 10.1021/ci100031x 20235588 PMC2859685

[36] RDKit: Open-source cheminformatics.

[37] Allison B, Combs S, DeLuca S, Lemmon G, Mizoue L, Meiler J. Computational design of protein-small molecule interfaces. J Struct Biol. 2014 2 1;185(2):193–202. 10.1016/j.jsb.2013.08.003 23962892 PMC3946393

[38] Deluca S, Dorr B, Meiler J. Design of native-like proteins through an exposure-dependent environment potential. Biochemistry. 2011 10 11;50(40):8521–8. 10.1021/bi200664b 21905701 PMC3752783

[39] O’Meara MJ, Leaver-Fay A, Tyka MD, Stein A, Houlihan K, Dimaio F, et al Combined covalent-electrostatic model of hydrogen bonding improves structure prediction with Rosetta. J Chem Theory Comput. 2015 2 10;11(2):609–22. 10.1021/ct500864r 25866491 PMC4390092

[40] Hu L, Benson ML, Smith RD, Lerner MG, Carlson HA. Binding MOAD (Mother of All Databases). Proteins Struct Funct Genet. 2005 8 15;60(3):333–40. 10.1002/prot.20512 15971202

[41] Lawrence MC, Colman PM. Shape complementarity at protein/protein interfaces. J Mol Biol. 1993 12 20;234(4):946–50. 10.1006/jmbi.1993.1648 8263940

[42] Sheffler W, Baker D. RosettaHoles: Rapid assessment of protein core packing for structure prediction, refinement, design, and validation. Protein Sci. 2009 1 1;18(1):229–39. 10.1002/pro.8 19177366 PMC2708028

[43] Ollikainen N, de Jong RM, Kortemme T. Coupling Protein Side-Chain and Backbone Flexibility Improves the Re-design of Protein-Ligand Specificity. PLoS Comput Biol. 2015;11(9). 10.1371/journal.pcbi.1004335 PMC4580623 26397464

[44] Ollikainen N, Smith CA, Fraser JS, Kortemme T. Flexible backbone sampling methods to model and design protein alternative conformations. Methods Enzymol. 2013 1 1;523:61–85. 10.1016/B978-0-12-394292-0.00004-7 23422426 PMC3750959

[45] Gainza P, Roberts KE, Georgiev I, Lilien RH, Keedy DA, Chen CY, et al Osprey: Protein design with ensembles, flexibility, and provable algorithms. Methods Enzymol. 2013 1 1;523:87–107. 10.1016/B978-0-12-394292-0.00005-9 23422427 PMC3692370

[46] Malisi C, Schumann M, Toussaint NC, Kageyama J, Kohlbacher O, Höcker B. Binding Pocket Optimization by Computational Protein Design. PLoS One. 2012 12 27;7(12). 10.1371/journal.pone.0052505 PMC3531388 23300688

[47] Hallen MA, Keedy DA, Donald BR. Dead-end elimination with perturbations (DEEPer): A provable protein design algorithm with continuous sidechain and backbone flexibility. Proteins Struct Funct Bioinforma. 2013 1 1;81(1):18–39. 10.1002/prot.24150 PMC3491125 22821798

[48] Ladbury JE. Just add water! The effect of water on the specificity of protein- ligand binding sites and its potential application to drug design. Chem Biol. 1996 12 1;3(12):973–80. 10.1016/s1074-5521(96)90164-7 9000013

[49] Breiten B, Lockett MR, Sherman W, Fujita S, Al-Sayah M, Lange H, et al Water networks contribute to enthalpy/entropy compensation in protein-ligand binding. J Am Chem Soc. 2013 10 16;135(41):15579–84. 10.1021/ja4075776 24044696

[50] Hosseinzadeh P, Bhardwaj G, Mulligan VK, Shortridge MD, Craven TW, Pardo-Avila F, et al Comprehensive computational design of ordered peptide macrocycles. Science (80-). 2017 12 15;358(6369):1461–6. 10.1126/science.aap7577 29242347 PMC5860875

[51] Jacobs TM, Williams B, Williams T, Xu X, Eletsky A, Federizon JF, et al Design of structurally distinct proteins using strategies inspired by evolution. Science (80-). 2016 5 6;352(6286):687–90. 10.1126/science.aad8036 27151863 PMC4934125

[52] Guffy SL, Teets FD, Langlois MI, Kuhlman B. Protocols for Requirement-Driven Protein Design in the Rosetta Modeling Program. J Chem Inf Model. 2018 5 29;58(5):895–901. 10.1021/acs.jcim.8b00060 29659276 PMC5975180

[53] Kundert K, Kortemme T. Computational design of structured loops for new protein functions Vol. 400, Biological Chemistry. De Gruyter; 2019 p. 275–88. 10.1515/hsz-2018-0348 30676995 PMC6530579

[54] Chaudhury S, Lyskov S, Gray JJ. PyRosetta: a script-based interface for implementing molecular modeling algorithms using Rosetta. Bioinformatics. 2010 3 1;26(5):689–91. 10.1093/bioinformatics/btq007 20061306 PMC2828115

[55] Bakan A, Meireles LM, Bahar I. ProDy: Protein Dynamics Inferred from Theory and Experiments. Bioinformatics. 2011 6 1;27(11):1575–7. 10.1093/bioinformatics/btr168 21471012 PMC3102222

[56] Hanwell MD, Curtis DE, Lonie DC, Vandermeerschd T, Zurek E, Hutchison GR. Avogadro: An advanced semantic chemical editor, visualization, and analysis platform. J Cheminform. 2012 8 13;4(8):17. 22889332 10.1186/1758-2946-4-17 PMC3542060

[57] Sunghwan Kim, Jie Chen, Tiejun Cheng, Asta Gindulyte, Jia He, Siqian He, Qingliang Li, Benjamin A Shoemaker, Paul A Thiessen, Bo Yu, Leonid Zaslavsky, Jian Zhang EEB. PubChem 2019 update: improved access to chemical data. Nucleic Acids Res. 2019 1 8;47(D1):D1102–9. 10.1093/nar/gky1033 30371825 PMC6324075

[58] Feng Z, Chen L, Maddula H, Akcan O, Oughtred R, Berman HM, et al Ligand Depot: A data warehouse for ligands bound to macromolecules. Bioinformatics. 2004 9 1;20(13):2153–5. 10.1093/bioinformatics/bth214 15059838

[59] Yona G, Levitt M. Within the twilight zone: A sensitive profile-profile comparison tool based on information theory. J Mol Biol. 2002 2 1;315(5):1257–75. 10.1006/jmbi.2001.5293 11827492

[60] Lucas JE. New Computational Protein Design Methods for De Novo Small Molecule Binding Sites. PhD Thesis, University of California, San Francisco and University of California, Berkeley; 2020. Available from: https://escholarship.org/uc/item/5p41p5vh 10.1371/journal.pcbi.1008178 PMC7575090 33017412

