# Supplementary material for: New computational protein design methods for de novo small molecule binding sites
Source: PLoS Comput Biol. 2020 Oct 5;16(10):e1008178. doi: 10.1371/journal.pcbi.1008178 (PMC7575090; doi:10.1371/journal.pcbi.1008178)

Binding Site Recovery Benchmark Sequence Logos

Contents

1 5J5I **2**

2 1SRI **3**

3 2XN3 **4**

4 2QRY **5**

5 5HZ8 **6**

6 1LNM **7**

7 4QAC **8**

8 5HZ6 **9**

9 5EDB **10**

10 1LKE **11**

11 5T52 **12**

12 5J5G **13**

13 3CZ1 **14**

14 3OKI **15**

15 4AFG **16**

16 4AFH **17**

17 6M9B **18**

18 4B5D **19**

19 1N0S **20**

20 2IZL **21**

21 5URA **22**

22 1TOU **23**

1 1N0S

(a) special_rot bonus: 0.0

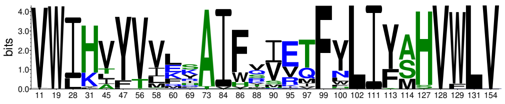

(b) special_rot bonus: -0.5

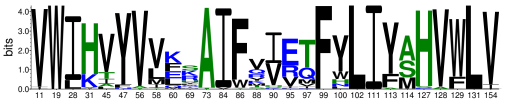

(c) special_rot bonus: -1.0

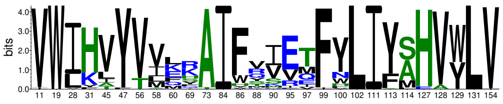

(d) special_rot bonus: -1.5

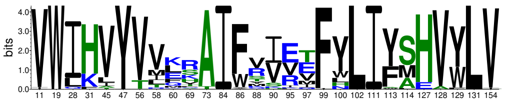

(e) special_rot bonus: -2.0

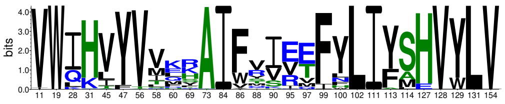

(f) special_rot bonus: -2.5

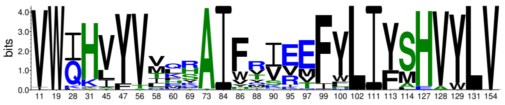

(g) special_rot bonus: -3.0

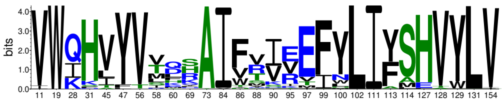

(h) special_rot bonus: -3.5

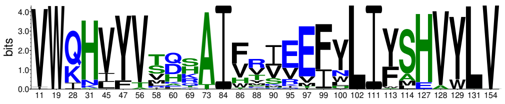

(i) special_rot bonus: -4.0

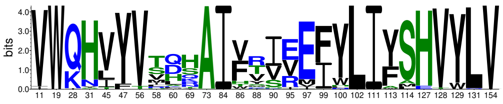

(j) special_rot bonus: -4.5

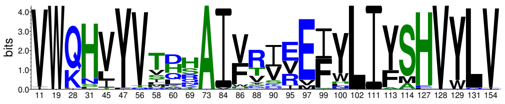

(k) special_rot bonus: -5.0

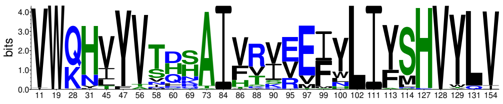

(l) special_rot bonus: -6.0

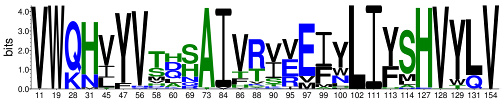

(m) special_rot bonus: -8.0

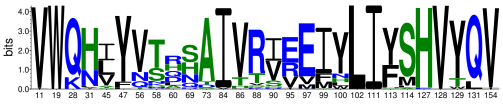

(n) special_rot bonus: -10.0

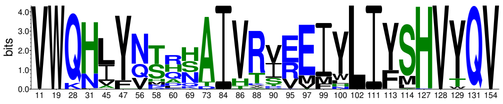

(o) special_rot bonus: Control

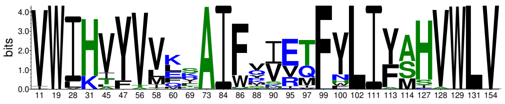

(p) special_rot bonus: Native

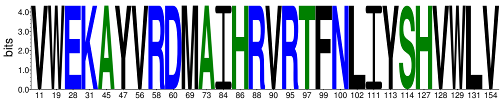

2 5URA

(a) special_rot bonus: 0.0

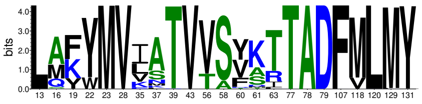

(b) special_rot bonus: -0.5

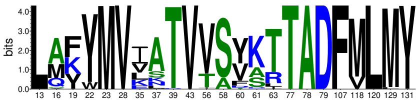

(c) special_rot bonus: -1.0

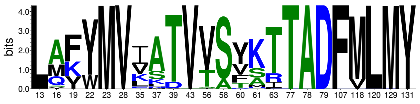

(d) special_rot bonus: -1.5

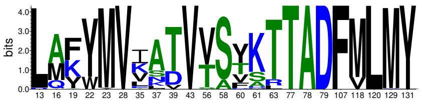

(e) special_rot bonus: -2.0

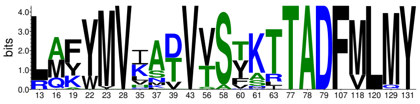

(f) special_rot bonus: -2.5

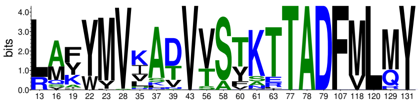

(g) special_rot bonus: -3.0

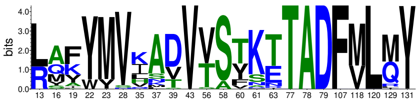

(h) special_rot bonus: -3.5

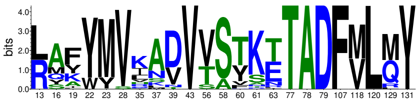

(i) special_rot bonus: -4.0

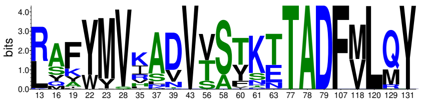

(j) special_rot bonus: -4.5

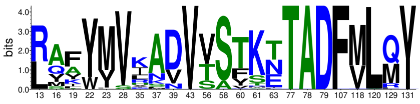

(k) special_rot bonus: -5.0

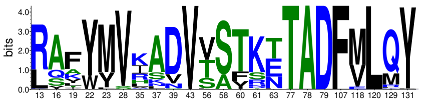

(l) special_rot bonus: -6.0

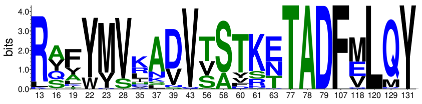

(m) special_rot bonus: -8.0

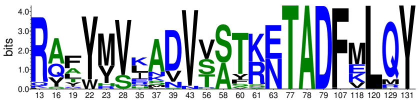

(n) special_rot bonus: -10.0

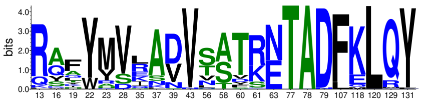

(o) special_rot bonus: Control

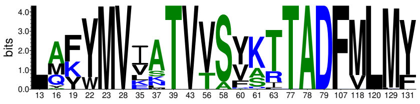

(p) special_rot bonus: Native

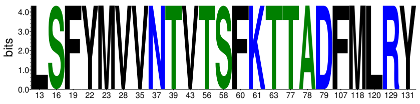

3 5J5I

(a) special_rot bonus: 0.0

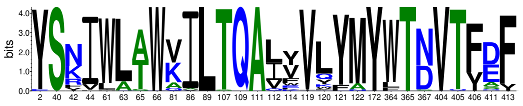

(b) special_rot bonus: -0.5

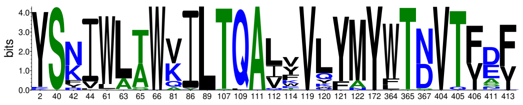

(c) special_rot bonus: -1.0

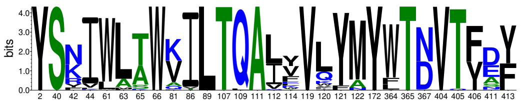

(d) special_rot bonus: -1.5

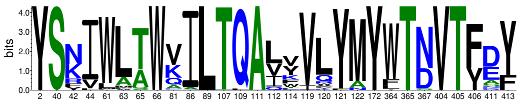

(e) special_rot bonus: -2.0

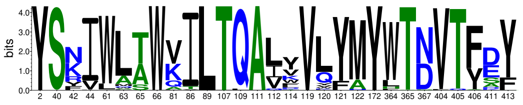

(f) special_rot bonus: -2.5

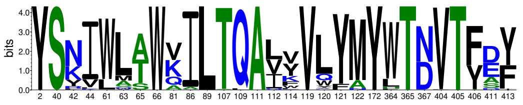

(g) special_rot bonus: -3.0

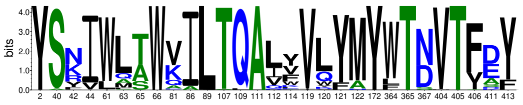

(h) special_rot bonus: -3.5

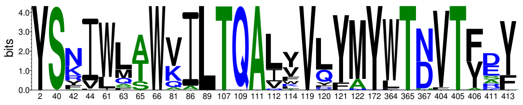

(i) special_rot bonus: -4.0

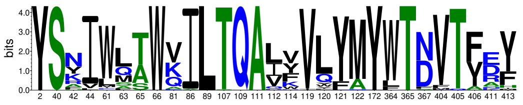

(j) special_rot bonus: -4.5

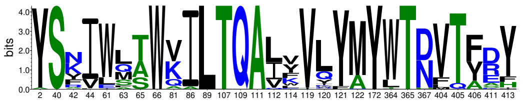

(k) special_rot bonus: -5.0

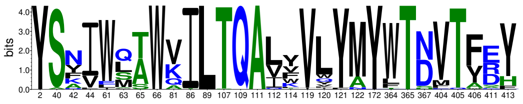

(l) special_rot bonus: -6.0

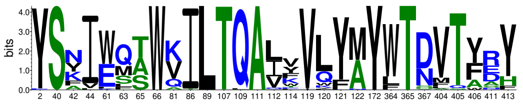

(m) special_rot bonus: -8.0

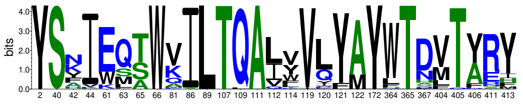

(n) special_rot bonus: -10.0

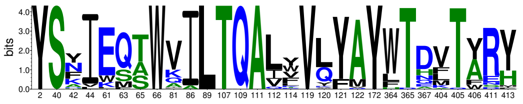

(o) special_rot bonus: Control

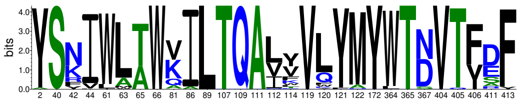

(p) special_rot bonus: Native

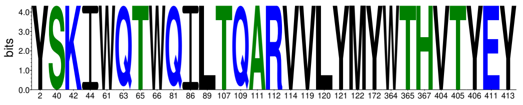

4 4B5D

(a) special_rot bonus: 0.0

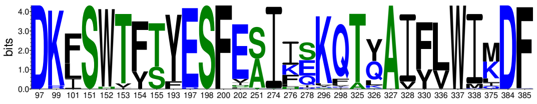

(b) special_rot bonus: -0.5

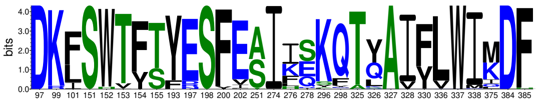

(c) special_rot bonus: -1.0

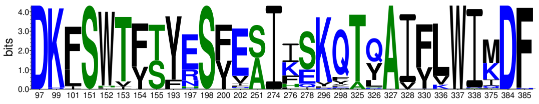

(d) special_rot bonus: -1.5

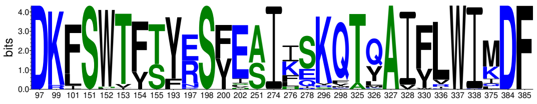

(e) special_rot bonus: -2.0

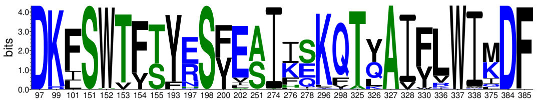

(f) special_rot bonus: -2.5

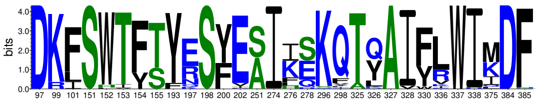

(g) special_rot bonus: -3.0

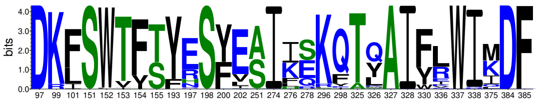

(h) special_rot bonus: -3.5

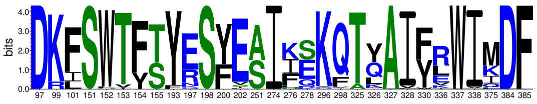

(i) special_rot bonus: -4.0

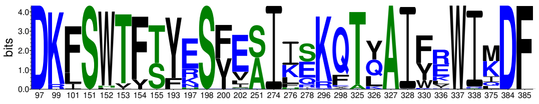

(j) special_rot bonus: -4.5

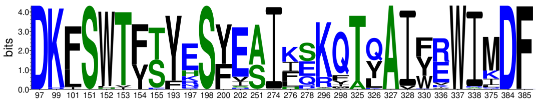

(k) special_rot bonus: -5.0

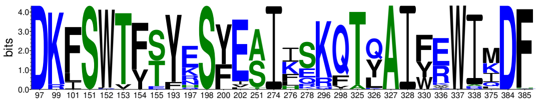

(l) special_rot bonus: -6.0

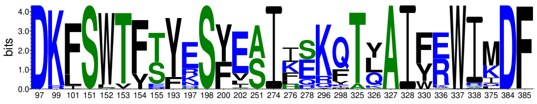

(m) special_rot bonus: -8.0

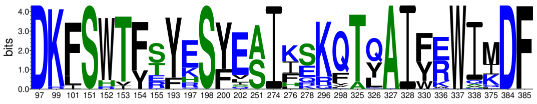

(n) special_rot bonus: -10.0

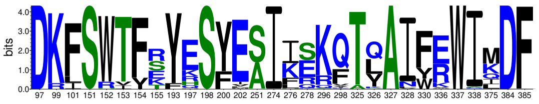

(o) special_rot bonus: Control

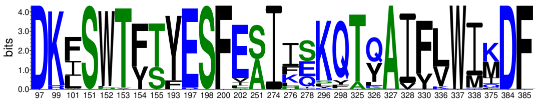

(p) special_rot bonus: Native

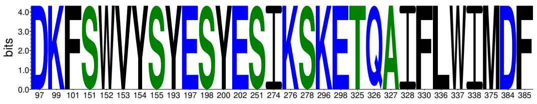

5 5J5G

(a) special_rot bonus: 0.0

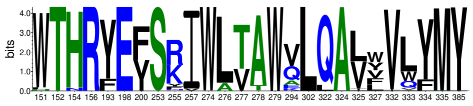

(b) special_rot bonus: -0.5

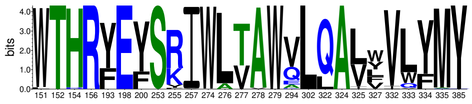

(c) special_rot bonus: -1.0

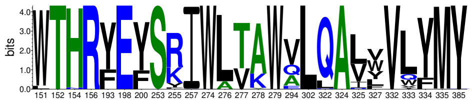

(d) special_rot bonus: -1.5

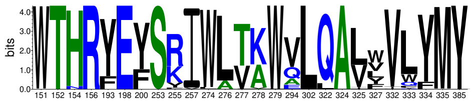

(e) special_rot bonus: -2.0

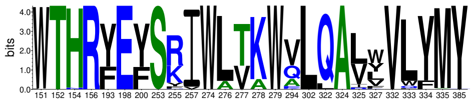

(f) special_rot bonus: -2.5

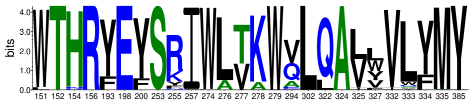

(g) special_rot bonus: -3.0

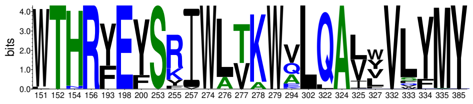

(h) special_rot bonus: -3.5

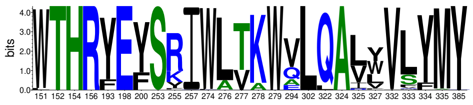

(i) special_rot bonus: -4.0

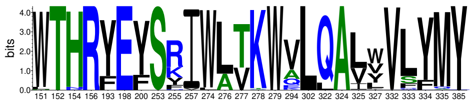

(j) special_rot bonus: -4.5

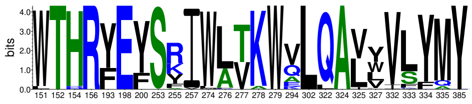

(k) special_rot bonus: -5.0

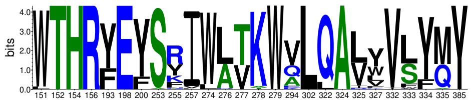

(l) special_rot bonus: -6.0

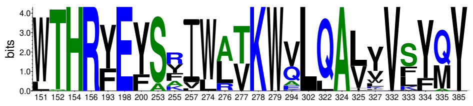

(m) special_rot bonus: -8.0

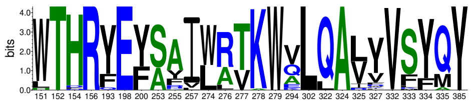

(n) special_rot bonus: -10.0

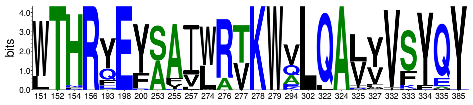

(o) special_rot bonus: Control

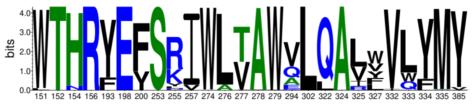

(p) special_rot bonus: Native

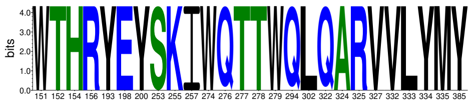

6 4QAC

(a) special_rot bonus: 0.0

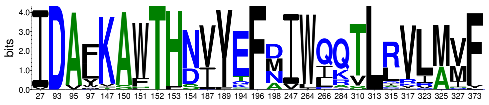

(b) special_rot bonus: -0.5

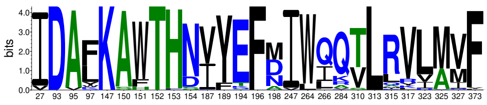

(c) special_rot bonus: -1.0

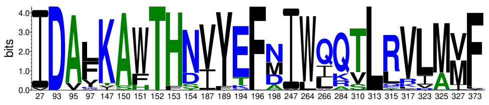

(d) special_rot bonus: -1.5

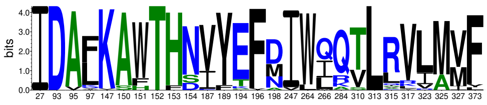

(e) special_rot bonus: -2.0

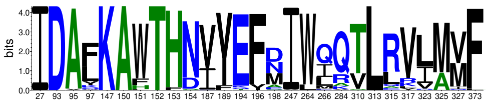

(f) special_rot bonus: -2.5

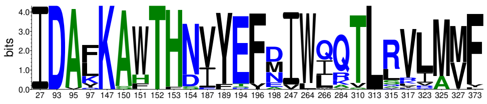

(g) special_rot bonus: -3.0

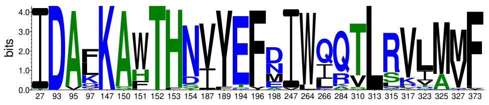

(h) special_rot bonus: -3.5

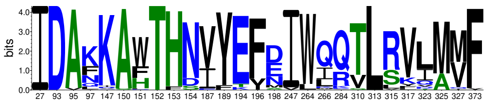

(i) special_rot bonus: -4.0

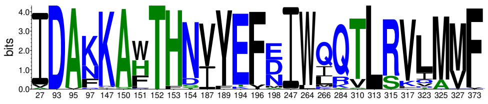

(j) special_rot bonus: -4.5

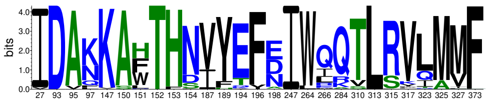

(k) special_rot bonus: -5.0

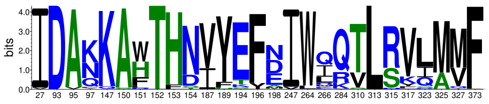

(l) special_rot bonus: -6.0

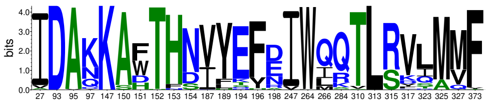

(m) special_rot bonus: -8.0

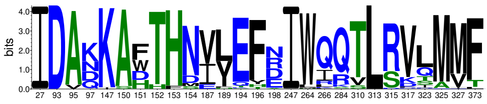

(n) special_rot bonus: -10.0

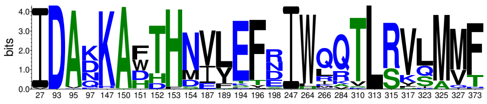

(o) special_rot bonus: Control

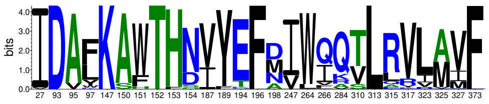

(p) special_rot bonus: Native

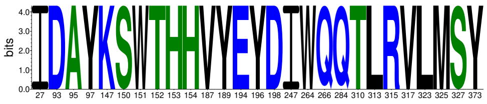

7 1LNM

(a) special_rot bonus: 0.0

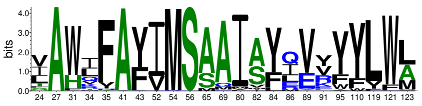

(b) special_rot bonus: -0.5

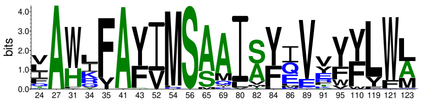

(c) special_rot bonus: -1.0

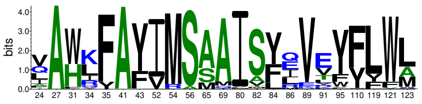

(d) special_rot bonus: -1.5

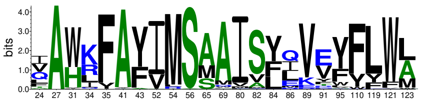

(e) special_rot bonus: -2.0

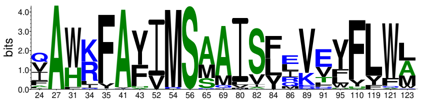

(f) special_rot bonus: -2.5

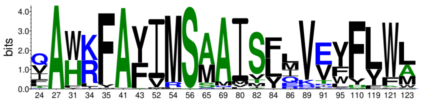

(g) special_rot bonus: -3.0

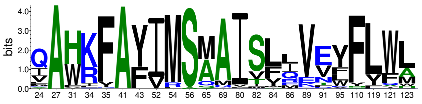

(h) special_rot bonus: -3.5

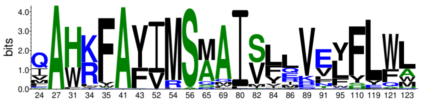

(i) special_rot bonus: -4.0

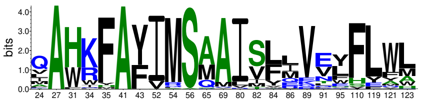

(j) special_rot bonus: -4.5

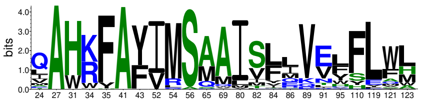

(k) special_rot bonus: -5.0

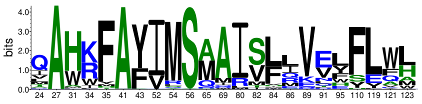

(l) special_rot bonus: -6.0

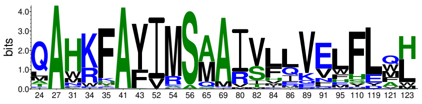

(m) special_rot bonus: -8.0

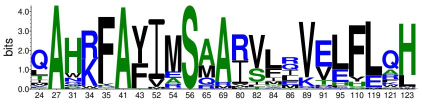

(n) special_rot bonus: -10.0

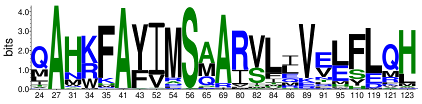

(o) special_rot bonus: Control

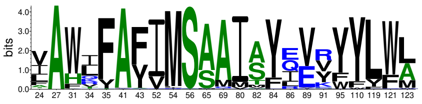

(p) special_rot bonus: Native

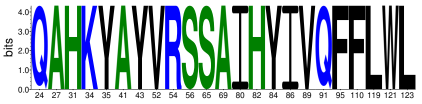

8 3CZ1

(a) special_rot bonus: 0.0

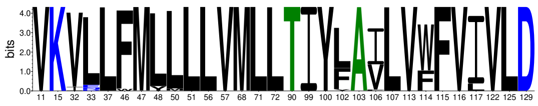

(b) special_rot bonus: -0.5

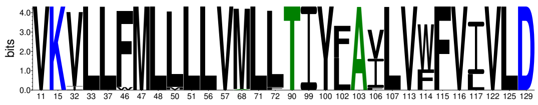

(c) special_rot bonus: -1.0

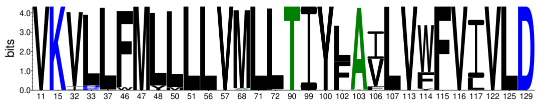

(d) special_rot bonus: -1.5

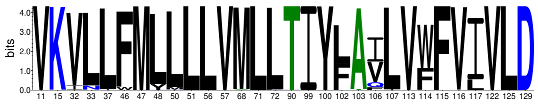

(e) special_rot bonus: -2.0

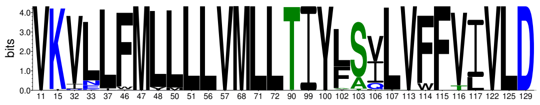

(f) special_rot bonus: -2.5

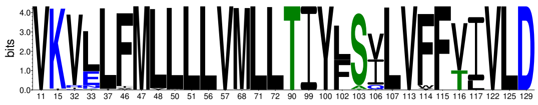

(g) special_rot bonus: -3.0

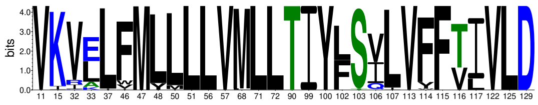

(h) special_rot bonus: -3.5

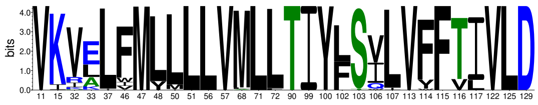

(i) special_rot bonus: -4.0

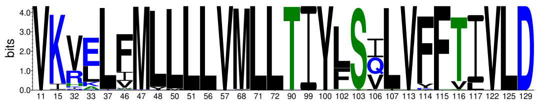

(j) special_rot bonus: -4.5

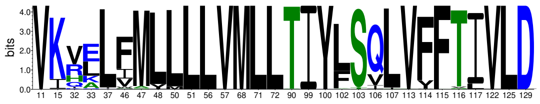

(k) special_rot bonus: -5.0

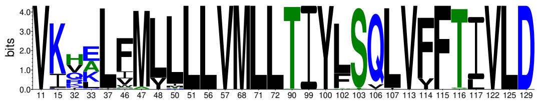

(l) special_rot bonus: -6.0

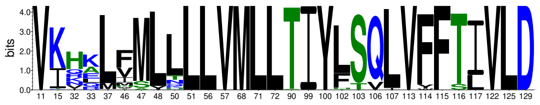

(m) special_rot bonus: -8.0

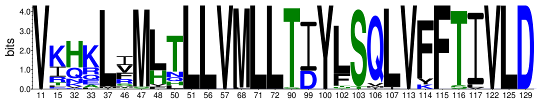

(n) special_rot bonus: -10.0

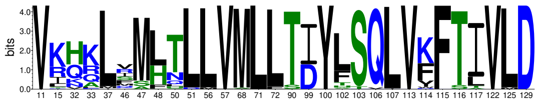

(o) special_rot bonus: Control

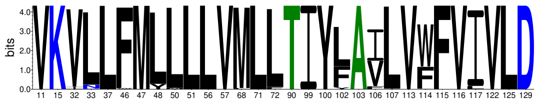

(p) special_rot bonus: Native

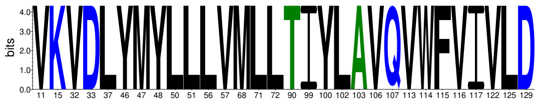

9 6M9B

(a) special_rot bonus: 0.0

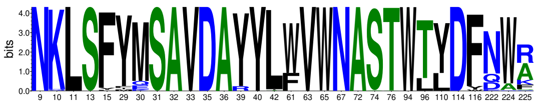

(b) special_rot bonus: -0.5

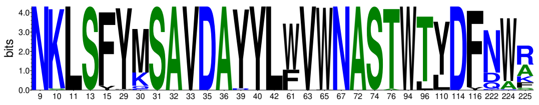

(c) special_rot bonus: -1.0

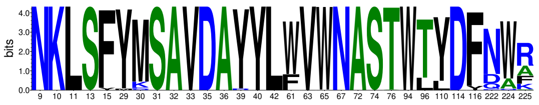

(d) special_rot bonus: -1.5

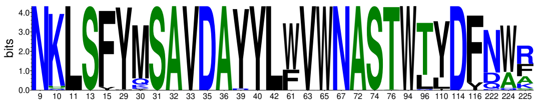

(e) special_rot bonus: -2.0

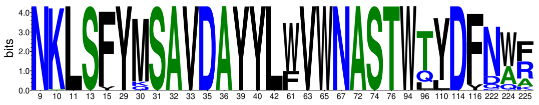

(f) special_rot bonus: -2.5

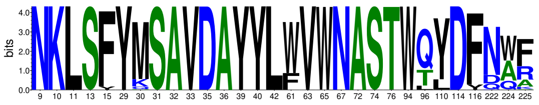

(g) special_rot bonus: -3.0

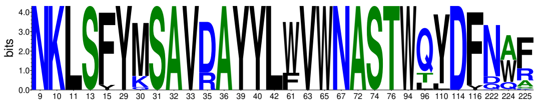

(h) special_rot bonus: -3.5

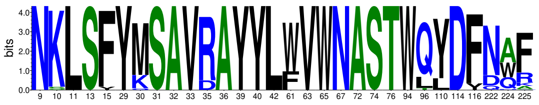

(i) special_rot bonus: -4.0

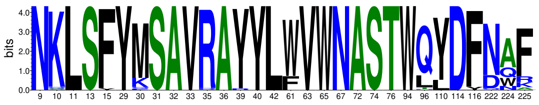

(j) special_rot bonus: -4.5

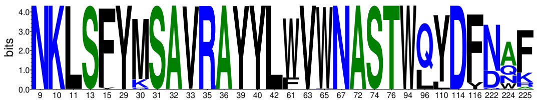

(k) special_rot bonus: -5.0

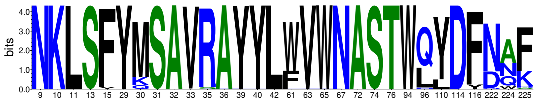

(l) special_rot bonus: -6.0

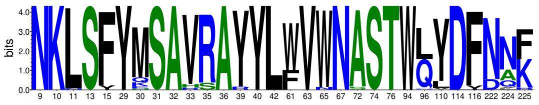

(m) special_rot bonus: -8.0

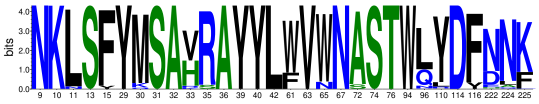

(n) special_rot bonus: -10.0

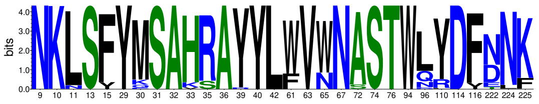

(o) special_rot bonus: Control

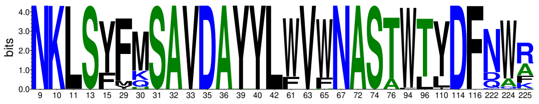

(p) special_rot bonus: Native

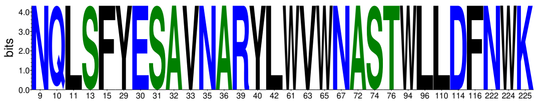

10 5EDB

(a) special_rot bonus: 0.0

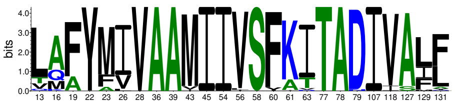

(b) special_rot bonus: -0.5

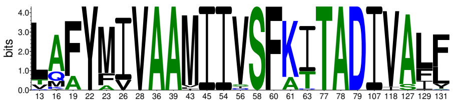

(c) special_rot bonus: -1.0

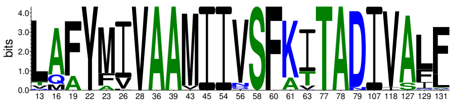

(d) special_rot bonus: -1.5

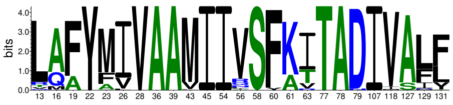

(e) special_rot bonus: -2.0

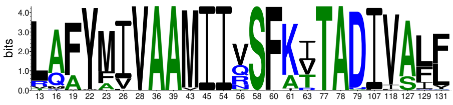

(f) special_rot bonus: -2.5

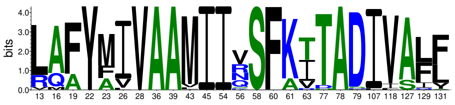

(g) special_rot bonus: -3.0

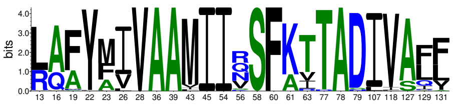

(h) special_rot bonus: -3.5

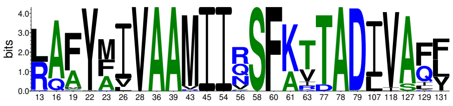

(i) special_rot bonus: -4.0

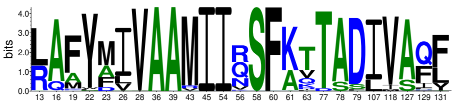

(j) special_rot bonus: -4.5

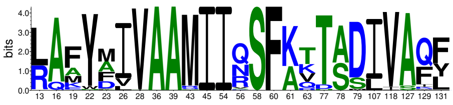

(k) special_rot bonus: -5.0

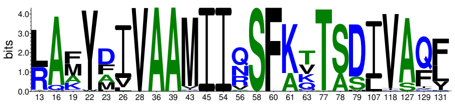

(l) special_rot bonus: -6.0

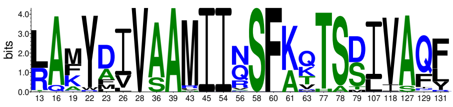

(m) special_rot bonus: -8.0

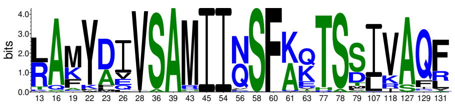

(n) special_rot bonus: -10.0

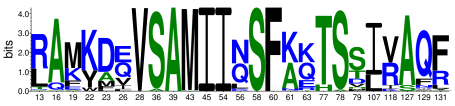

(o) special_rot bonus: Control

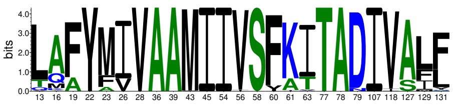

(p) special_rot bonus: Native

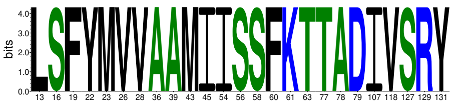

11 1LKE

(a) special_rot bonus: 0.0

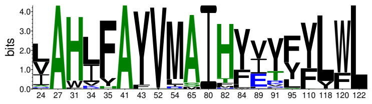

(b) special_rot bonus: -0.5

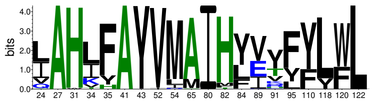

(c) special_rot bonus: -1.0

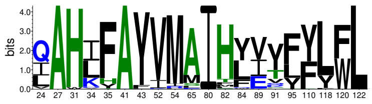

(d) special_rot bonus: -1.5

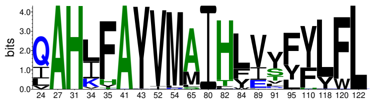

(e) special_rot bonus: -2.0

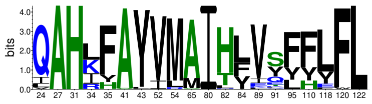

(f) special_rot bonus: -2.5

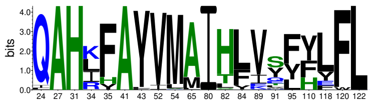

(g) special_rot bonus: -3.0

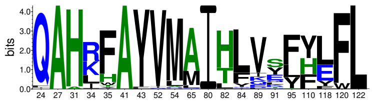

(h) special_rot bonus: -3.5

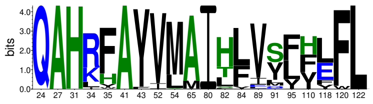

(i) special_rot bonus: -4.0

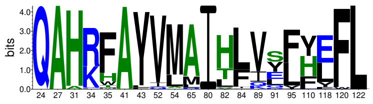

(j) special_rot bonus: -4.5

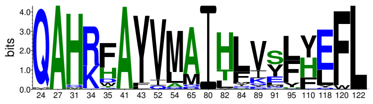

(k) special_rot bonus: -5.0

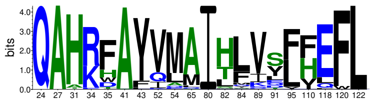

(l) special_rot bonus: -6.0

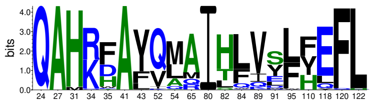

(m) special_rot bonus: -8.0

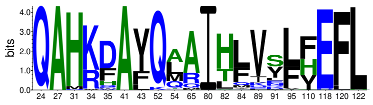

(n) special_rot bonus: -10.0

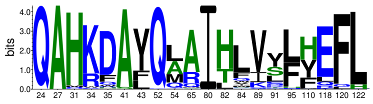

(o) special_rot bonus: Control

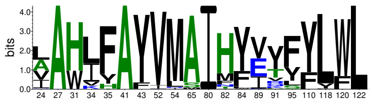

(p) special_rot bonus: Native

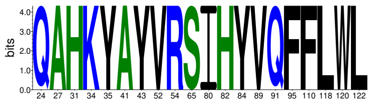

12 2XN3

(a) special_rot bonus: 0.0

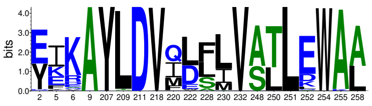

(b) special_rot bonus: -0.5

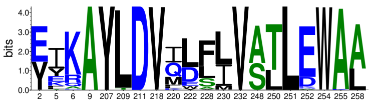

(c) special_rot bonus: -1.0

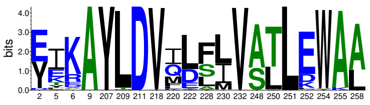

(d) special_rot bonus: -1.5

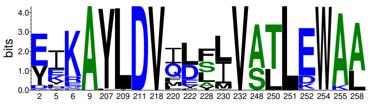

(e) special_rot bonus: -2.0

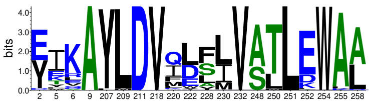

(f) special_rot bonus: -2.5

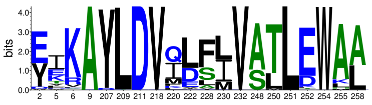

(g) special_rot bonus: -3.0

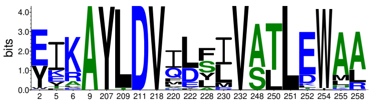

(h) special_rot bonus: -3.5

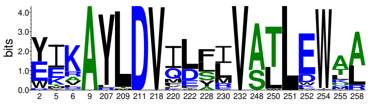

(i) special_rot bonus: -4.0

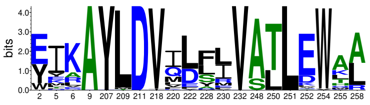

(j) special_rot bonus: -4.5

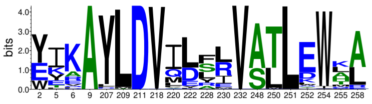

(k) special_rot bonus: -5.0

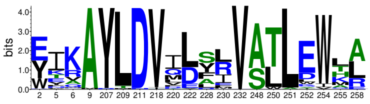

(l) special_rot bonus: -6.0

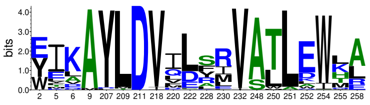

(m) special_rot bonus: -8.0

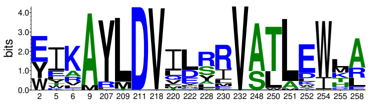

(n) special_rot bonus: -10.0

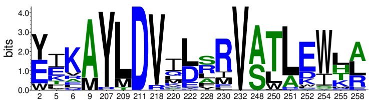

(o) special_rot bonus: Control

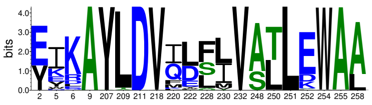

(p) special_rot bonus: Native

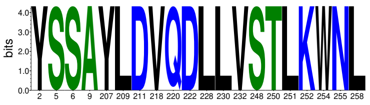

13 2QRY

(a) special_rot bonus: 0.0

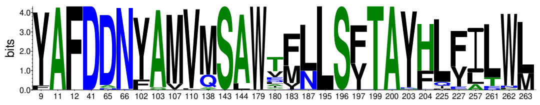

(b) special_rot bonus: -0.5

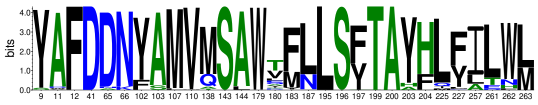

(c) special_rot bonus: -1.0

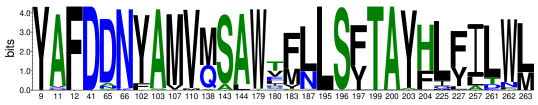

(d) special_rot bonus: -1.5

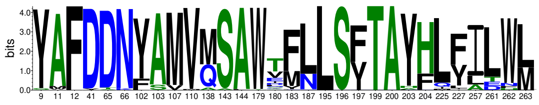

(e) special_rot bonus: -2.0

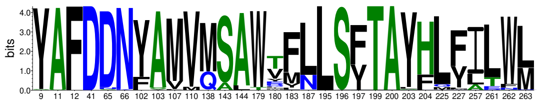

(f) special_rot bonus: -2.5

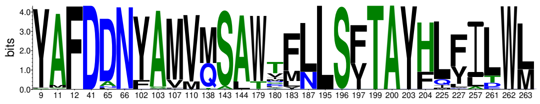

(g) special_rot bonus: -3.0

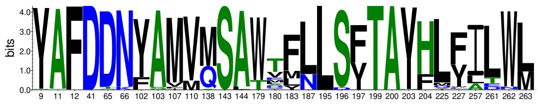

(h) special_rot bonus: -3.5

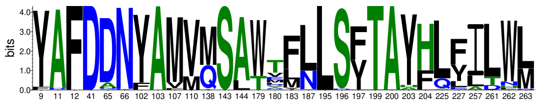

(i) special_rot bonus: -4.0

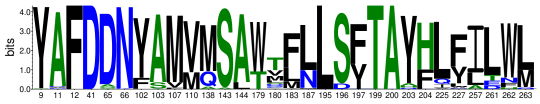

(j) special_rot bonus: -4.5

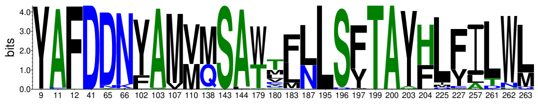

(k) special_rot bonus: -5.0

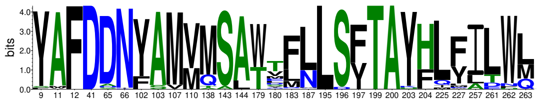

(l) special_rot bonus: -6.0

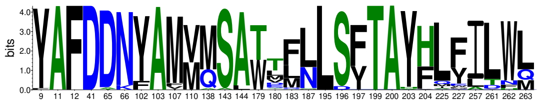

(m) special_rot bonus: -8.0

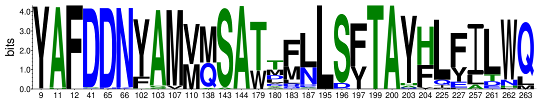

(n) special_rot bonus: -10.0

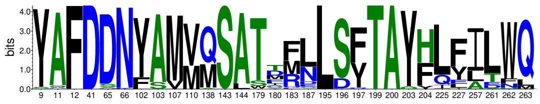

(o) special_rot bonus: Control

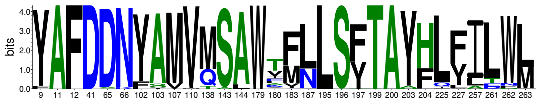

(p) special_rot bonus: Native

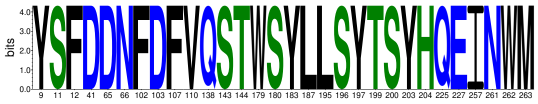

14 3OKI

(a) special_rot bonus: 0.0

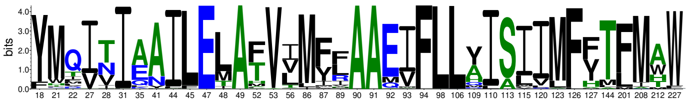

(b) special_rot bonus: -0.5

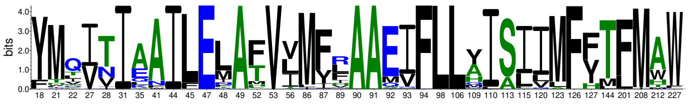

(c) special_rot bonus: -1.0

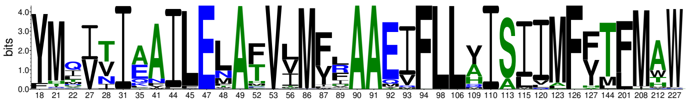

(d) special_rot bonus: -1.5

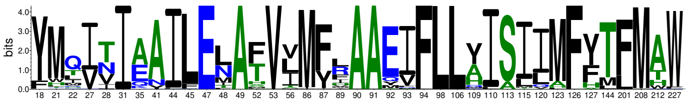

(e) special_rot bonus: -2.0

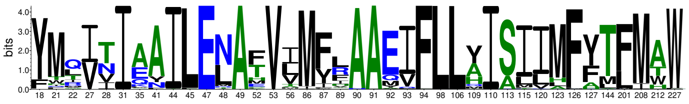

(f) special_rot bonus: -2.5

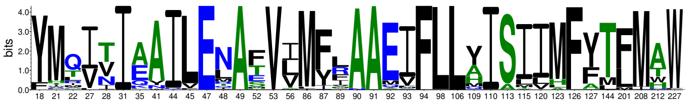

(g) special_rot bonus: -3.0

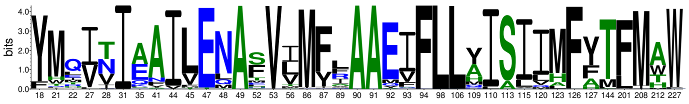

(h) special_rot bonus: -3.5

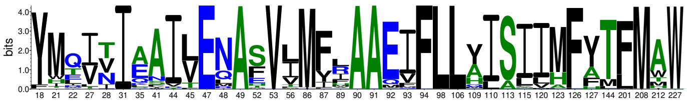

(i) special_rot bonus: -4.0

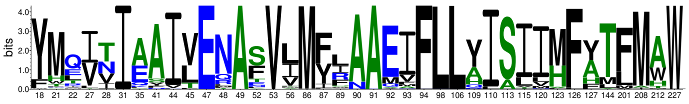

(j) special_rot bonus: -4.5

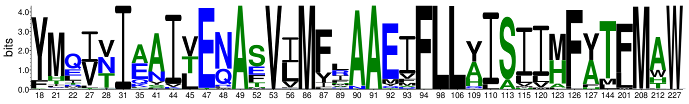

(k) special_rot bonus: -5.0

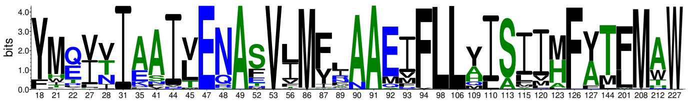

(l) special_rot bonus: -6.0

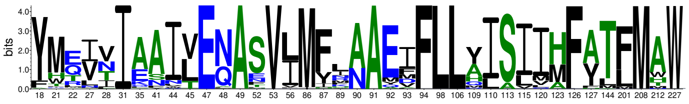

(m) special_rot bonus: -8.0

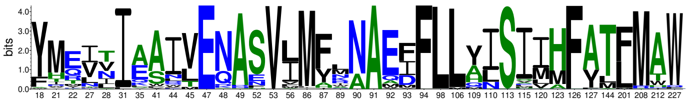

(n) special_rot bonus: -10.0

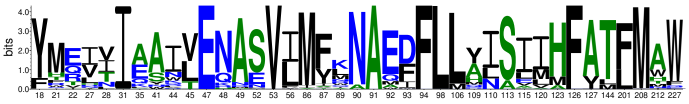

(o) special_rot bonus: Control

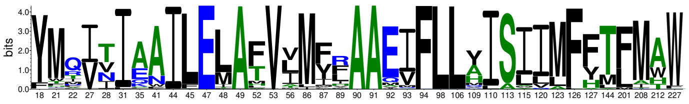

(p) special_rot bonus: Native

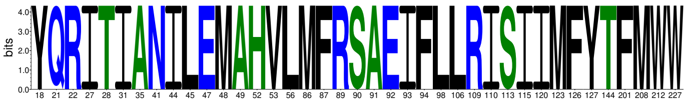

15 5HZ6

(a) special_rot bonus: 0.0

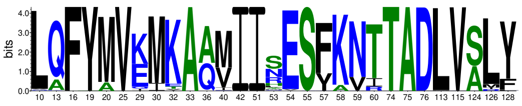

(b) special_rot bonus: -0.5

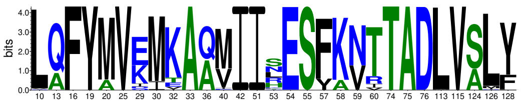

(c) special_rot bonus: -1.0

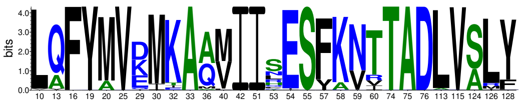

(d) special_rot bonus: -1.5

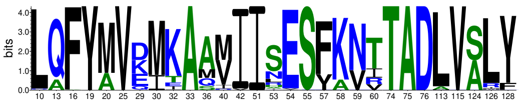

(e) special_rot bonus: -2.0

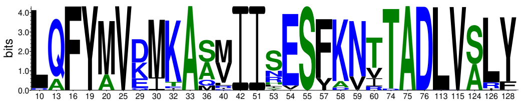

(f) special_rot bonus: -2.5

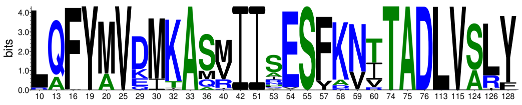

(g) special_rot bonus: -3.0

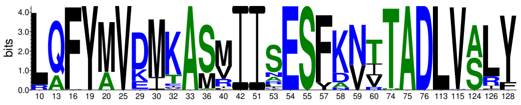

(h) special_rot bonus: -3.5

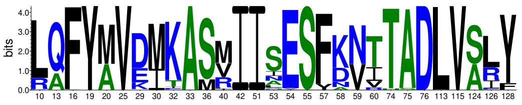

(i) special_rot bonus: -4.0

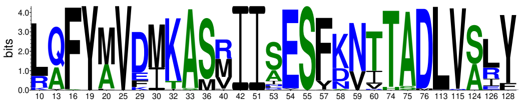

(j) special_rot bonus: -4.5

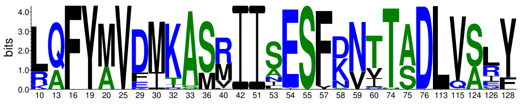

(k) special_rot bonus: -5.0

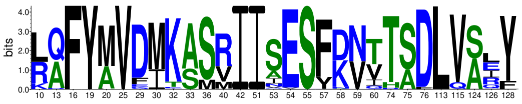

(l) special_rot bonus: -6.0

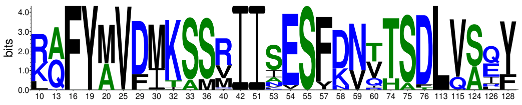

(m) special_rot bonus: -8.0

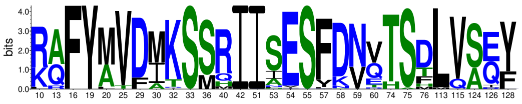

(n) special_rot bonus: -10.0

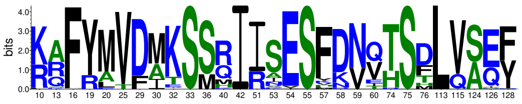

(o) special_rot bonus: Control

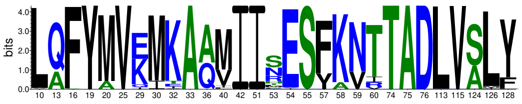

(p) special_rot bonus: Native

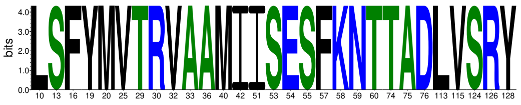

16 1SRI

(a) special_rot bonus: 0.0

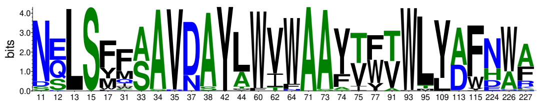

(b) special_rot bonus: -0.5

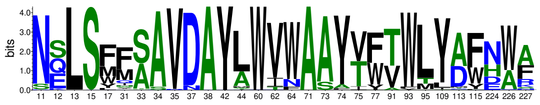

(c) special_rot bonus: -1.0

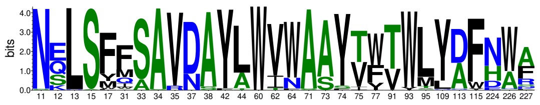

(d) special_rot bonus: -1.5

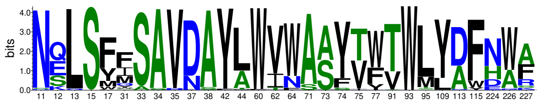

(e) special_rot bonus: -2.0

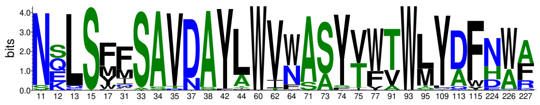

(f) special_rot bonus: -2.5

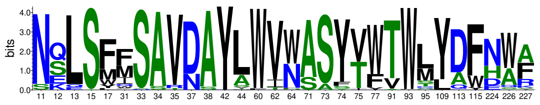

(g) special_rot bonus: -3.0

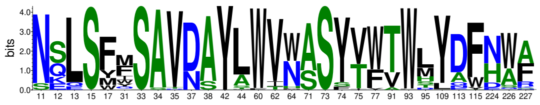

(h) special_rot bonus: -3.5

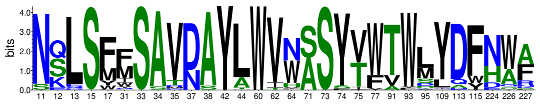

(i) special_rot bonus: -4.0

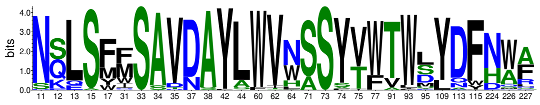

(j) special_rot bonus: -4.5

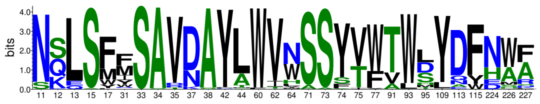

(k) special_rot bonus: -5.0

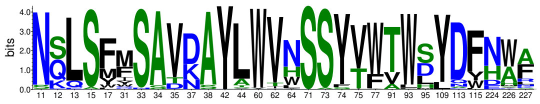

(l) special_rot bonus: -6.0

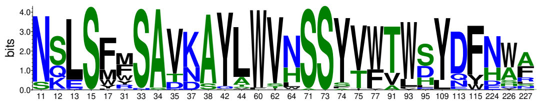

(m) special_rot bonus: -8.0

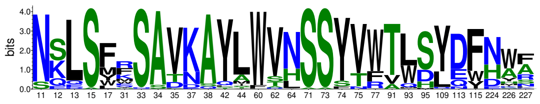

(n) special_rot bonus: -10.0

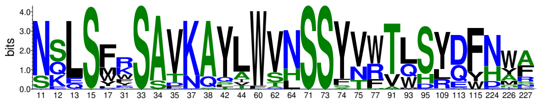

(o) special_rot bonus: Control

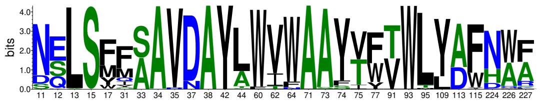

(p) special_rot bonus: Native

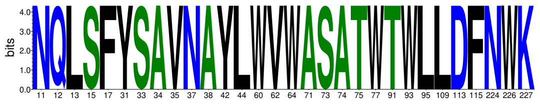

17 5T52

(a) special_rot bonus: 0.0

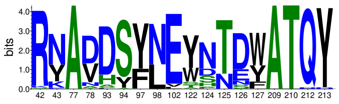

(b) special_rot bonus: -0.5

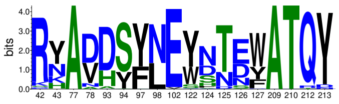

(c) special_rot bonus: -1.0

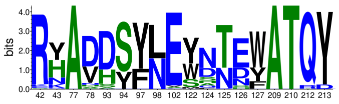

(d) special_rot bonus: -1.5

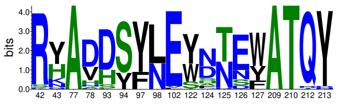

(e) special_rot bonus: -2.0

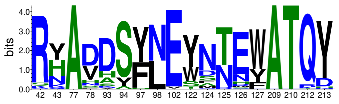

(f) special_rot bonus: -2.5

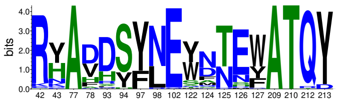

(g) special_rot bonus: -3.0

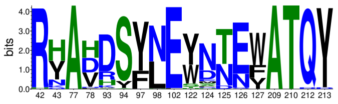

(h) special_rot bonus: -3.5

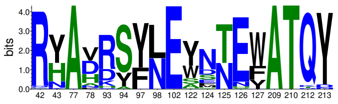

(i) special_rot bonus: -4.0

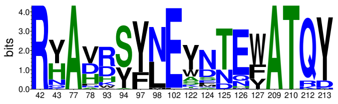

(j) special_rot bonus: -4.5

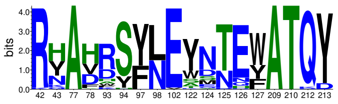

(k) special_rot bonus: -5.0

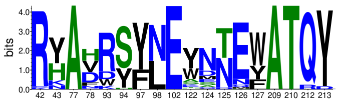

(l) special_rot bonus: -6.0

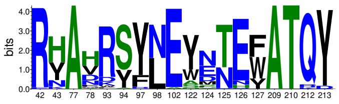

(m) special_rot bonus: -8.0

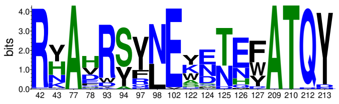

(n) special_rot bonus: -10.0

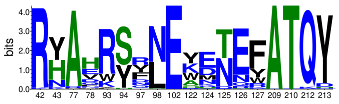

(o) special_rot bonus: Control

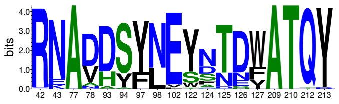

(p) special_rot bonus: Native

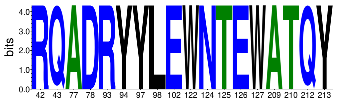

18 5HZ8

(a) special_rot bonus: 0.0

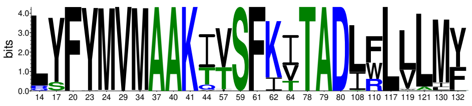

(b) special_rot bonus: -0.5

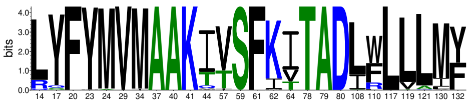

(c) special_rot bonus: -1.0

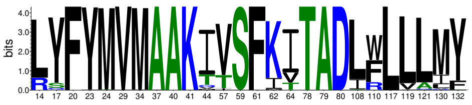

(d) special_rot bonus: -1.5

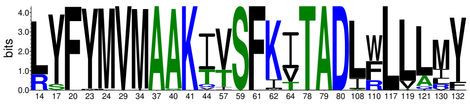

(e) special_rot bonus: -2.0

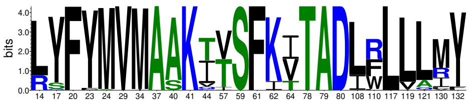

(f) special_rot bonus: -2.5

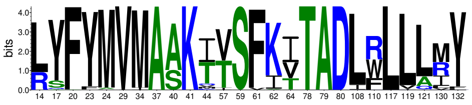

(g) special_rot bonus: -3.0

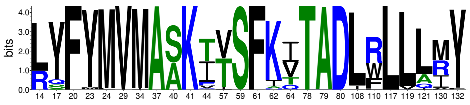

(h) special_rot bonus: -3.5

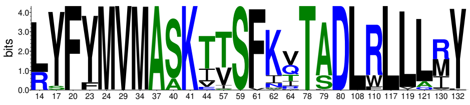

(i) special_rot bonus: -4.0

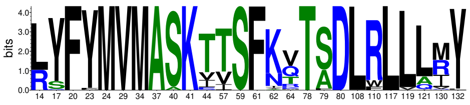

(j) special_rot bonus: -4.5

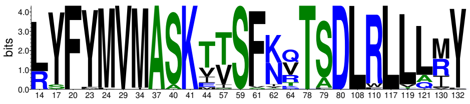

(k) special_rot bonus: -5.0

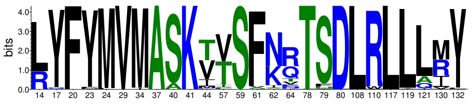

(l) special_rot bonus: -6.0

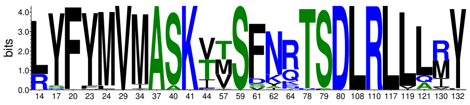

(m) special_rot bonus: -8.0

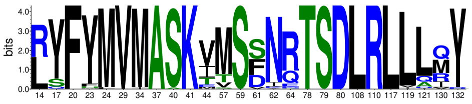

(n) special_rot bonus: -10.0

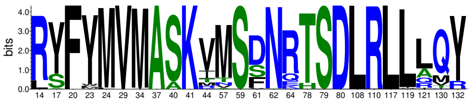

(o) special_rot bonus: Control

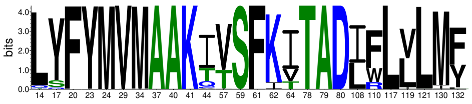

(p) special_rot bonus: Native

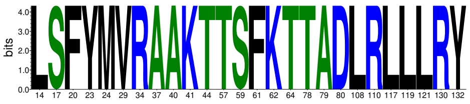

19 1TOU

(a) special_rot bonus: 0.0

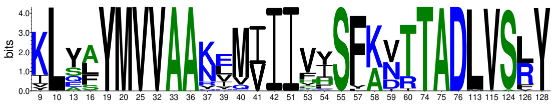

(b) special_rot bonus: -0.5

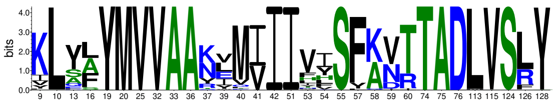

(c) special_rot bonus: -1.0

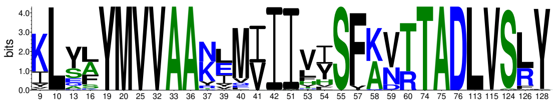

(d) special_rot bonus: -1.5

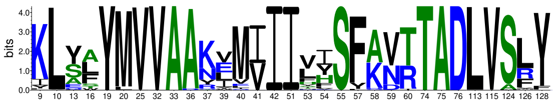

(e) special_rot bonus: -2.0

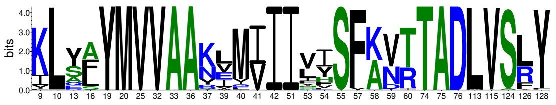

(f) special_rot bonus: -2.5

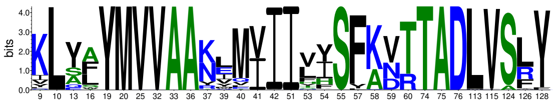

(g) special_rot bonus: -3.0

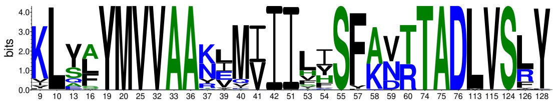

(h) special_rot bonus: -3.5

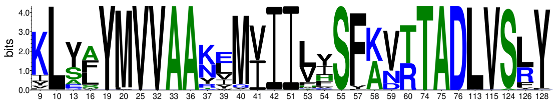

(i) special_rot bonus: -4.0

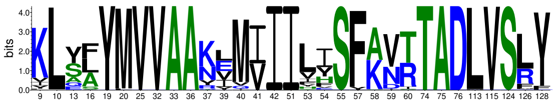

(j) special_rot bonus: -4.5

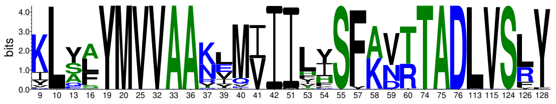

(k) special_rot bonus: -5.0

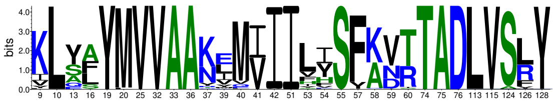

(l) special_rot bonus: -6.0

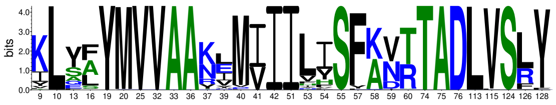

(m) special_rot bonus: -8.0

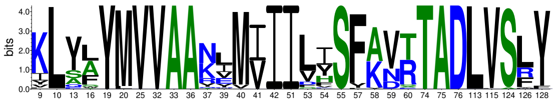

(n) special_rot bonus: -10.0

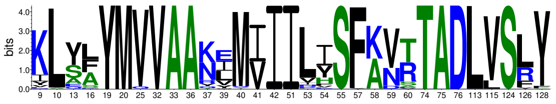

(o) special_rot bonus: Control

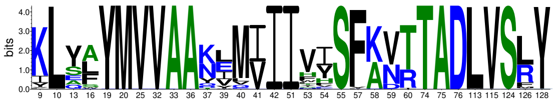

(p) special_rot bonus: Native

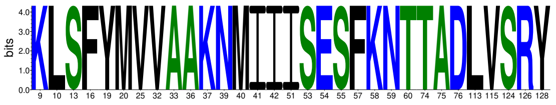

20 2IZL

(a) special_rot bonus: 0.0

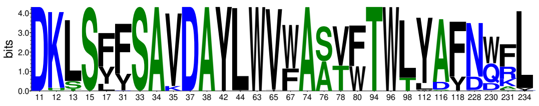

(b) special_rot bonus: -0.5

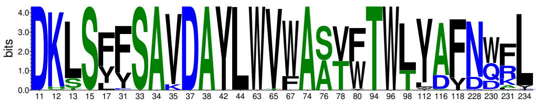

(c) special_rot bonus: -1.0

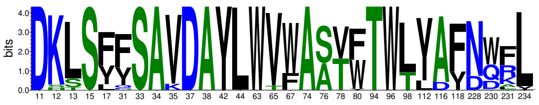

(d) special_rot bonus: -1.5

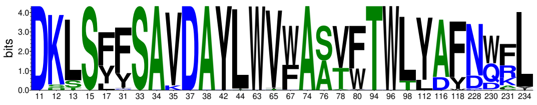

(e) special_rot bonus: -2.0

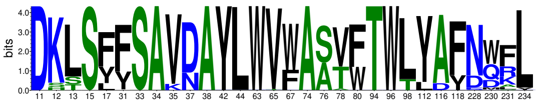

(f) special_rot bonus: -2.5

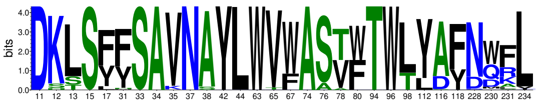

(g) special_rot bonus: -3.0

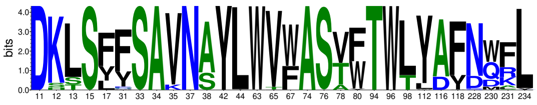

(h) special_rot bonus: -3.5

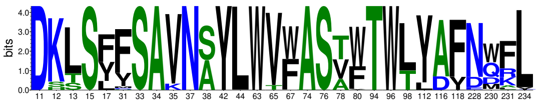

(i) special_rot bonus: -4.0

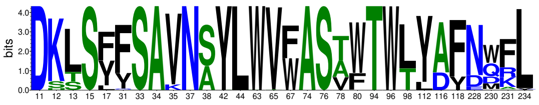

(j) special_rot bonus: -4.5

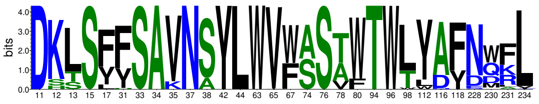

(k) special_rot bonus: -5.0

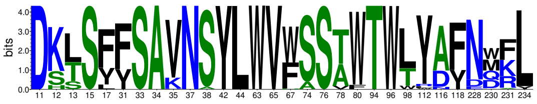

(l) special_rot bonus: -6.0

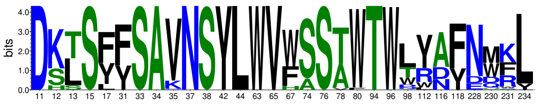

(m) special_rot bonus: -8.0

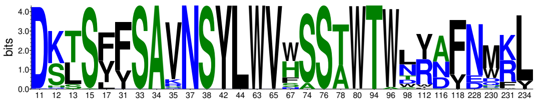

(n) special_rot bonus: -10.0

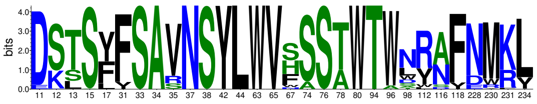

(o) special_rot bonus: Control

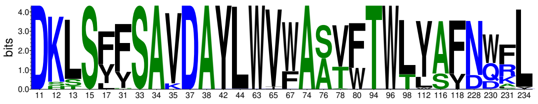

(p) special_rot bonus: Native

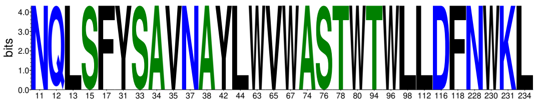

21 4AFG

(a) special_rot bonus: 0.0

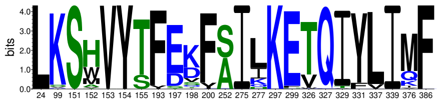

(b) special_rot bonus: -0.5

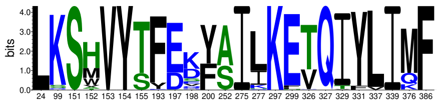

(c) special_rot bonus: -1.0

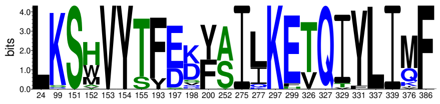

(d) special_rot bonus: -1.5

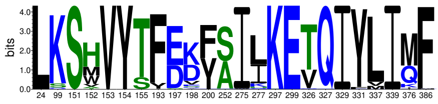

(e) special_rot bonus: -2.0

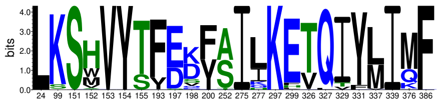

(f) special_rot bonus: -2.5

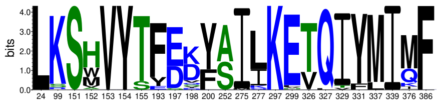

(g) special_rot bonus: -3.0

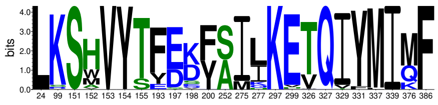

(h) special_rot bonus: -3.5

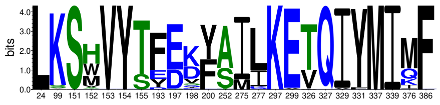

(i) special_rot bonus: -4.0

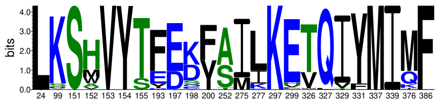

(j) special_rot bonus: -4.5

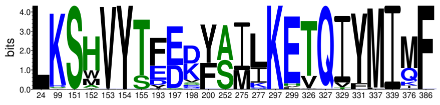

(k) special_rot bonus: -5.0

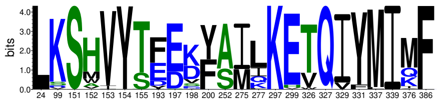

(l) special_rot bonus: -6.0

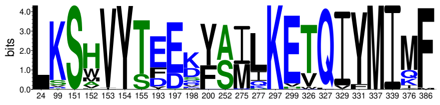

(m) special_rot bonus: -8.0

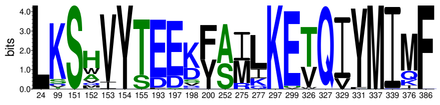

(n) special_rot bonus: -10.0

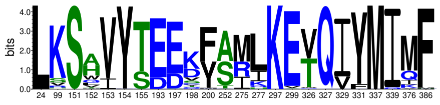

(o) special_rot bonus: Control

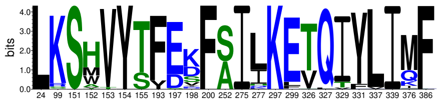

(p) special_rot bonus: Native

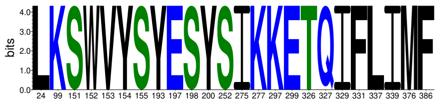

22 4AFH

(a) special_rot bonus: 0.0

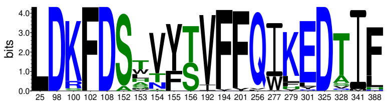

(b) special_rot bonus: -0.5

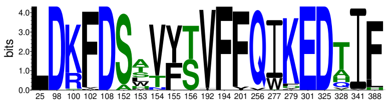

(c) special_rot bonus: -1.0

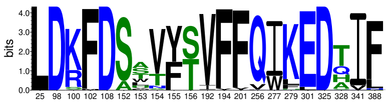

(d) special_rot bonus: -1.5

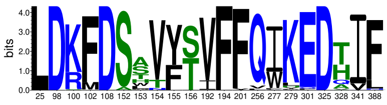

(e) special_rot bonus: -2.0

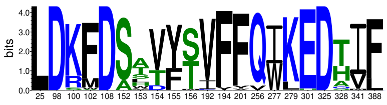

(f) special_rot bonus: -2.5

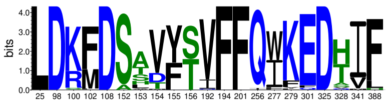

(g) special_rot bonus: -3.0

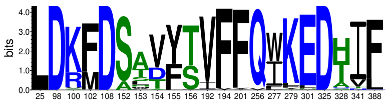

(h) special_rot bonus: -3.5

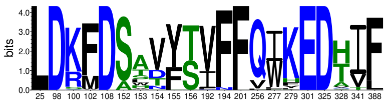

(i) special_rot bonus: -4.0

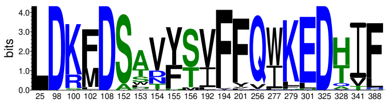

(j) special_rot bonus: -4.5

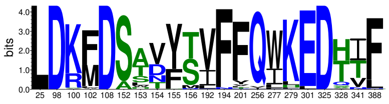

(k) special_rot bonus: -5.0

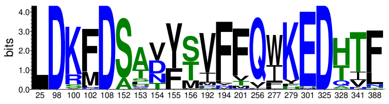

(l) special_rot bonus: -6.0

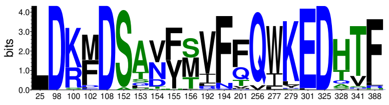

(m) special_rot bonus: -8.0

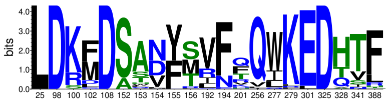

(n) special_rot bonus: -10.0

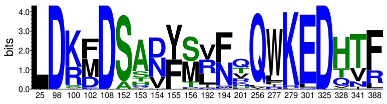

(o) special_rot bonus: Control

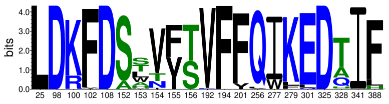

(p) special_rot bonus: Native

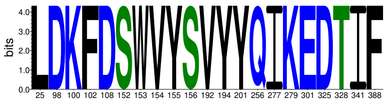

Supplement: S1 Appendix — Sequence logos generated for designable positions in existing complexes listed in S4 Table for various special_rot bonus values, where each logo represents 5000 design trajectories. A special_rot bonus of 0 indicates that complementary rotamers were added to the Packer, but received no score term bias. “Control” sequence logo was generated using the unmodified Packer. “Native” sequence logo shows the “correct” residue identity found in the native protein-ligand complex. Sequence logo positions are in Rosetta numbering corresponding to PDB files provided in S3 File. (PDF) [file pcbi.1008178.s005.pdf]
