# Supplementary material for: New computational protein design methods for de novo small molecule binding sites
Source: PLoS Comput Biol. 2020 Oct 5;16(10):e1008178. doi: 10.1371/journal.pcbi.1008178 (PMC7575090; doi:10.1371/journal.pcbi.1008178)

Design Metric Gridplots

Contents

1 UM_1_F153W228W36_40_2fnc_11_LFN_0001 2

2 UM_1_F24W123M71_84_2owpA_LFN_0001 3

3 UM_1_F51F138Y33_204_3gzbA_IM4_0002 4

4 UM_1_F74V71F18_5_1q40A_ATZ_0018 5

5 UM_1_K104F89F60_244_1tuhA_IBP_0019 6

6 UM_1_L11W14F18_9_1tp6A_ATZ_0022 7

7 UM_1_L21W41F124_2_1z1sA_IM4_0017 8

8 UM_1_L27L98L132_127_1jkgA_IM4_0007 9

9 UM_1_L27L98L132_174_1jkgA_IM4_0007 10

10 UM_1_R260W12W155_35_1jvx_11_IBP_0007 11

11 UM_1_R32I12V51_90_3ecfA_IBP_0004 12

12 UM_1_R93W62Y38_39_3gzrA_NPS_0005 13

13 UM_1_W27Y49F124_122_2rcdA_NPS_0005 14

14 UM_1_Y49W60L53_146_3er7B_DOG_0001 15

15 UM_2_F146V133M65_3_3emm_11_IM4_0018 16

1 UM_1_F153W228W36_40_2fnc_11_LFN_0001

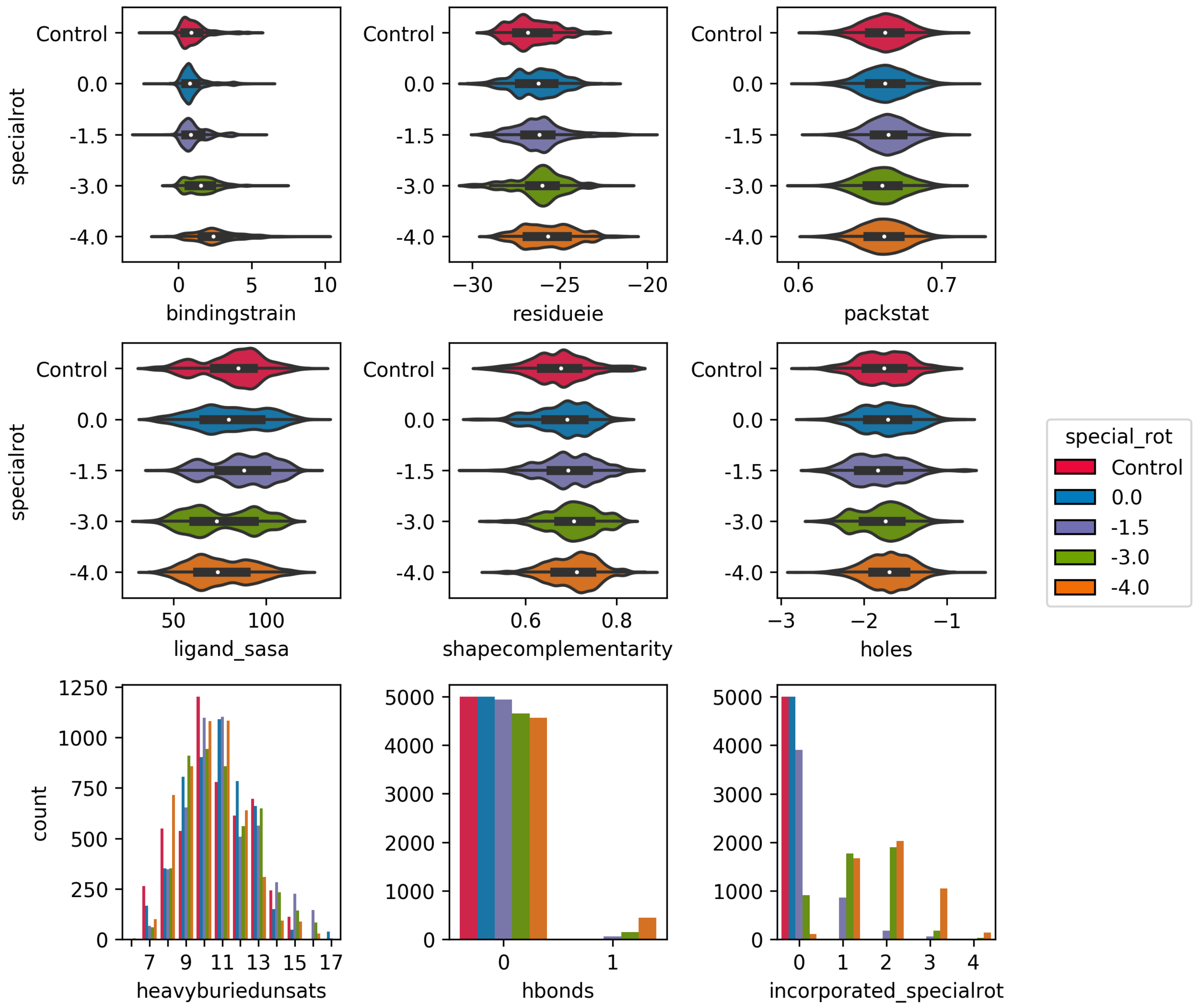

2 UM_1_F24W123M71_84_2owpA_LFN_0001

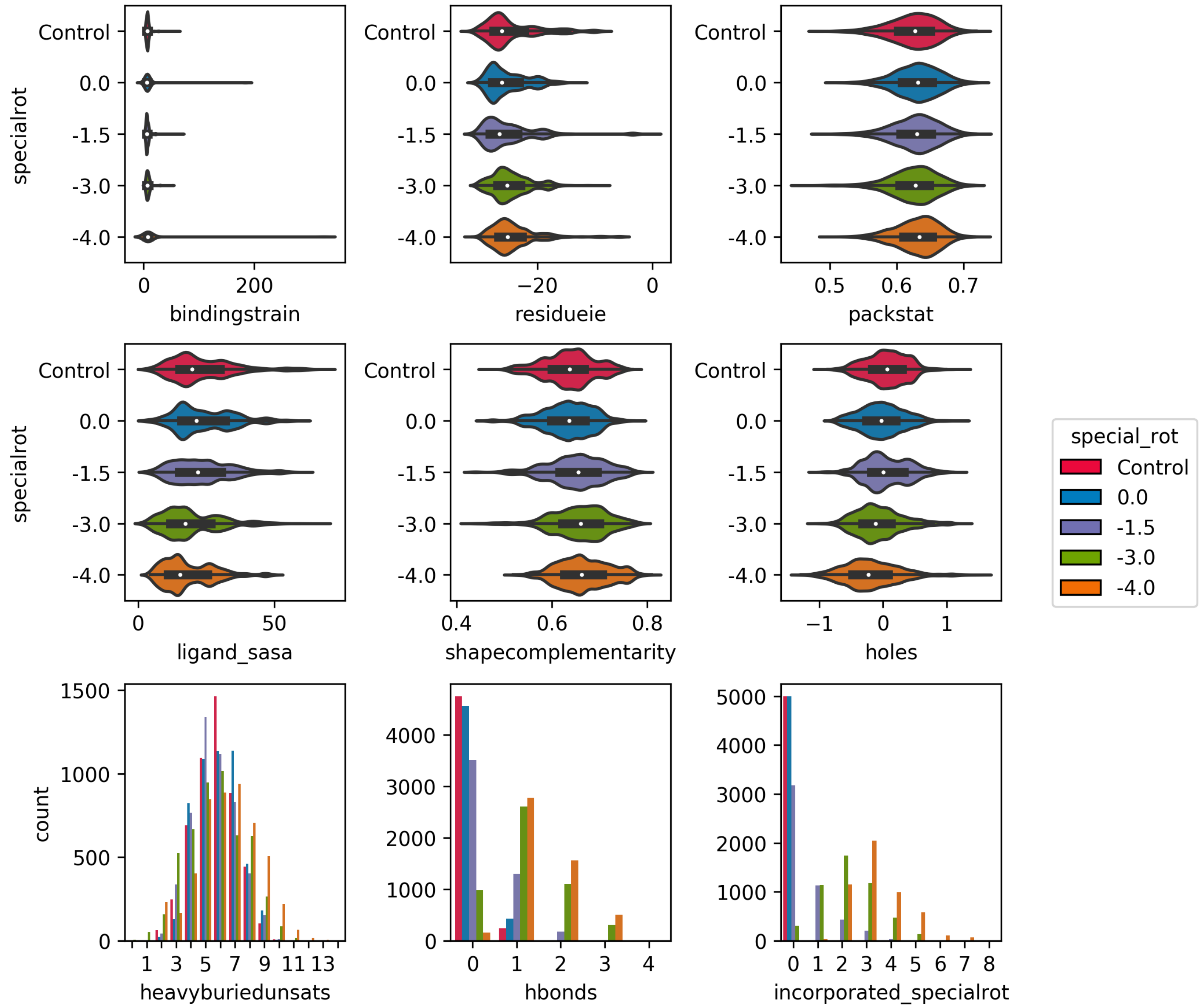

3 UM_1_F51F138Y33_204_3gzbA_IM4_0002

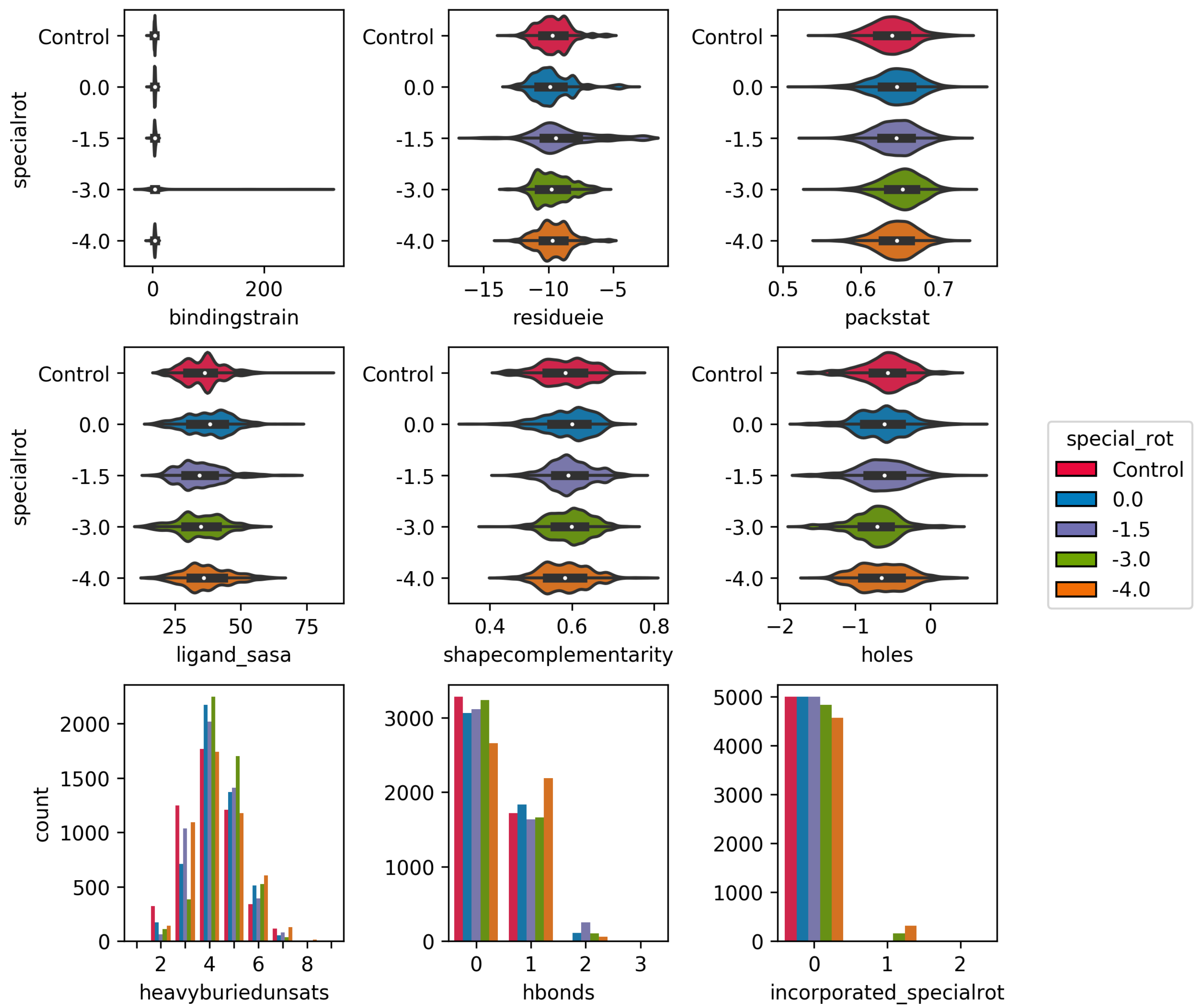

4 UM_1_F74V71F18_5_1q40A_ATZ_0018

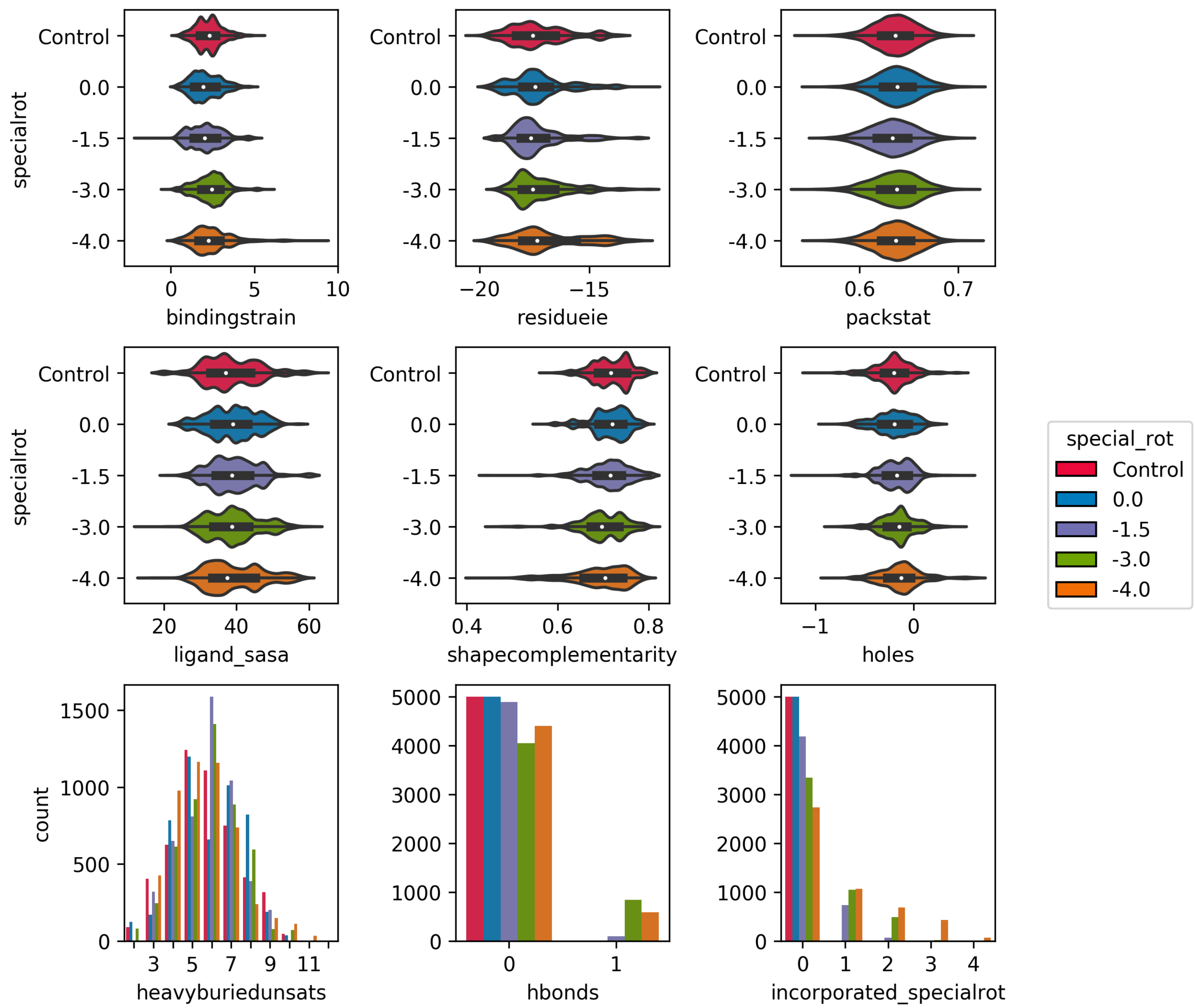

5 UM_1_K104F89F60_244_1tuhA_IBP_0019

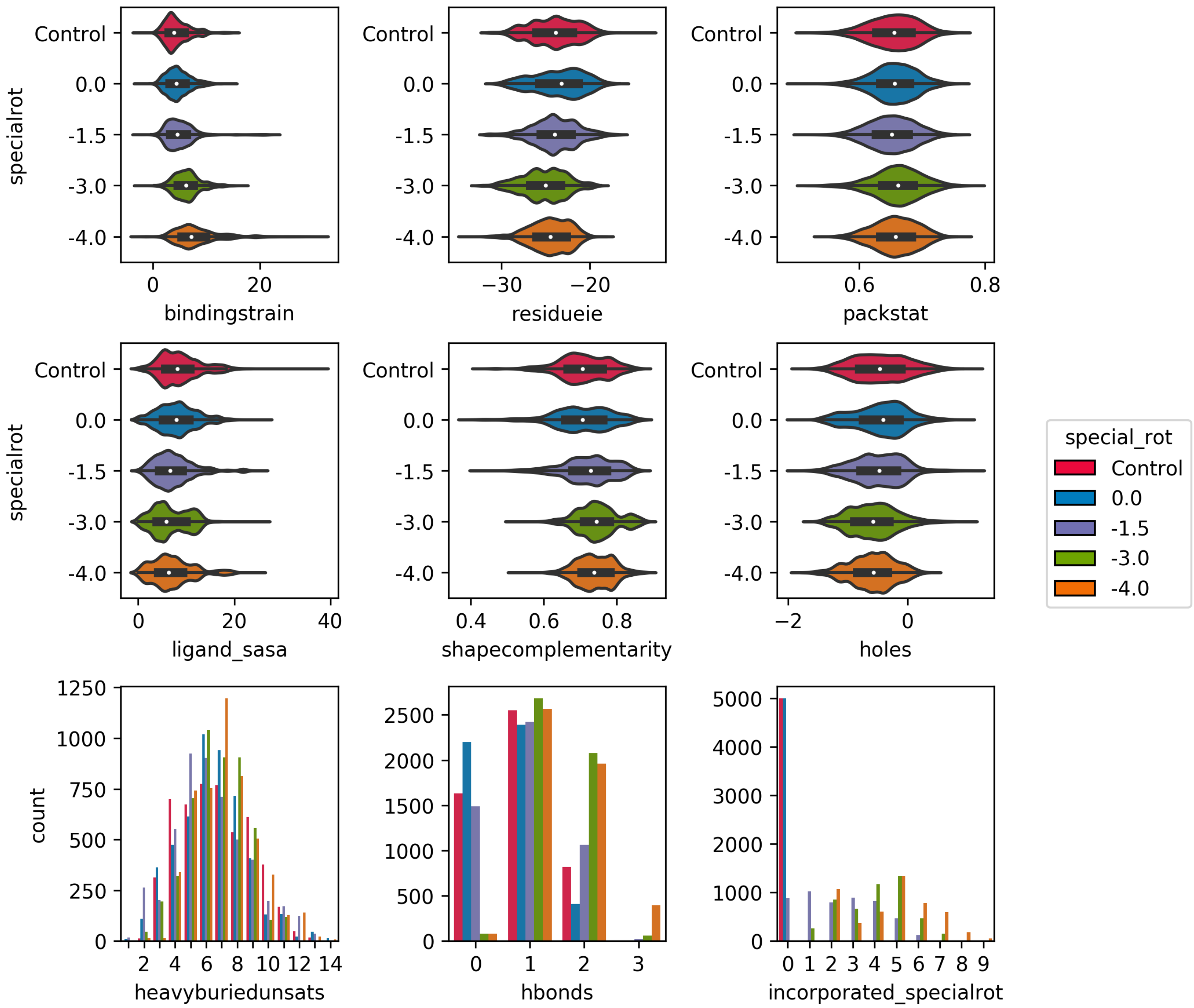

6 UM_1_L11W14F18_9_1tp6A_ATZ_0022

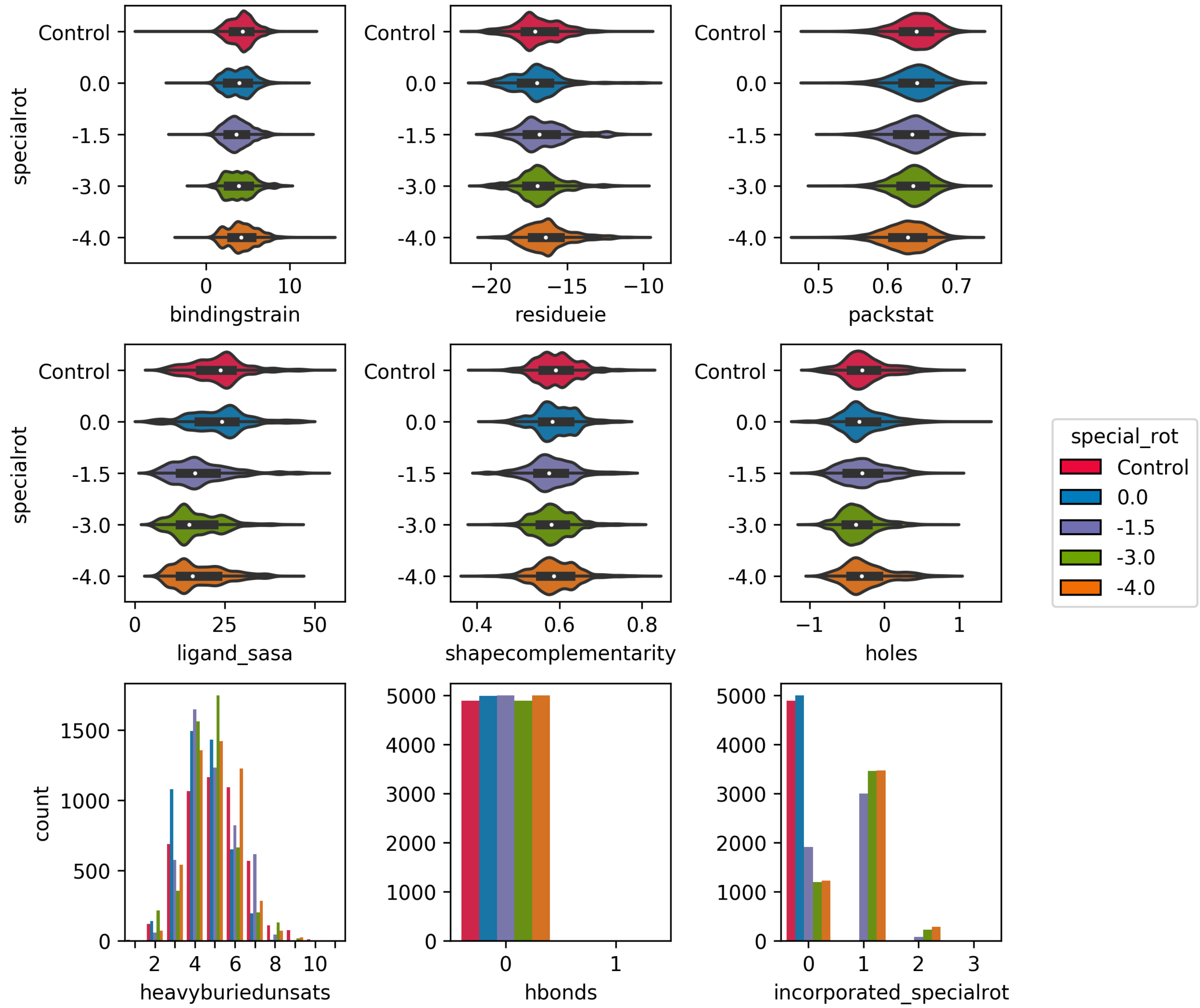

7 UM_1_L21W41F124_2_1z1sA_IM4_0017

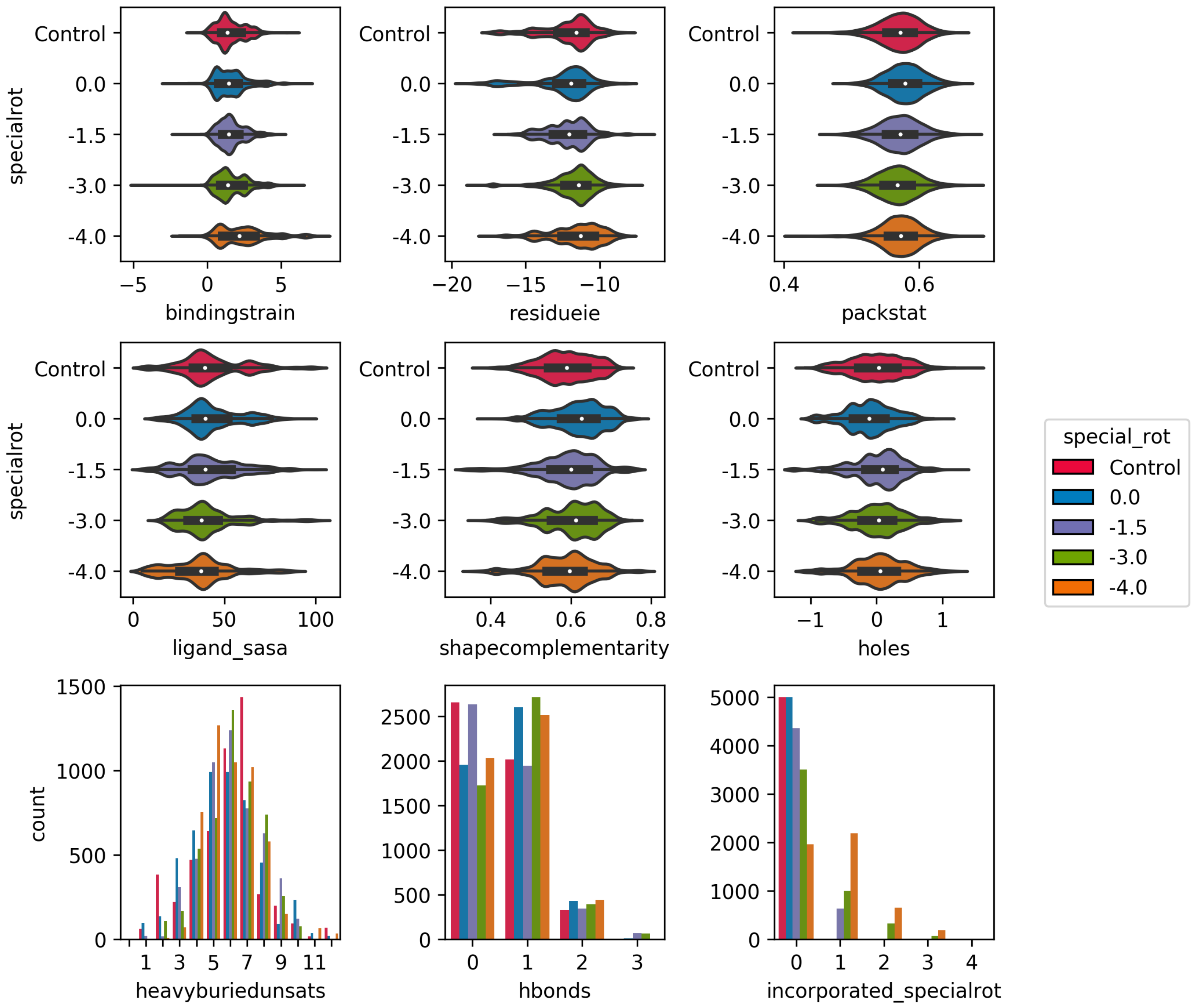

8 UM_1_L27L98L132_127_1jkgA_IM4_0007

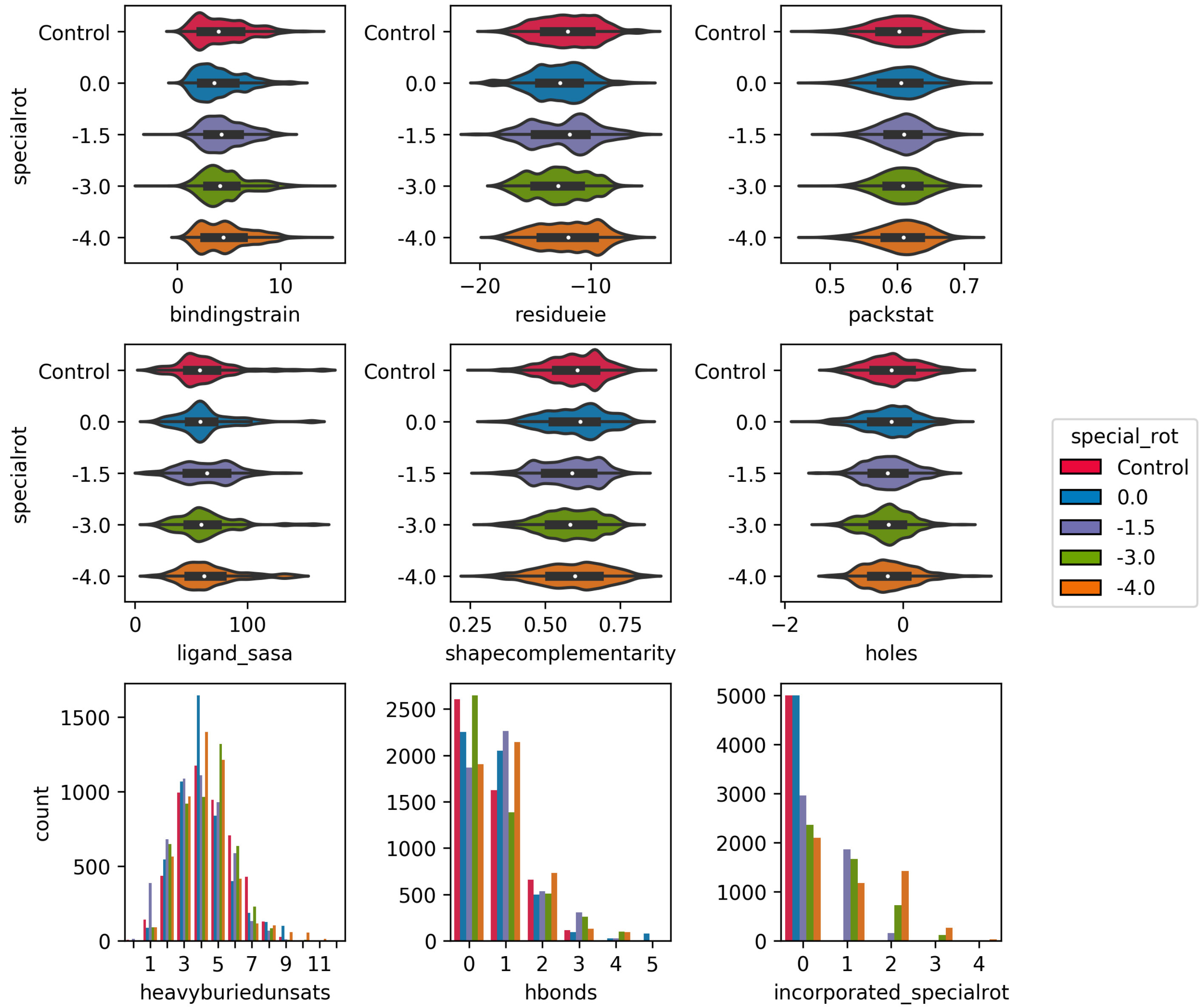

9 UM_1_L27L98L132_174_1jkgA_IM4_0007

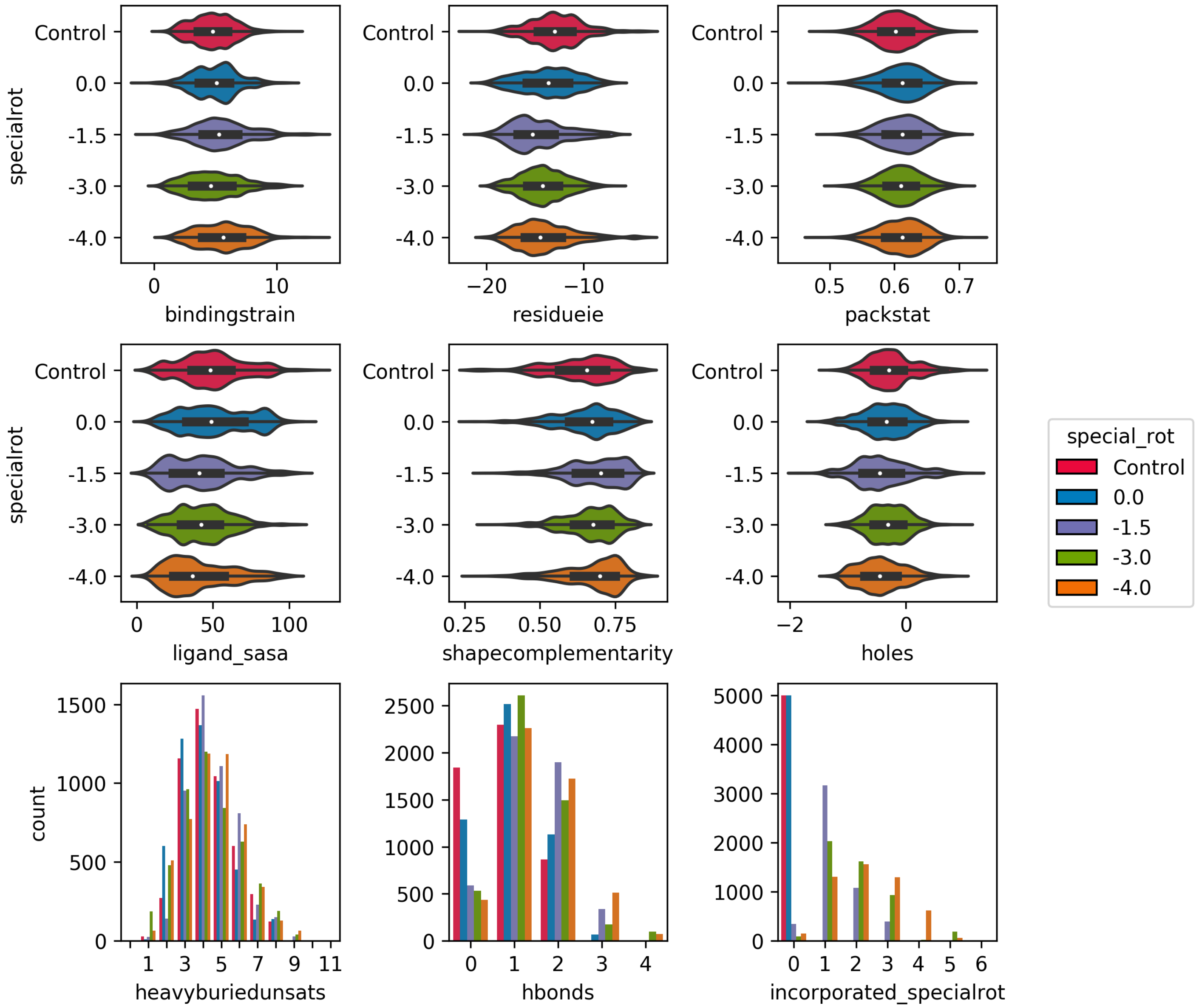

10 UM_1_R260W12W155_35_1jvx_11_IBP_0007

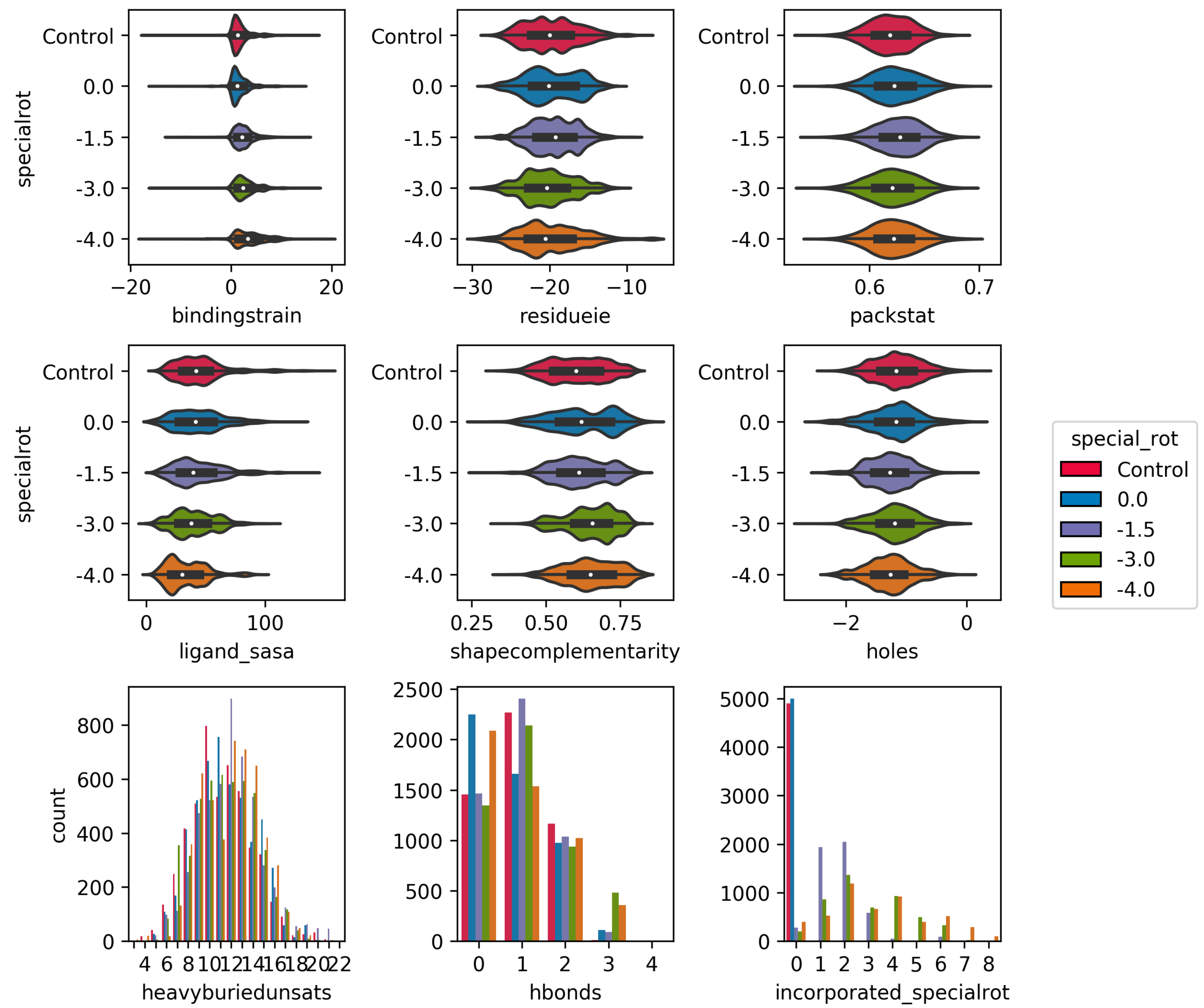

11 UM_1_R32I12V51_90_3ecfA_IBP_0004

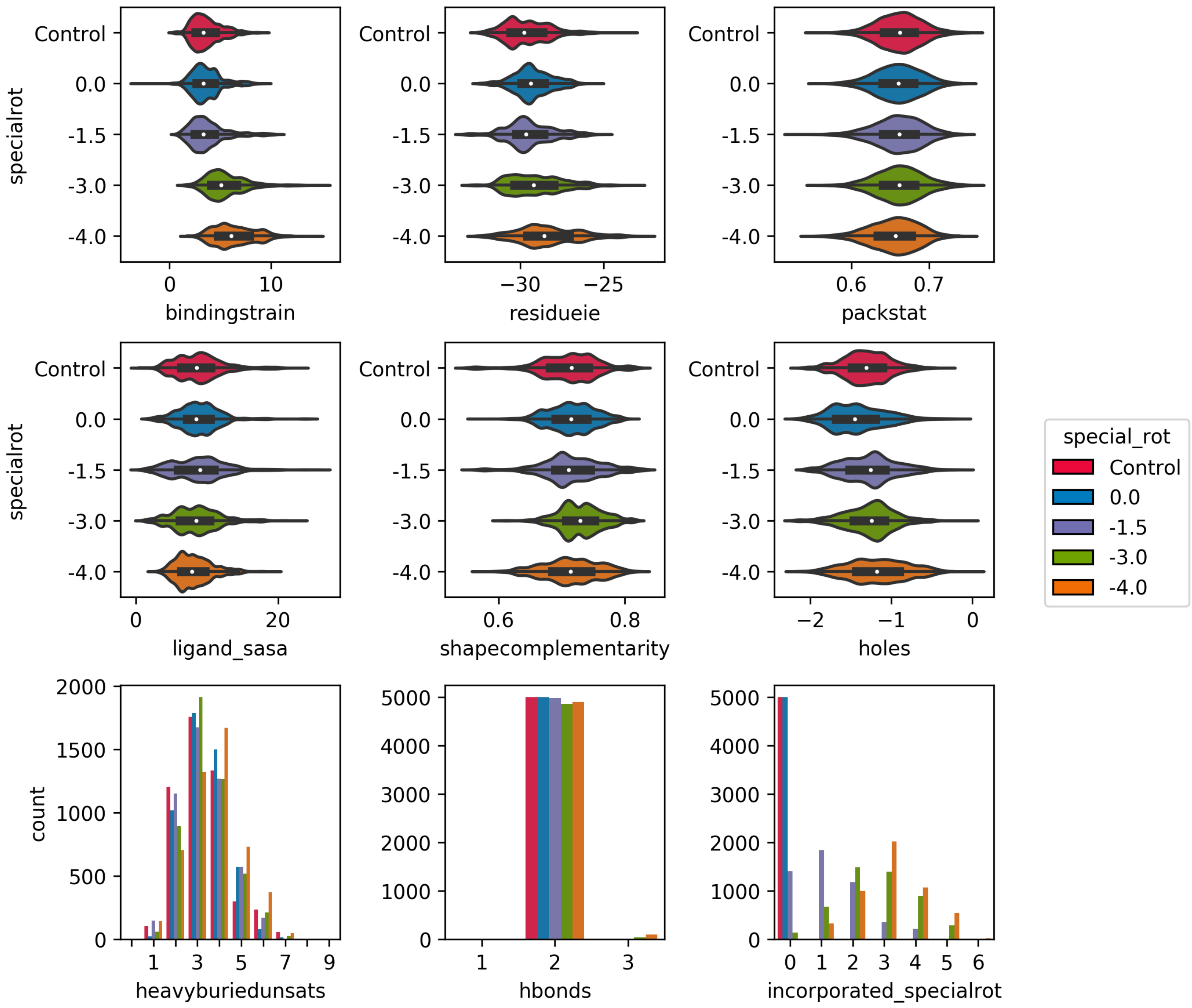

12 UM_1_R93W62Y38_39_3gzrA_NPS_0005

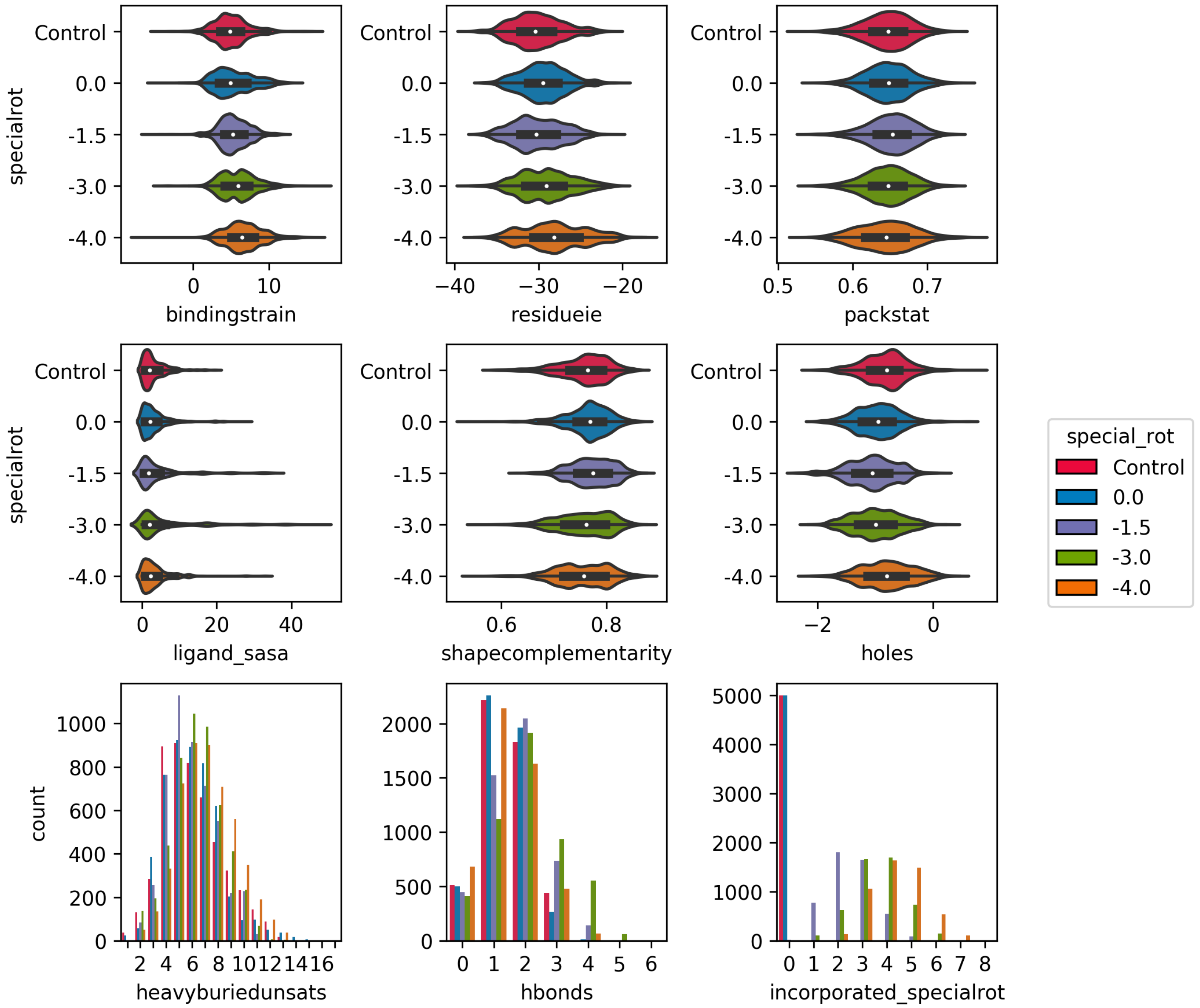

13 UM_1_W27Y49F124_122_2rcdA_NPS_0005

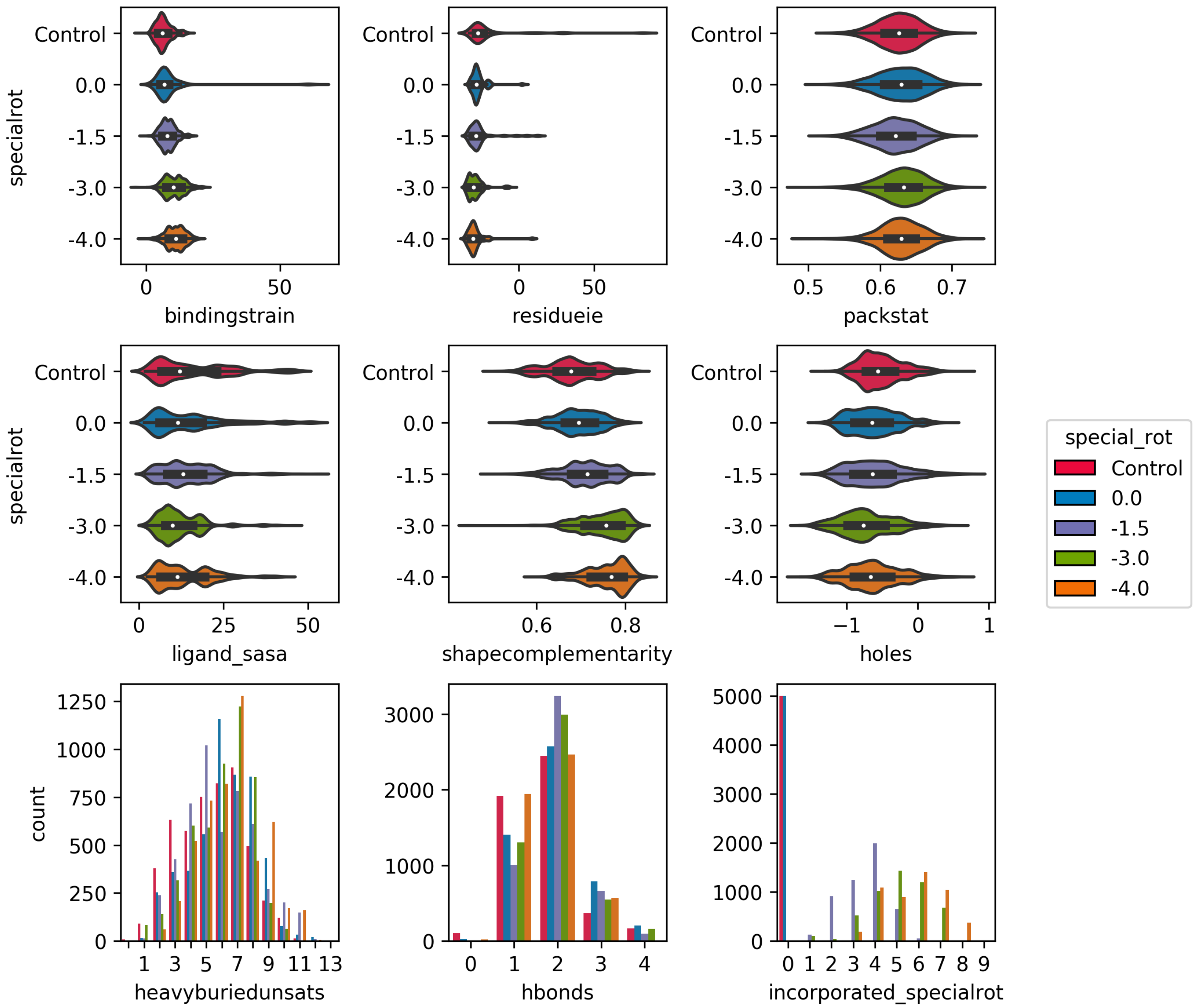

14 UM_1_Y49W60L53_146_3er7B_DOG_0001

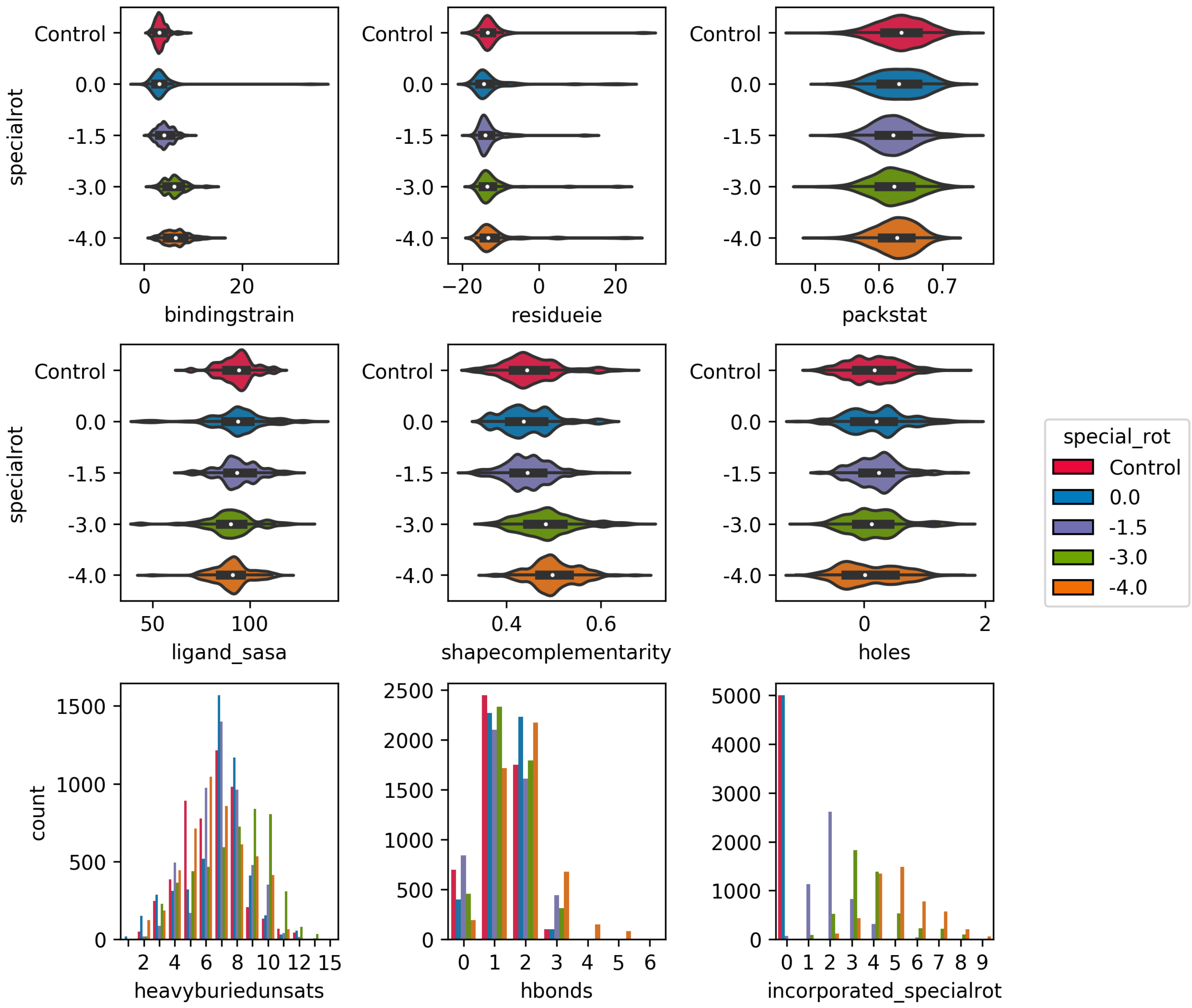

15 UM_2_F146V133M65_3_3emm_11_IM4_0018

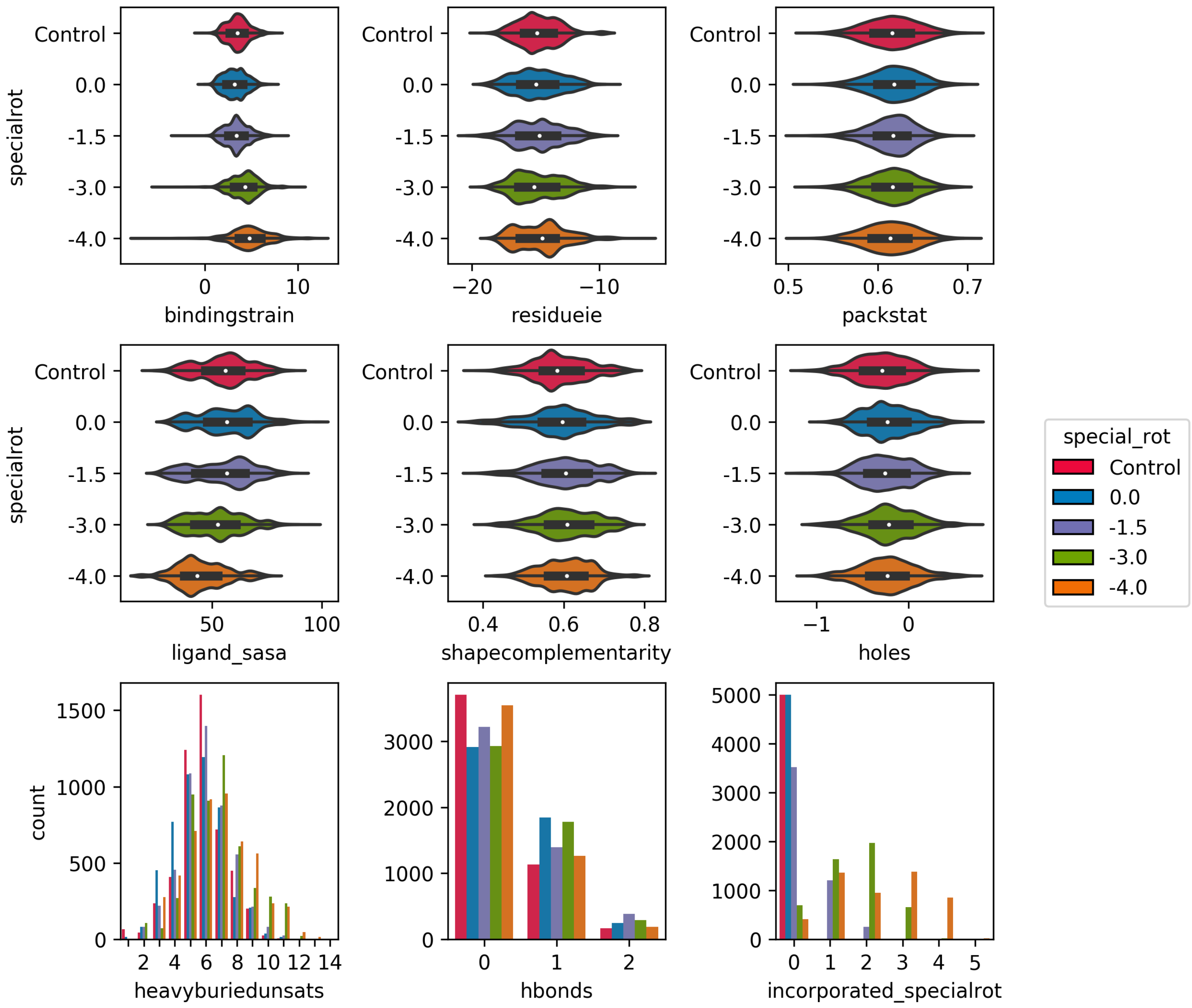

Supplement: S2 Appendix — Design metric distributions measured for 5000 forward design trajectories of selected matches listed in S5 Table. Existing Rosetta filters were applied to calculate binding strain (bindingstrain), interaction energy between the protein and ligand (residueie), packing statistics (Packstat), shape complementarity between the protein and ligand (shapecomplementarity), packing with RosettaHoles (holes), and the number of buried unsatisfied hydrogen bond donors/acceptors (heavyburiedunsats) for each design. Ligand solvent accessible surface area in Å2 (ligand_sasa), number of hydrogen bonds made between the protein and ligand (hbond), and number of complementary rotamers incorporated during design (incorporated_specialrot) were also calculated. (PDF) [file pcbi.1008178.s006.pdf]
