# Supplementary material for: New computational protein design methods for de novo small molecule binding sites
Source: PLoS Comput Biol. 2020 Oct 5;16(10):e1008178. doi: 10.1371/journal.pcbi.1008178 (PMC7575090; doi:10.1371/journal.pcbi.1008178)
Supplement: S1 Table — Kekule structures and corresponding SMILES strings are provided for all fragments used to investigate the availability of protein-fragment contact information in the PDB. The total number of unique residue-fragment contacts and the number of unique clusters (i.e. contact modes) represented by these residue-fragment contacts are reported. (DOCX) [file pcbi.1008178.s007.docx]

**S1 Table. Fragment SMILES Strings Used to Investigate Contact Diversity**

| **Fragment** | **SMILES** | **Total Residues** | **Total Clusters** | **Fraction PDB Sampled for >80% Contact Recovery** |
| --- | --- | --- | --- | --- |
| 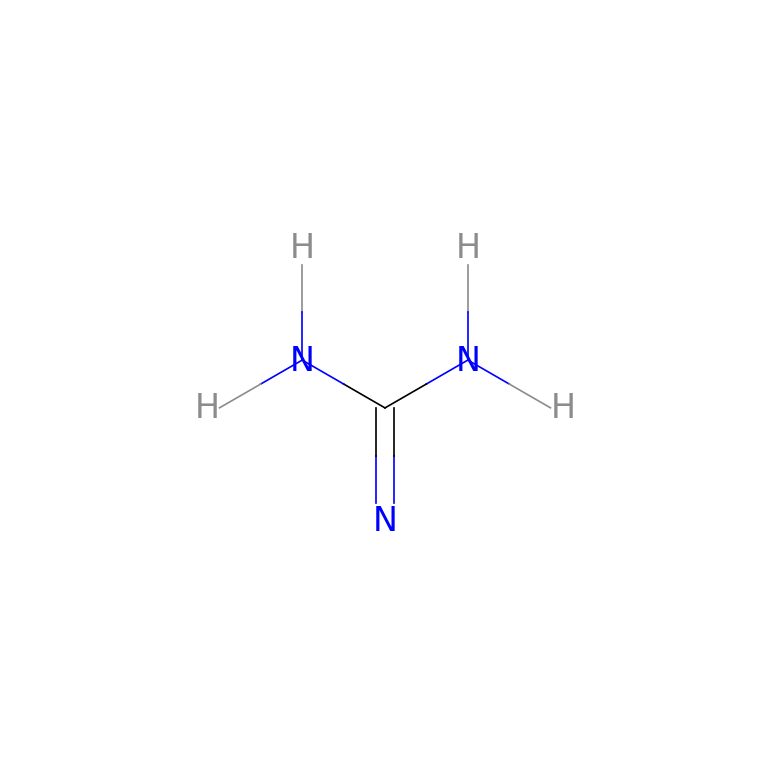 | [N;v3,v4]=C([N;v3]([H])[H])[N;v3]([H])[H] | 3699 | 467 | 0.45 |
| 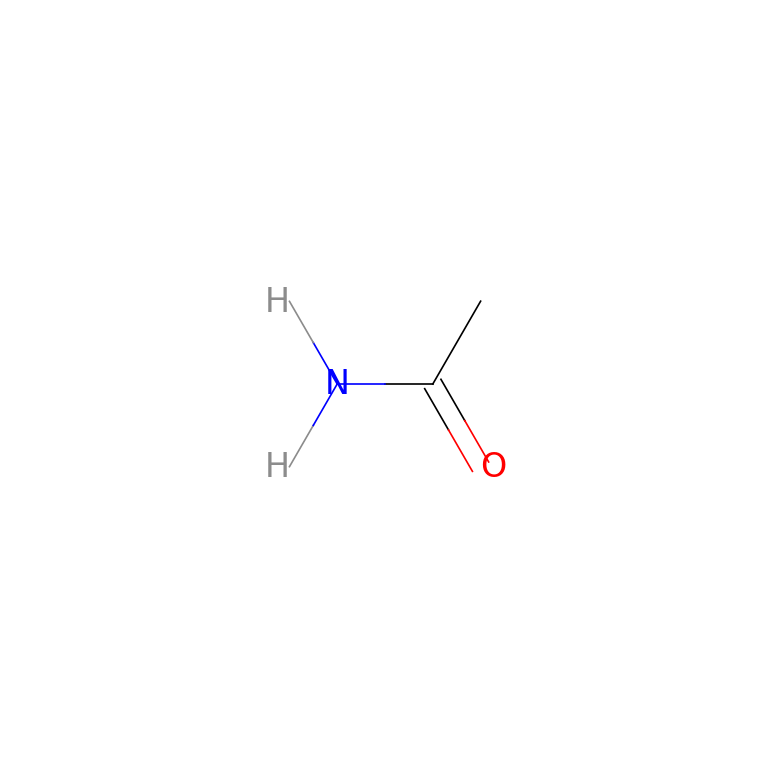 | CC(=O)N([H])[H] | 19077 | 932 | 0.35 |
| 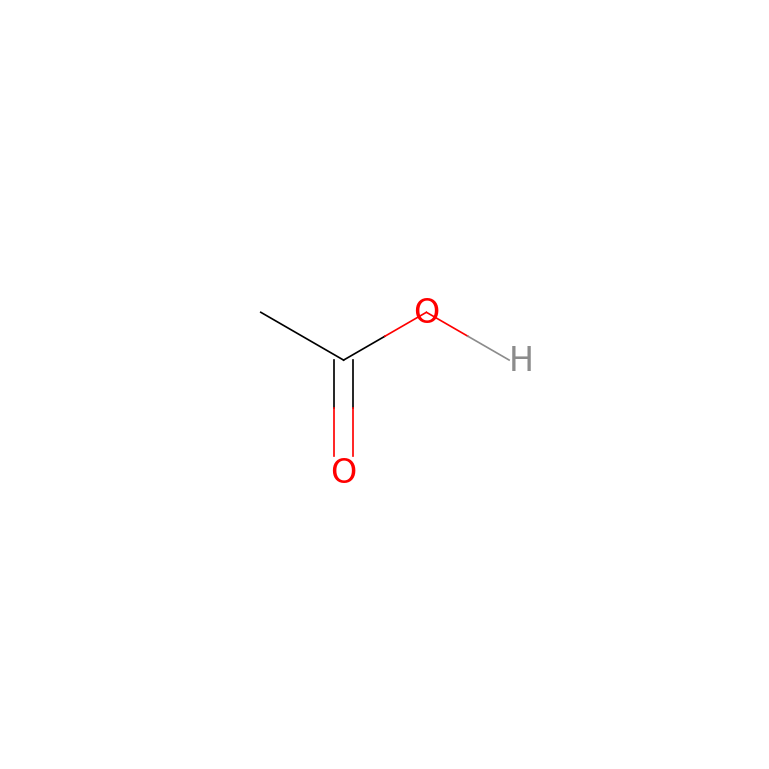 | CC(=O)O[H] | 64489 | 1699 | 0.20 |
| 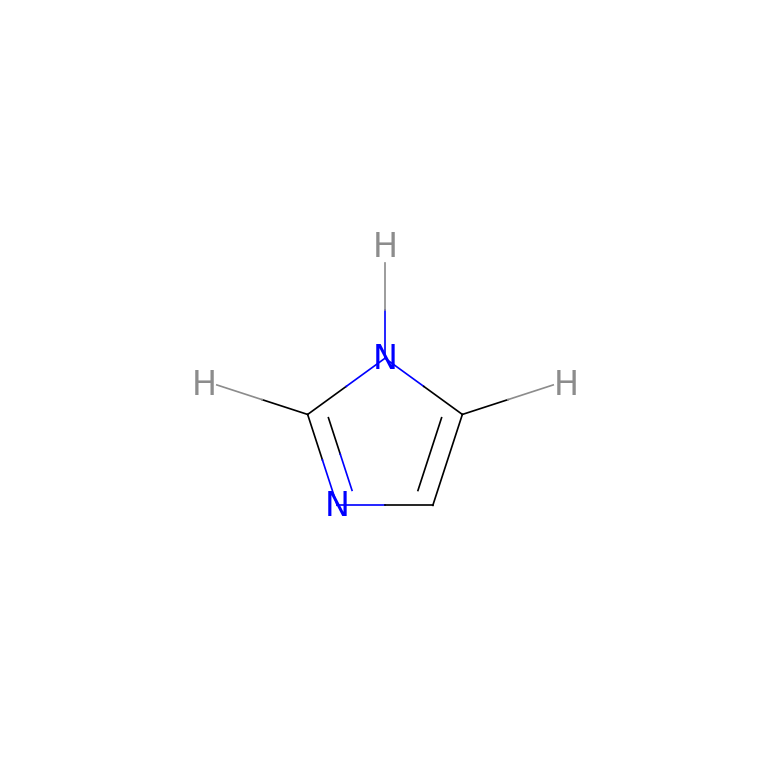 | C1=C([N](C(=N1)[H])[H])[H] | 3722 | 592 | 0.40 |
| 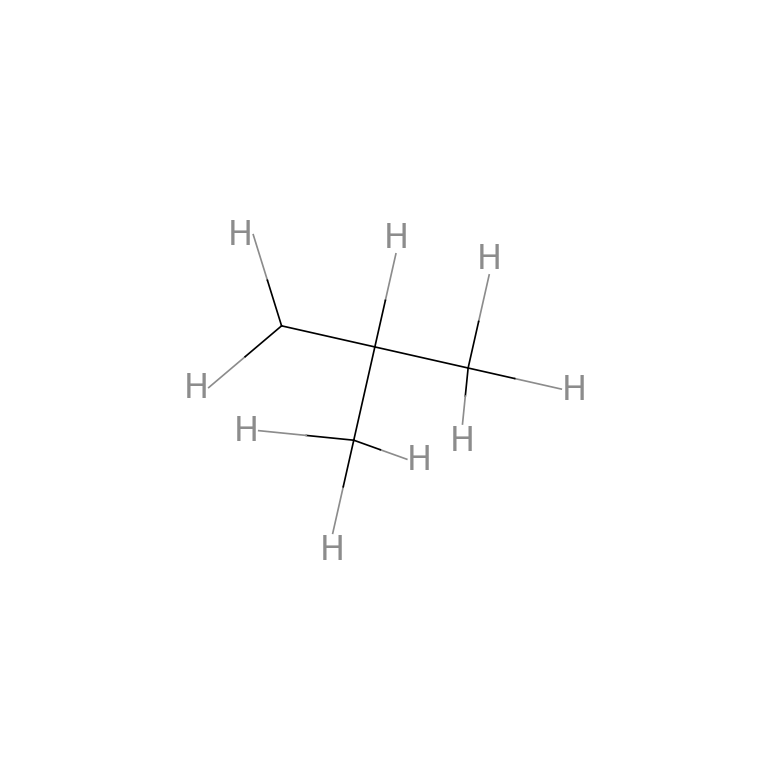 | C([H])([H])C([H])(C([H])([H])[H])C([H])([H])[H] | 6432 | 492 | 0.40 |
| 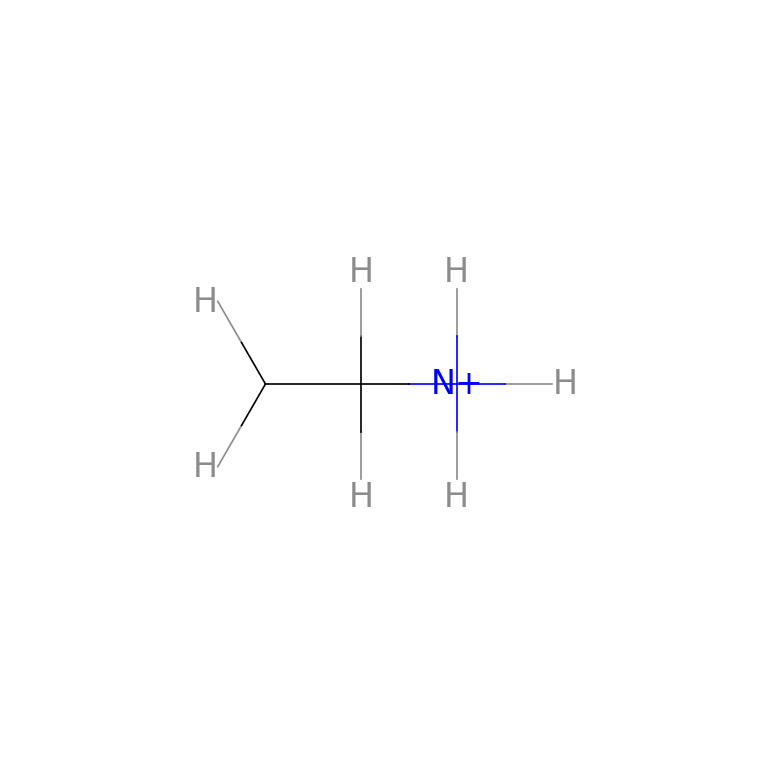 | C([H])([H])C([N+]([H])([H])[H])([H])[H] | 2814 | 442 | 0.50 |
| 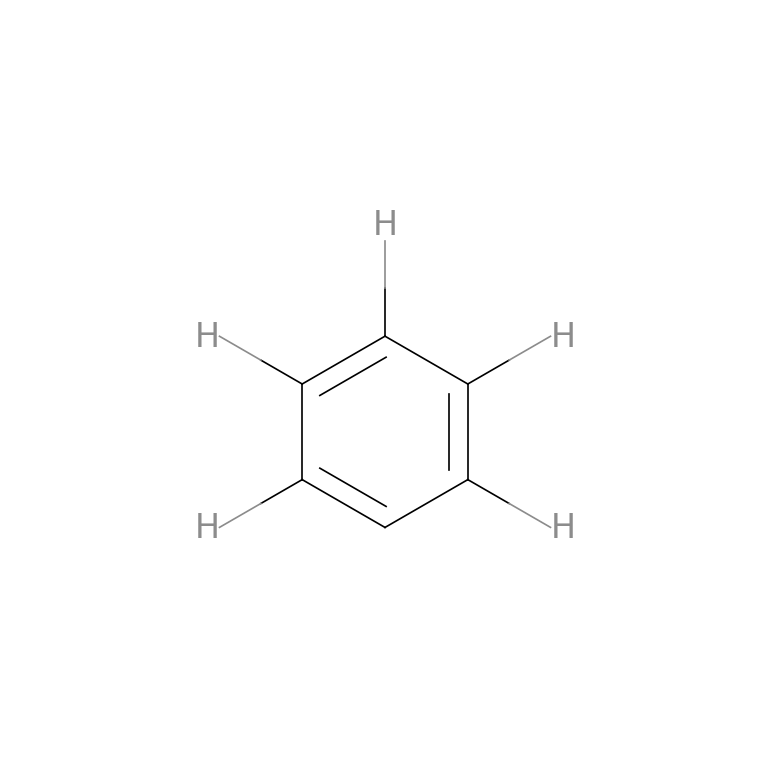 | C1=C(C(=C(C(=C1[H])[H])[H])[H])[H] | 10063 | 746 | 0.35 |
| 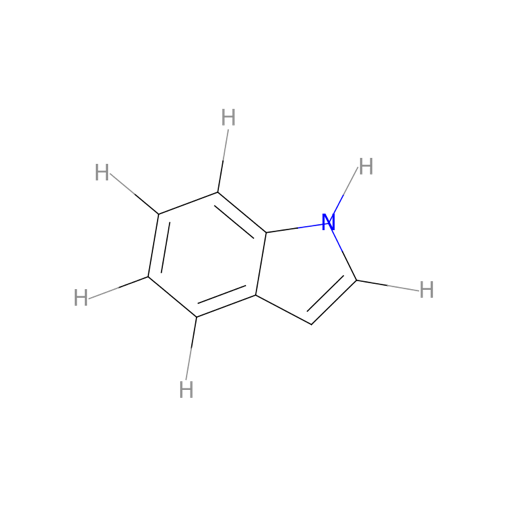 | C1=C([N]([H])C2=C([H])C(=C(C(=C12)[H])[H])[H])[H] | 2089 | 314 | 0.55 |
| 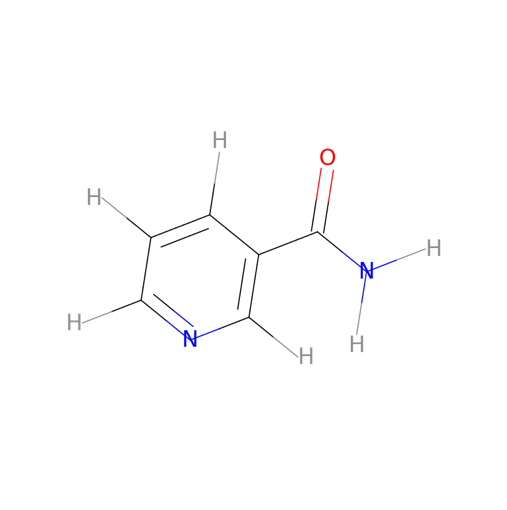 | N1=C(C(=C(C(=C1[H])C(=O)N([H])[H])[H])[H])[H] | 3414 | 319 | 0.45 |
| 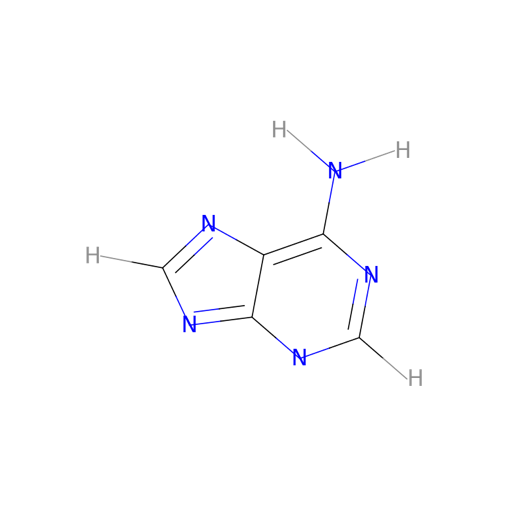 | N2=C1C(=C(N([H])[H])N=C([N]1)[H])N=C2[H] | 68520 | 1046 | 0.25 |
| 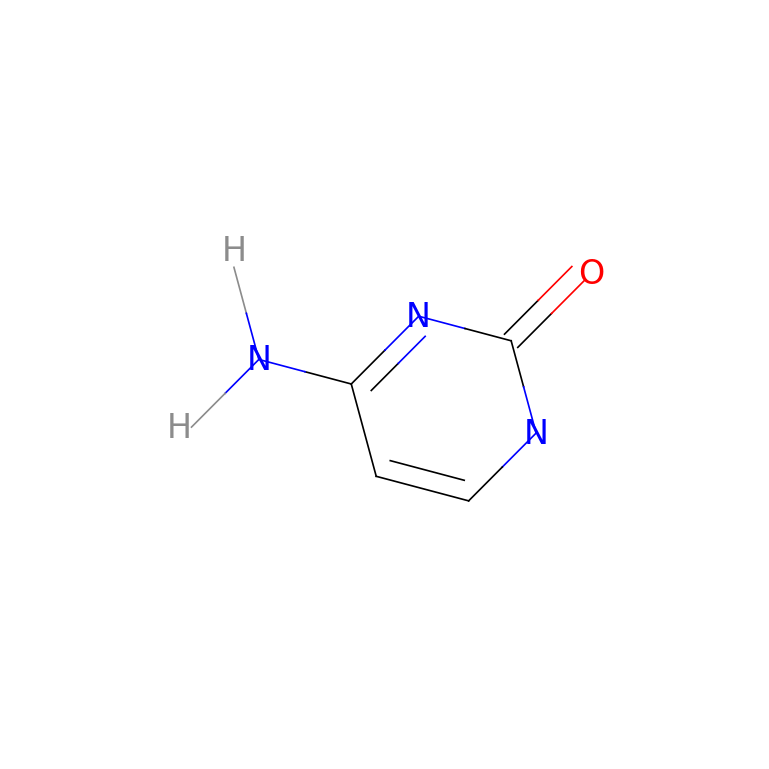 | N1C=CC(=NC1=O)N([H])[H] | 2123 | 282 | 0.50 |
| 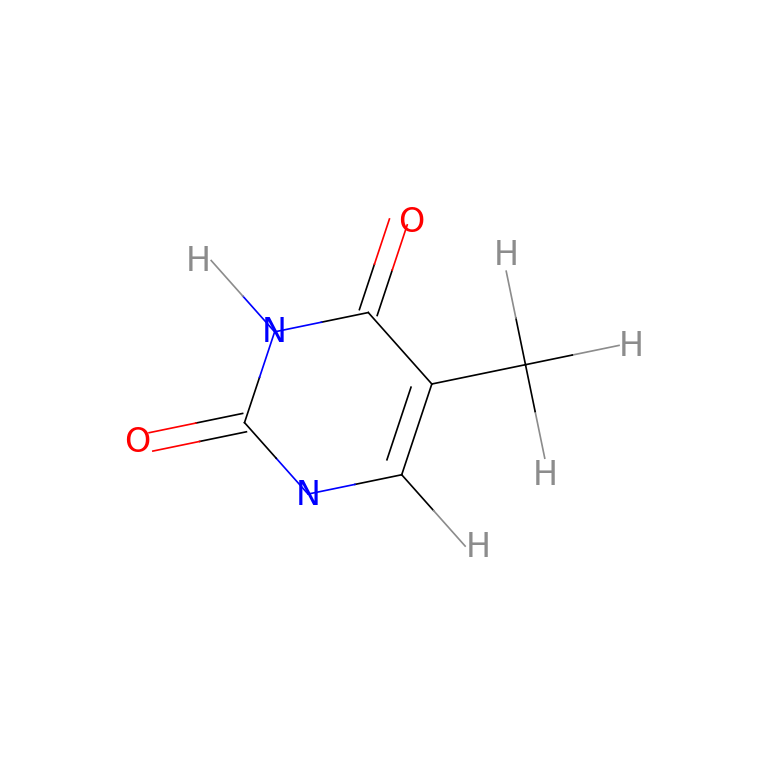 | N1C(=O)N(C(=O)C(=C1[H])C([H])([H])[H])[H] | 2116 | 228 | 0.50 |
| 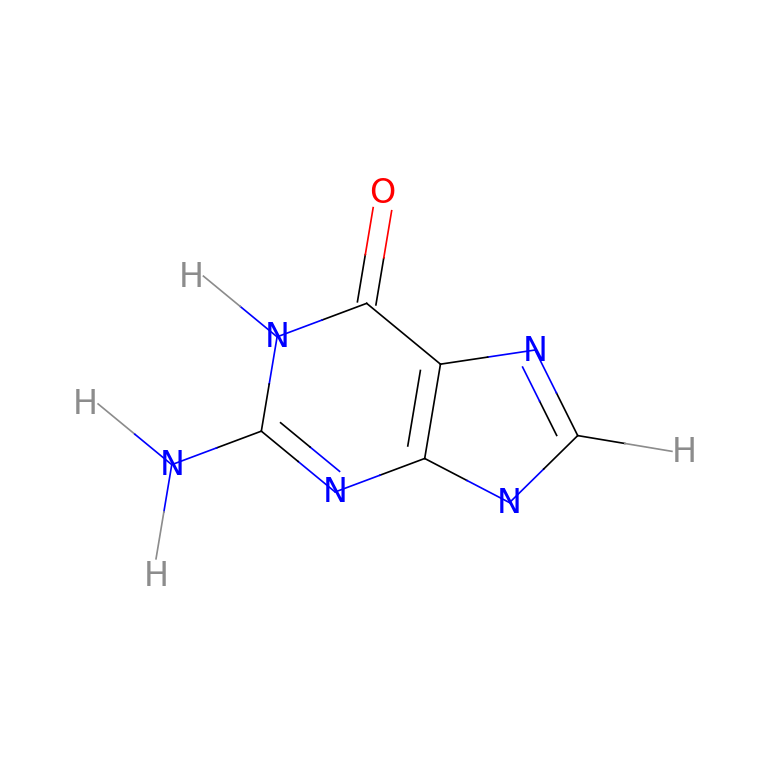 | [N]1C(=NC2=C1N=C(N(C2=O)[H])N([H])[H])[H] | 12387 | 580 | 0.40 |
| 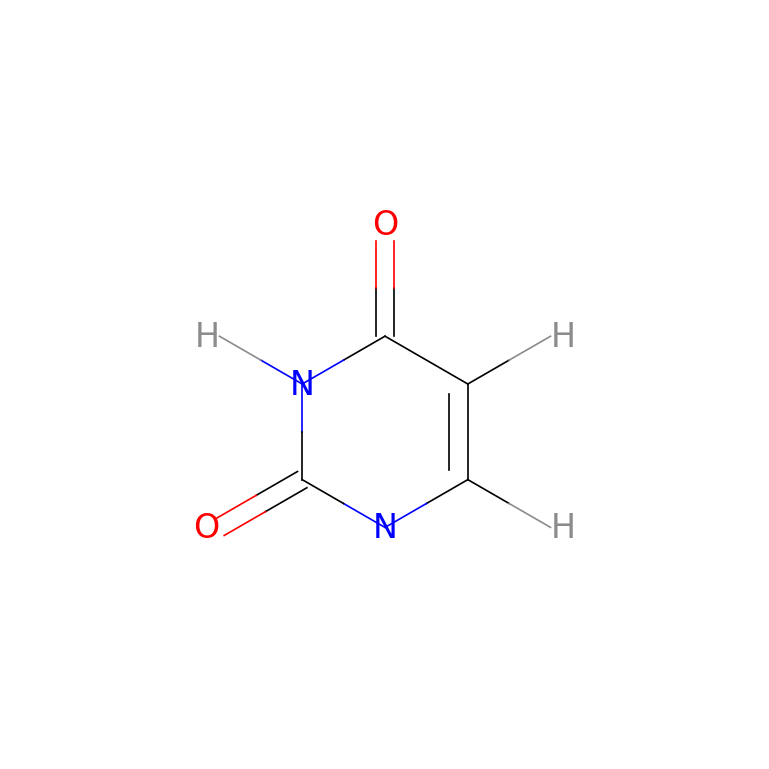 | N1C(N(C(C(=C1[H])[H])=O)[H])=O | 6040 | 440 | 0.45 |
| 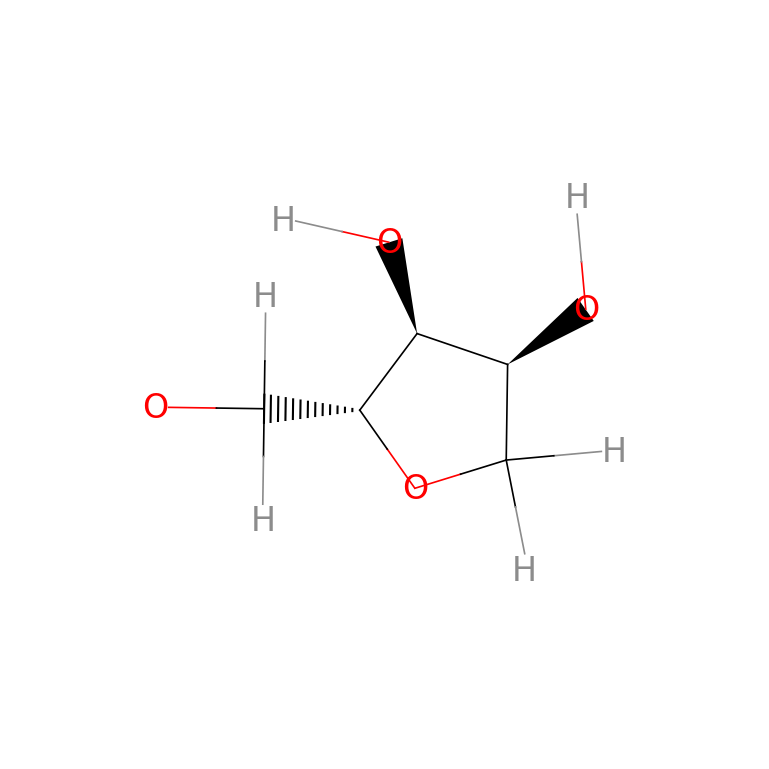 | [C]1([C@H](O[H])[C@@H]([C@H](O1)C(O)([H])[H])O[H])([H])[H] | 11621 | 754 | 0.35 |
| 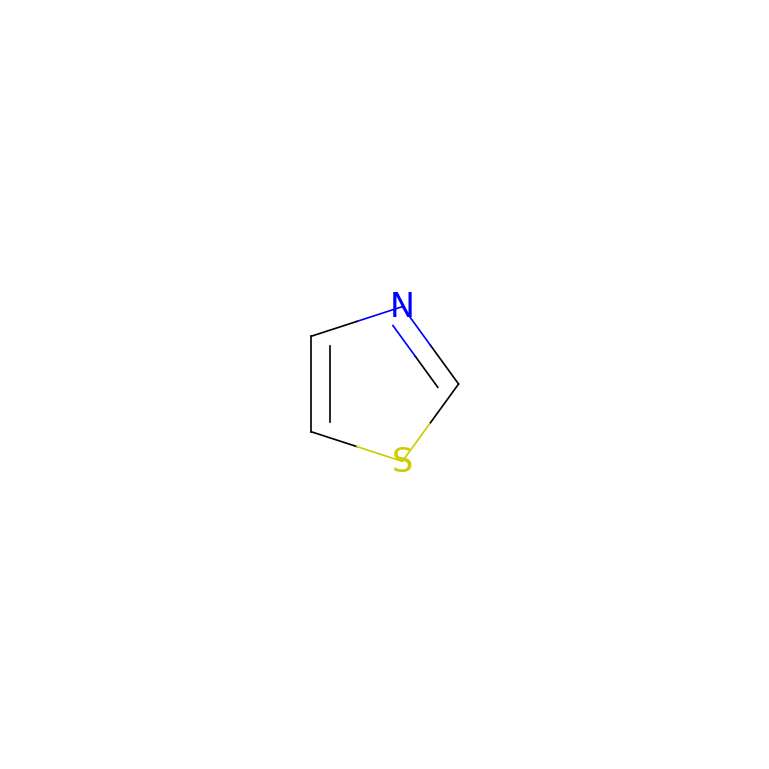 | C1=NC=CS1 | 1573 | 215 | 0.55 |
| 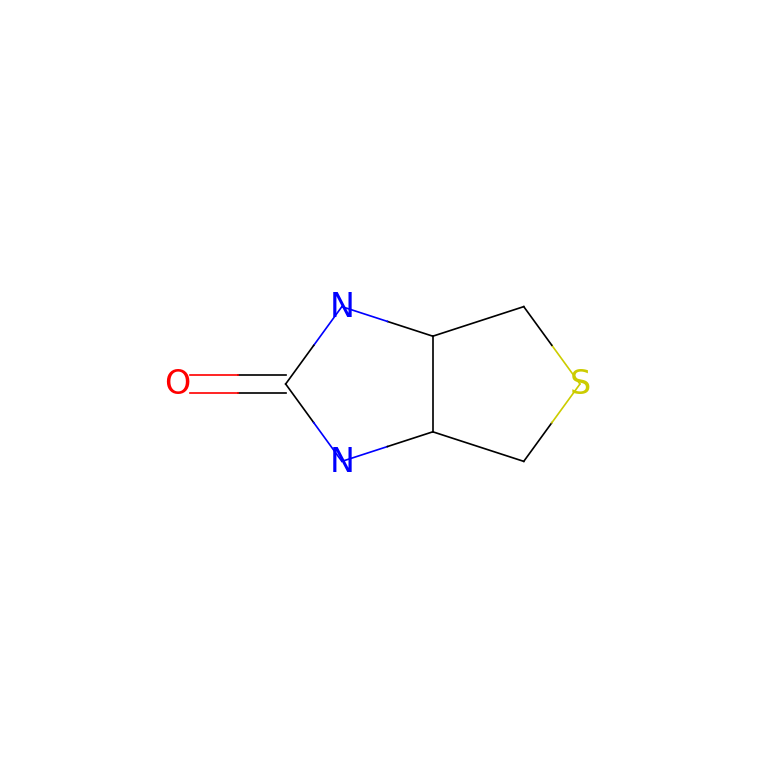 | C1SCC2NC(=O)NC12 | 1051 | 34 | 0.20 |
| 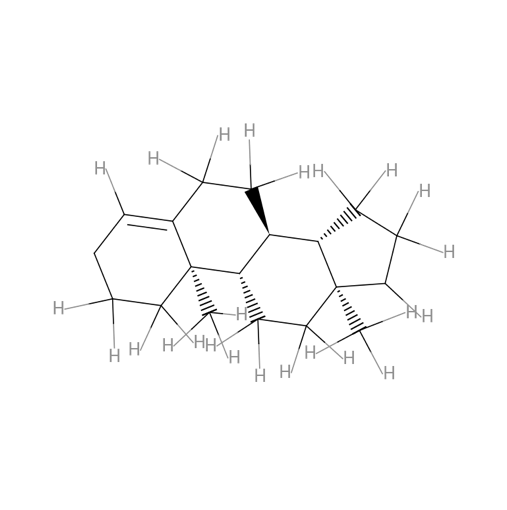 | C1(C(CC(=C2C(C([C@@H]3[C@@H]([C@@]12C([H])([H])[H])C(C([C@]4([C@H]3C(C(C4[H])([H])[H])([H])[H])C([H])([H])[H])([H])[H])([H])[H])([H])[H])([H])[H])[H])([H])[H])([H])[H] | 511 | 102 | 0.55 |
| 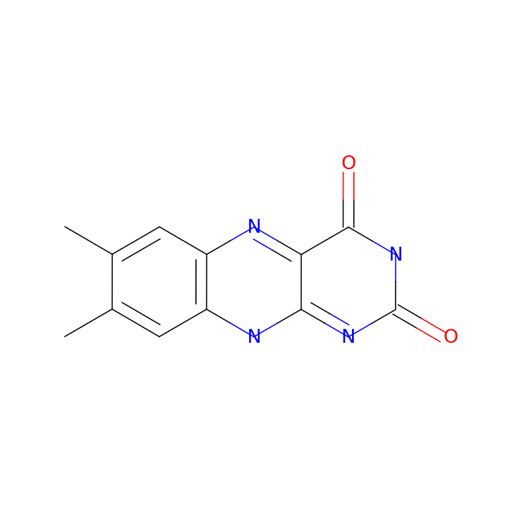 | N3=C2C(=NC1=C(C=C(C(=C1)C)C)N2)C(NC3=O)=O | 21494 | 685 | 0.30 |
| 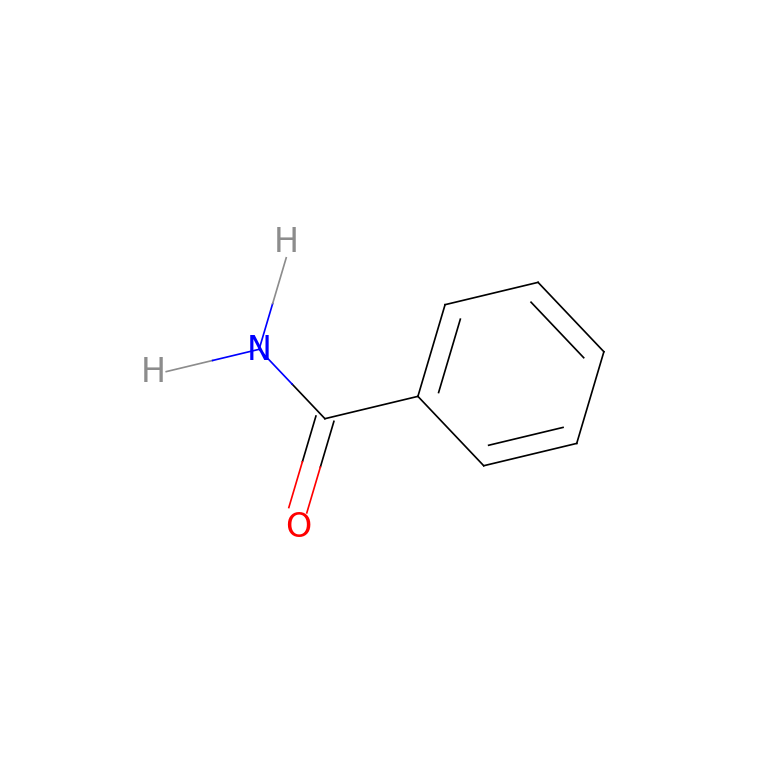 | [C]1=CC(=[C][C]=[C]1)C(=O)N([H])[H] | 284 | 84 | 0.65 |
| 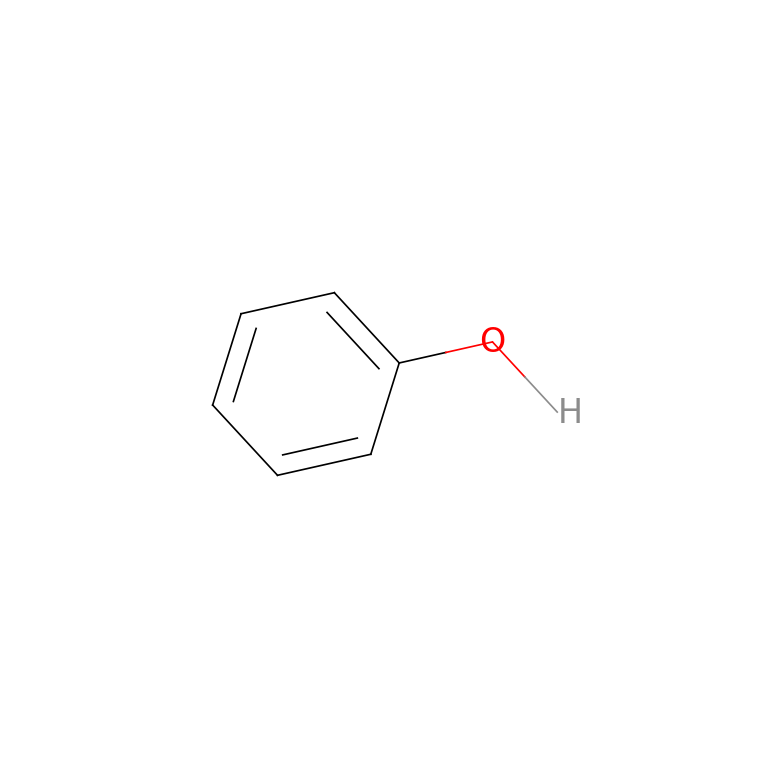 | C1=CC=C(C=C1)O[H] | 12964 | 896 | 0.35 |
| 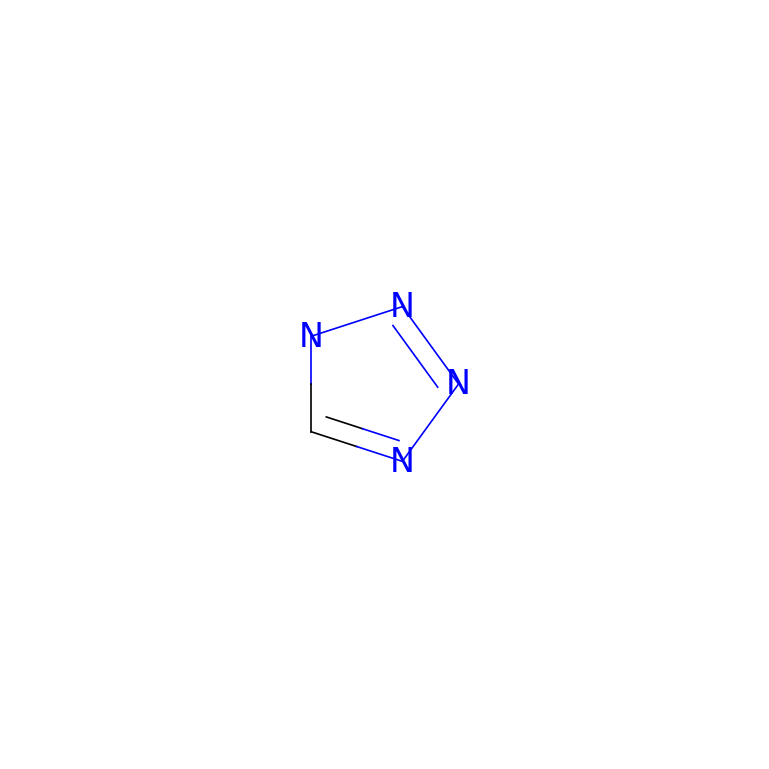 | N1=N[N]C=N1 | 166 | 52 | 0.65 |
| 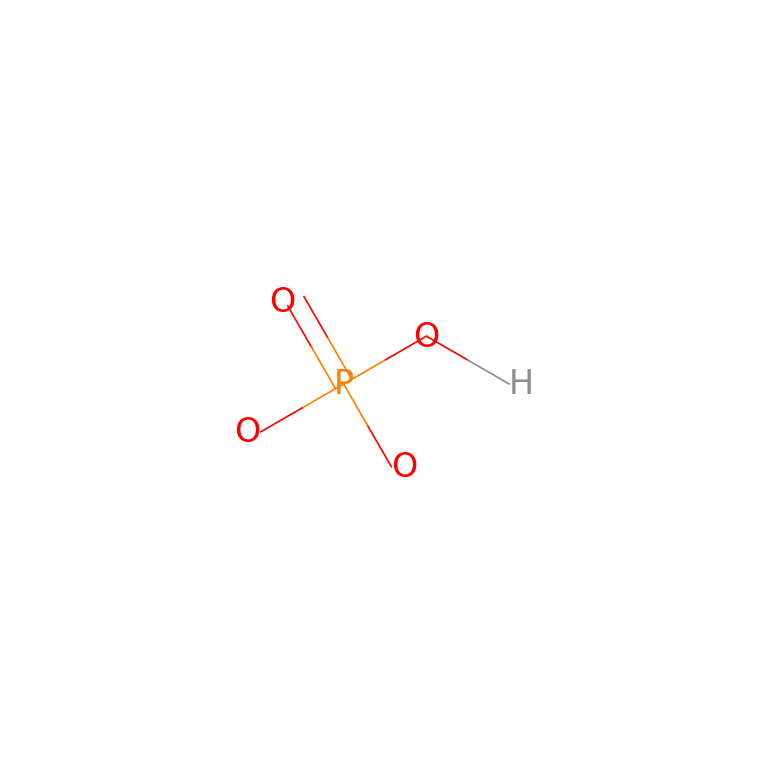 | O=[P](O)(O[H])O | 78948 | 1371 | 0.15 |
| 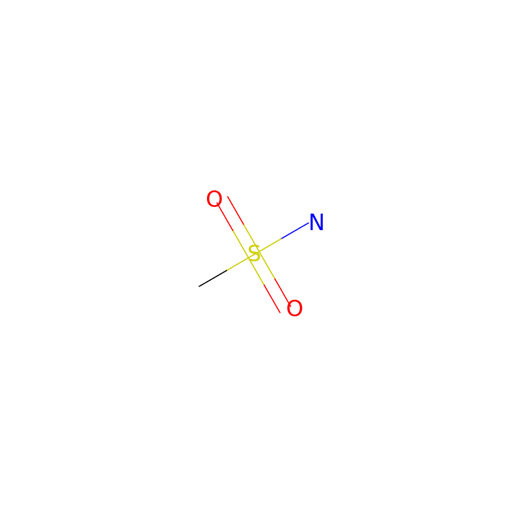 | [S](=O)(=O)(N)C | 1631 | 252 | 0.45 |
| 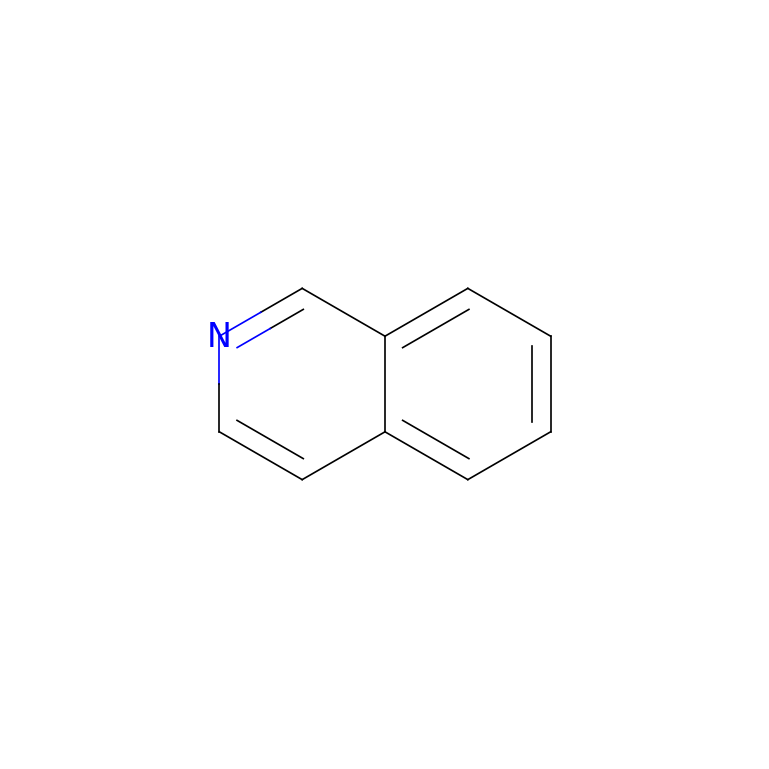 | C1=C2C(=CC=C1)C=NC=C2 | 533 | 126 | 0.60 |
| 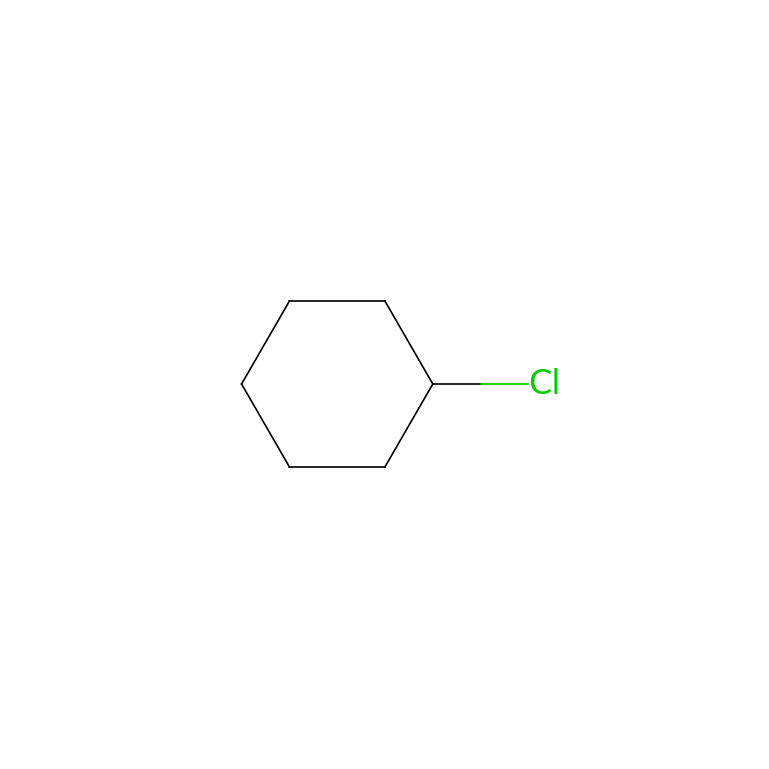 | C1=CC(=CC=C1)[Cl] | 7592 | 599 | 0.40 |
| 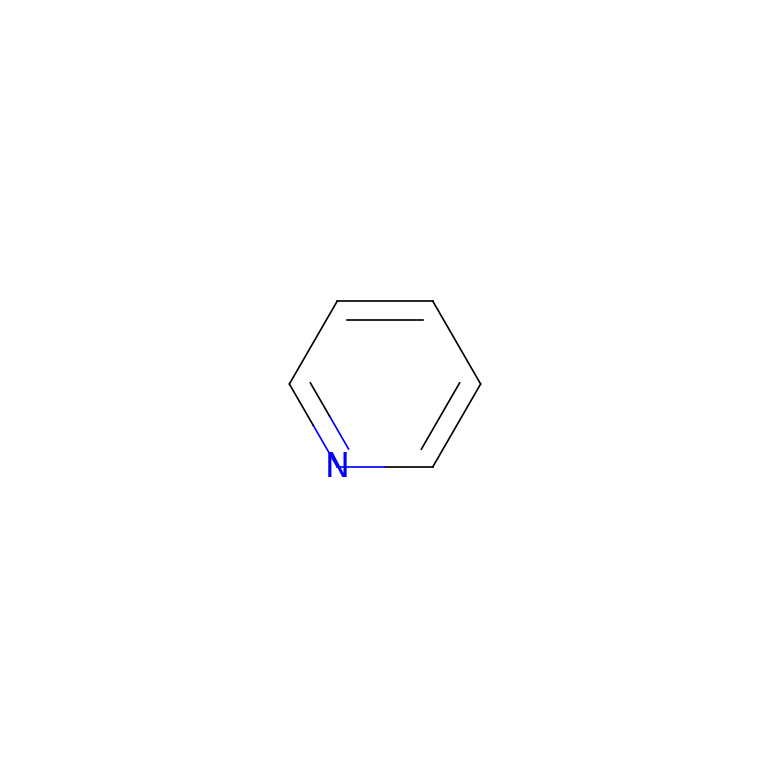 | C1=CN=CC=C1 | 17058 | 755 | 0.35 |
| 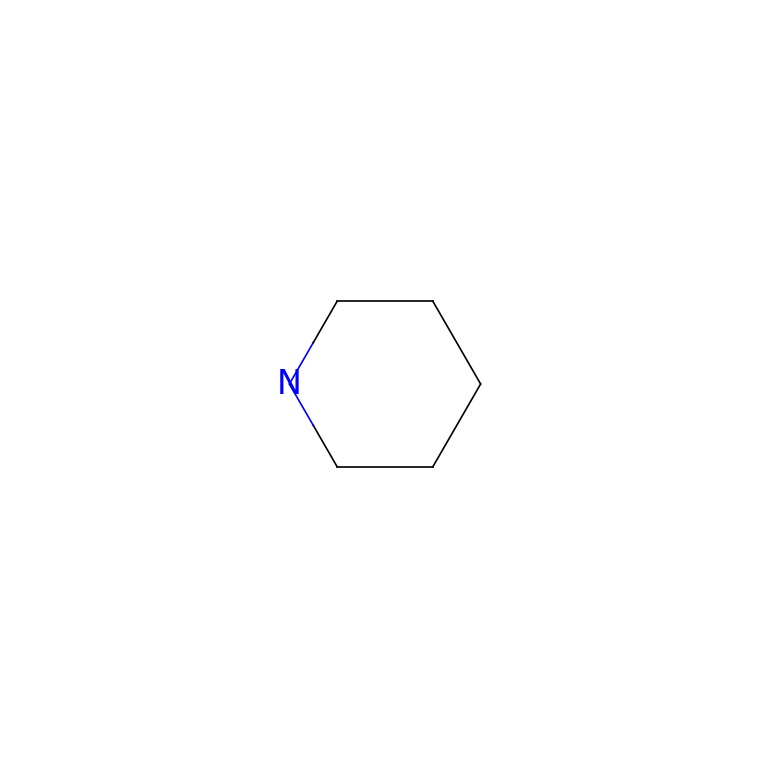 | [C]1[C][C][N][C][C]1 | 18883 | 811 | 0.35 |
| 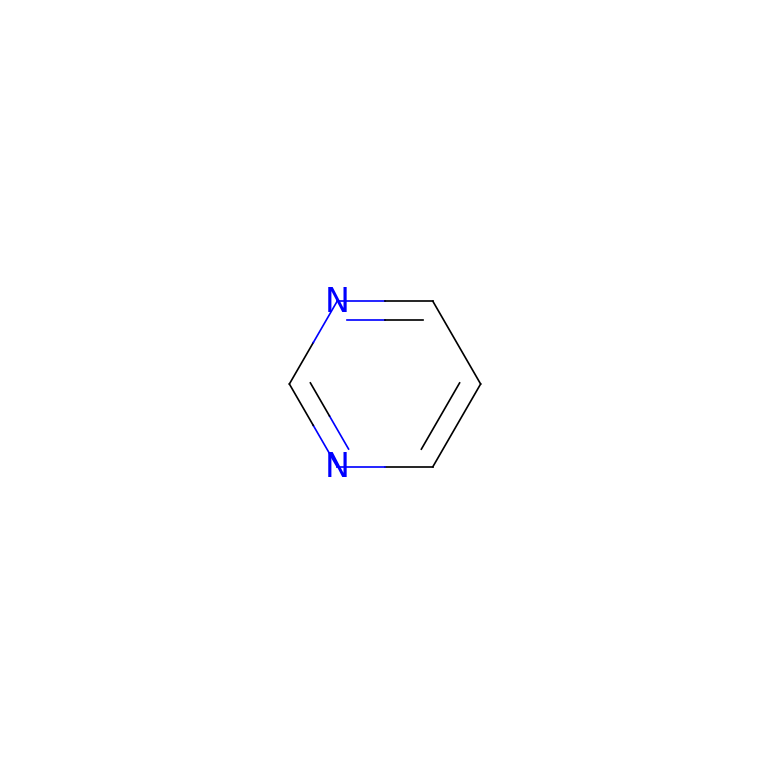 | C1=CN=CN=C1 | 52449 | 995 | 0.25 |
| 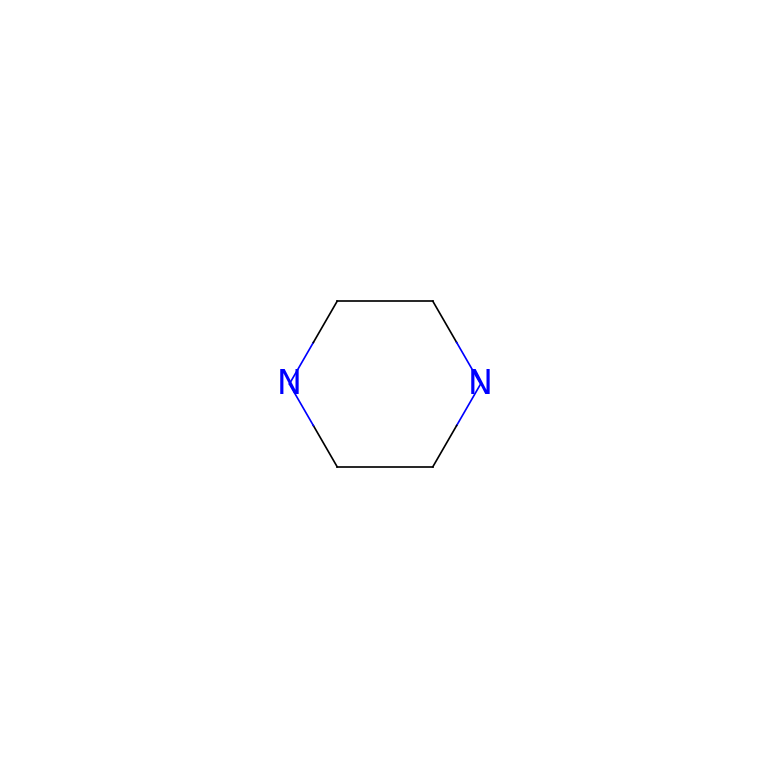 | N1[C][C]N[C][C]1 | 3799 | 551 | 0.45 |
| 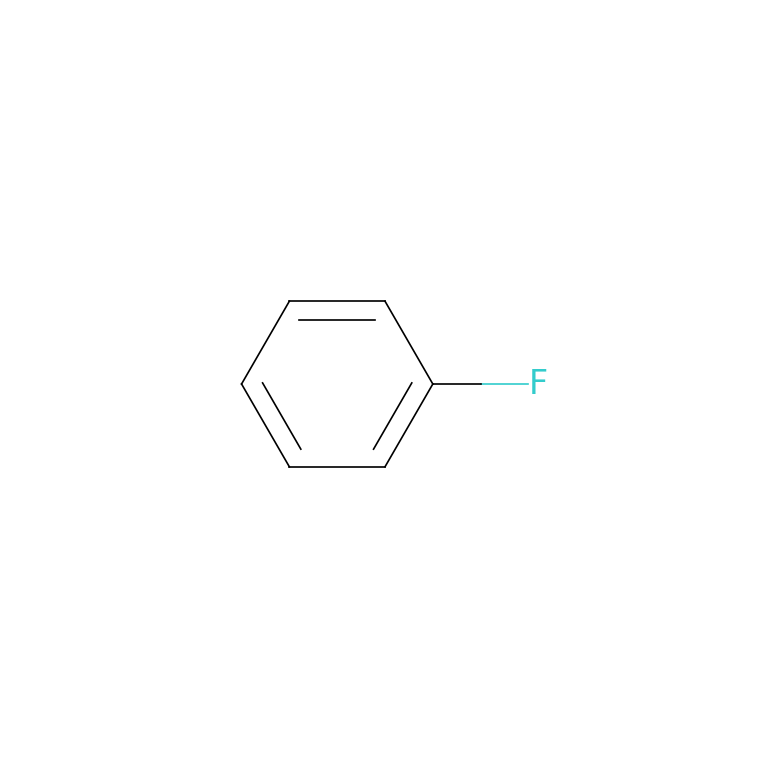 | FC1=CC=CC=C1 | 4243 | 523 | 0.45 |
| 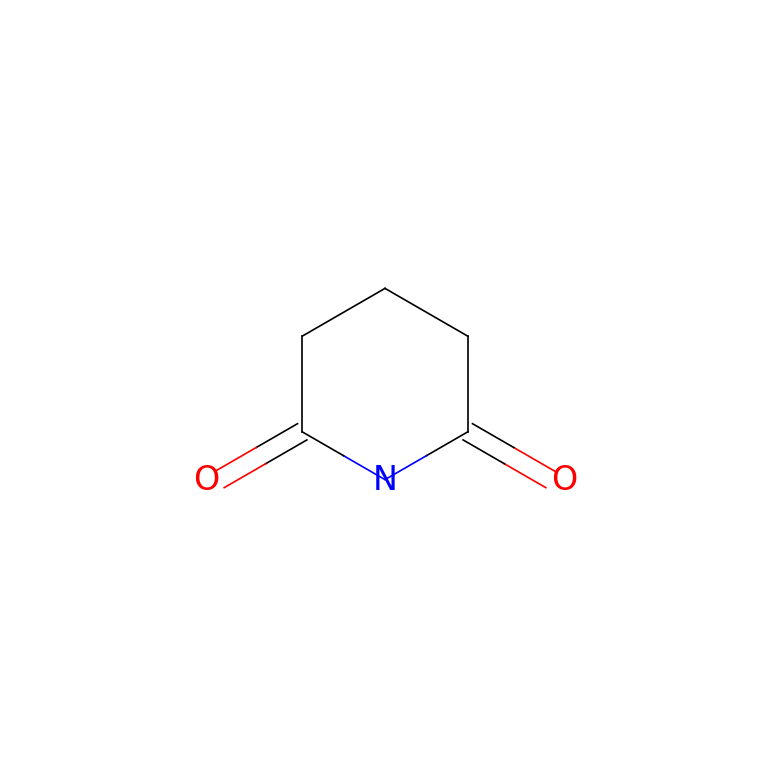 | O=C1NC(=O)CC[C]1 | 170 | 26 | 0.65 |
| 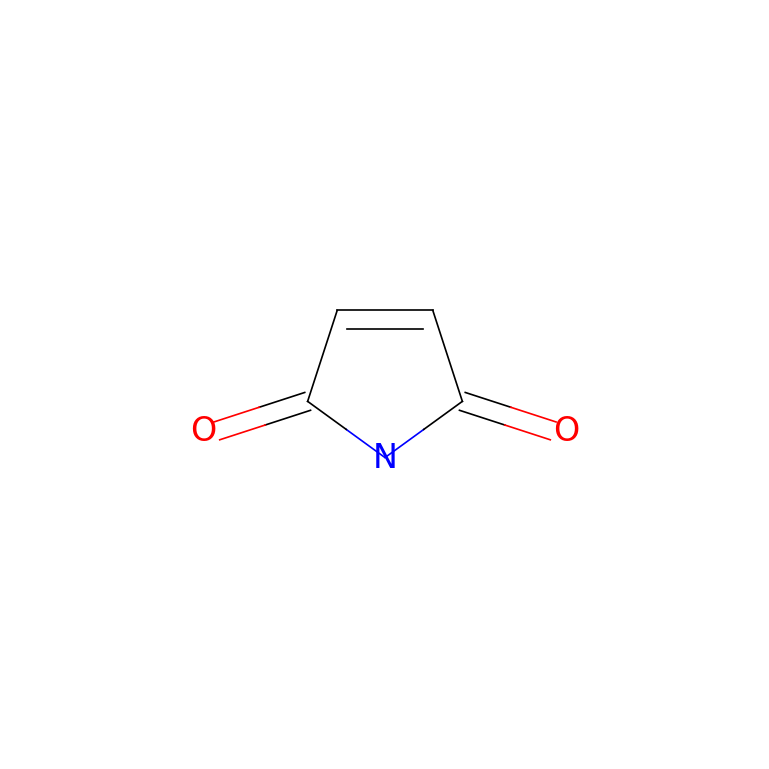 | C1(C=CC(N1)=O)=O | 485 | 82 | 0.60 |
| 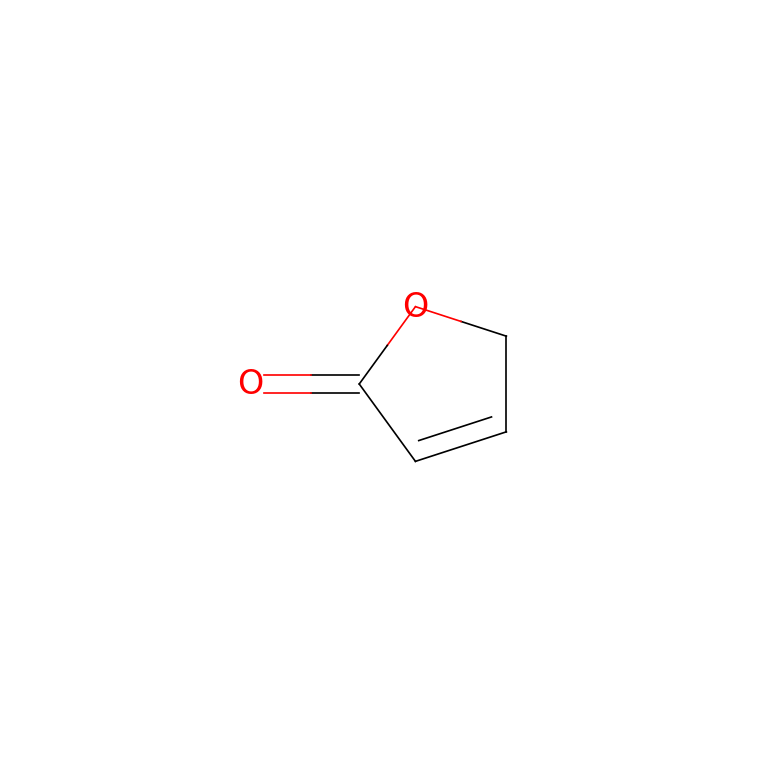 | C1=CCOC1=O | 357 | 96 | 0.65 |
