# Supplementary material for: New computational protein design methods for de novo small molecule binding sites
Source: PLoS Comput Biol. 2020 Oct 5;16(10):e1008178. doi: 10.1371/journal.pcbi.1008178 (PMC7575090; doi:10.1371/journal.pcbi.1008178)
Supplement: S2 Table — Full name and chemical component identifier for ligands used for the Match comparison benchmark and forward design with matched composited binding sites. All existing PDBs where a protein creates a contact interface with the ligand are provided. (DOCX) [file pcbi.1008178.s008.docx]

**S2 Table: Application Ligands**

| **Ligand** | **Chemical Component Identifier** | **Complex PDB(s)** |
| --- | --- | --- |
| (5Z)-5-(3,5-difluoro-4-hydroxybenzylidene)-2,3-dimethyl-3,5-dihydro-4H-imidazol-4-one | 38E | 6CZH, 6CZI |
| 8,9-DIHYDRO-9-HYDROXY-AFLATOXIN B1 | AFN | None. |
| ATRAZINE | ATZ | 5PRC |
| DIGOXIGENIN | DOG | 1LKE, 3RA7, 4J8T, 5BVB |
| IBUPROFEN | IBP | 6U4X, 5JQB, 4RS0,  4PH9, 4JTR, 3P6H, 3IB2, 2WD9, 2PWS, 2BXG, 1EQG |
| (E)-imidacloprid | IM4 | 3WTH, 3C79 |
| LUMIFLAVIN | LFN | 6ASL, 2CCC |
| Naproxen | NPS | 4ZBR, 4OR0, 4PO0, 4JQ1, 4FJP, 3R58, 3NT1, 2VDB |
