# Supplementary material for: New computational protein design methods for de novo small molecule binding sites
Source: PLoS Comput Biol. 2020 Oct 5;16(10):e1008178. doi: 10.1371/journal.pcbi.1008178 (PMC7575090; doi:10.1371/journal.pcbi.1008178)
Supplement: S3 Table — PDB ID, PDB description, ligand chemical component identifier, and full ligand name for BindingMOAD protein-ligand complexes applied in the binding site recovery benchmark. (DOCX) [file pcbi.1008178.s009.docx]

**S3 Table: BindingMOAD Complexes used for Binding Site Sequence Recovery**

| **PDB** | **PDB Description** | **Ligand** | **Ligand Name** |
| --- | --- | --- | --- |
| 6M9B | Wild-type streptavidin in complex with biotin solved by native SAD with data collected at 6 keV | BTN | Biotin |
| 5T52 | LECTIN FROM BAUHINIA FORFICATA IN COMPLEX WITH GALNAC | NGA | N-ACETYL-D-GALACTOSAMINE |
| 1LNM | ANTICALIN DIGA16 IN COMPLEX WITH DIGITOXIGENIN | DTX | DIGITOXIGENIN |
| 5HZ6 | FABP4 in complex with 6-Chloro-2-isopropyl-4-(3-isopropyl-phenyl)-quinoline-3-carboxylic acid | 65Y | 6-Chloro-2-isopropyl-4-(3-isopropyl-phenyl)-quinoline-3-carboxylic acid |
| 5HZ8 | FABP4_3 in complex with 6,8-Dichloro-4-phenyl-2-piperidin-1-yl-quinoline-3-carboxylic acid | 65Z | 6,8-dichloro-4-phenyl-2-(piperidin-1-yl)quinoline-3-carboxylic acid |
| 1LKE | ENGINEERED LIPOCALIN DIGA16 IN COMPLEX WITH DIGOXIGENIN | DOG | DIGOXIGENIN |
| 1N0S | ENGINEERED LIPOCALIN FLUA IN COMPLEX WITH FLUORESCEIN | FLU | 2-(6-HYDROXY-3-OXO-3H-XANTHEN-9-YL)-BENZOIC ACID |
| 3OKI | Crystal structure of human FXR in complex with (2S)-2-[2-(4-chlorophenyl)-1H-benzimidazol-1-yl]-N,2-dicyclohexylethanamide | OKI | (2S)-2-[2-(4-chlorophenyl)-1H-benzimidazol-1-yl]-N,2-dicyclohexylethanamide |
| 5EDB | human fatty acid binding protein 4 in complex with 6-Chloro-2-methyl-4-phenyl-quinoline-3-carboxylic acid at 1.18A | 5M8 | 6-chloranyl-2-methyl-4-phenyl-quinoline-3-carboxylic acid |
| 5URA | Enantiomer-Specific Binding of the Potent Antinociceptive Agent SBFI-26 to Anandamide transporters FABP7 | 8KS | (1S,2S,3S,4S)-3-{[(naphthalen-1-yl)oxy]carbonyl}-2,4-diphenylcyclobutane-1-carboxylic acid |
| 1SRI | STRUCTURE-BASED DESIGN OF SYNTHETIC AZOBENZENE LIGANDS FOR STREPTAVIDIN | DMB | 2-((3',5'-DIMETHYL-4'-HYDROXYPHENYL)AZO)BENZOIC ACID |
| 1TOU | Crystal structure of human adipocyte fatty acid binding protein in complex with a non-covalent ligand | B1V | 2-[(2-OXO-2-PIPERIDIN-1-YLETHYL)SULFANYL]-6-(TRIFLUOROMETHYL)PYRIMIDIN-4-OL |
| 2IZL | STREPTAVIDIN-2-IMINOBIOTIN PH 7.3 I222 COMPLEX | IMI | 2-IMINOBIOTIN |
| 4AFH | Capitella teleta AChBP in complex with lobeline | L0B | Alpha-Lobeline |
| 4QAC | X-RAY STRUCTURE OF ACETYLCHOLINE BINDING PROTEIN (ACHBP) IN COMPLEX WITH 4-(4-methylpiperidin-1-yl)-6-(4-(trifluoromethyl)phenyl)pyrimidin-2-amine | KK3 | 4-(4-methylpiperidin-1-yl)-6-[4-(trifluoromethyl)phenyl]pyrimidin-2-amine |
| 2QRY | Periplasmic thiamin binding protein | TPS | THIAMIN PHOSPHATE |
| 4AFG | Capitella teleta AChBP in complex with varenicline | QMR | VARENICLINE |
| 4B5D | Capitella teleta AChBP in complex with psychonicline (3-((2(S)- Azetidinyl)methoxy)-5-((1S,2R)-2-(2-hydroxyethyl)cyclopropyl)pyridine) | SW4 | 2-[(1R,2S)-2-[5-[[(2S)-azetidin-2-yl]methoxy]pyridin-3-yl]cyclopropyl]ethanol |
| 5J5G | X-Ray Crystal Structure of Acetylcholine Binding Protein (AChBP) in Complex with 6-(4-methoxyphenyl)-N4,N4-bis[(pyridin-2-yl)methyl]pyrimidine-2,4-diamine | 6GF | 6-(4-methoxyphenyl)-N~4~,N~4~-bis[(pyridin-2-yl)methyl]pyrimidine-2,4-diamine |
| 3CZ1 | Dimeric crystal structure of a pheromone binding protein from Apis mellifera in complex with the n-butyl benzene sulfonamide at pH 7.0 | NBB | N-BUTYL-BENZENESULFONAMIDE |
| 2XN3 | Crystal structure of thyroxine-binding globulin complexed with mefenamic acid | ID8 | 2-[(2,3-DIMETHYLPHENYL)AMINO]BENZOIC ACID |
| 5J5I | X-Ray Crystal Structure of Acetylcholine Binding Protein (AChBP) in Complex with 4-(2-amino-6-{bis[(pyridin-2-yl)methyl]amino}pyrimidin-4-yl)phenol | 6GM | 4-(2-amino-6-{bis[(pyridin-2-yl)methyl]amino}pyrimidin-4-yl)phenol |
