# Supplementary material for: New computational protein design methods for de novo small molecule binding sites
Source: PLoS Comput Biol. 2020 Oct 5;16(10):e1008178. doi: 10.1371/journal.pcbi.1008178 (PMC7575090; doi:10.1371/journal.pcbi.1008178)
Supplement: S4 Table — Statistics for complementary rotamers generated and applied to the binding site recovery benchmark. Rosetta numbering and the corresponding PDB numbering for scaffold PDBs (as in S3 File) are provided. Here we report the number of designable positions as well as the number of complementary rotamers generated, accepted (i.e. passed Rosetta energy and RMSD filters, see Methods), and applied to design for each benchmark binding site. Only the best 50 rotamers per residue type per position were applied top design. (DOCX) [file pcbi.1008178.s010.docx]

**S4 Table. Binding Site Benchmark Design Details**

| **Benchmark Complex** | **Designable_positions (Rosetta numbering)** | **Designable_positions (PDB numbering)** | **Total_rotamers,**  **accepted** | **Total_rotamers,**  **applied** | **Total_rotamers,**  **generated** | **Designable_positions, count** |
| --- | --- | --- | --- | --- | --- | --- |
| 5edb-clean | 13,16,19,20,22,23,24,26,28,30,33,36,39,56,58,60,61,63,77,78,79,80,107,127,129,131 | 11A ,14A ,17A ,20A ,21A ,24A ,26A ,34A ,37A ,41A ,43A ,52A ,54A ,56A ,58A ,59A ,61A ,75A ,76A ,77A ,105A ,116A ,125A ,127A ,129A | 7972 | 1544 | 123299 | 26 |
| 5HZ6-clean | 10,13,16,19,20,23,25,29,30,32,33,36,40,41,51,53,54,55,57,58,59,60,74,75,76,77,124,126,128 | 11A ,14A ,17A ,20A ,21A ,26A ,30A ,31A ,33A ,34A ,37A ,41A ,43A ,52A ,54A ,55A ,56A ,58A ,59A ,60A ,61A ,75A ,76A ,77A ,114A ,116A ,125A ,127A ,129A | 9768 | 2024 | 166273 | 29 |
| 5HZ8-clean | 14,17,20,23,24,29,34,37,40,44,57,59,61,62,64,78,79,80,81,108,110,119,121,130,132 | 11A ,14A ,17A ,20A ,21A ,26A ,31A ,34A ,37A ,38A ,41A ,54A ,56A ,58A ,59A ,61A ,75A ,76A ,77A ,105A ,107A ,114A ,116A ,118A ,127A ,129A | 10876 | 1901 | 110344 | 25 |
| 5J5G-clean | 151,152,193,200,253,274,276,277,278,279,296,324,325,332,333,334,335,385 | 143A ,144A ,146A ,148A ,185A ,190A ,192A ,32B ,34B ,36B ,53B ,55B ,56B ,57B ,58B ,73B ,81B ,101B ,103B ,104B ,106B ,111B ,112B ,113B ,114B ,164B | 3798 | 471 | 47205 | 18 |
| 5J5I-clean | 42,61,63,65,66,111,112,119,120,121,122,172,364,365,367,404,405,406,411,413 | -6A ,32A ,34A ,36A ,53A ,55A ,57A ,58A ,73A ,78A ,81A ,99A ,101A ,103A ,104A ,106A ,111A ,112A ,113A ,114A ,164A ,143E ,144E ,146E ,183E ,184E ,185E ,190E ,192E | 2720 | 604 | 62164 | 20 |
| 5ura-clean | 13,16,19,20,22,23,24,28,32,33,35,37,38,39,56,58,60,61,63,77,78,79,80,118,120,129,131 | 11A ,14A ,17A ,20A ,21A ,26A ,33A ,35A ,37A ,41A ,54A ,56A ,58A ,59A ,61A ,75A ,76A ,77A ,105A ,116A ,118A ,127A ,129A | 6594 | 1636 | 130454 | 27 |
| 1TOU-clean | 10,13,16,29,33,36,37,39,52,53,54,55,57,58,59,60,61,74,75,76,77,93,104,115,126 | 9A ,10A ,13A ,16A ,19A ,20A ,25A ,32A ,33A ,36A ,37A ,39A ,40A ,41A ,42A ,51A ,53A ,54A ,55A ,57A ,58A ,59A ,60A ,74A ,75A ,76A ,113A ,115A ,124A ,126A ,128A | 413 | 177 | 51463 | 25 |
| 6m9b-clean | 9,10,11,13,15,29,30,31,32,33,35,36,40,63,65,72,74,75,76,92,94,96,110,114,116,222,224,225 | 15A ,16A ,17A ,19A ,21A ,35A ,36A ,37A ,38A ,39A ,41A ,42A ,45A ,46A ,48A ,67A ,69A ,71A ,73A ,78A ,80A ,82A ,100A ,102A ,116A ,120A ,122A ,110D ,112D ,113D | 12346 | 2964 | 148958 | 28 |
| 1sri-clean | 11,12,13,15,17,31,33,34,35,37,38,42,62,64,71,73,74,75,77,91,93,95,109,113,115 | 23A ,24A ,25A ,27A ,29A ,43A ,45A ,46A ,47A ,49A ,50A ,54A ,56A ,75A ,77A ,79A ,86A ,88A ,89A ,90A ,92A ,106A ,108A ,110A ,124A ,128A ,130A ,118B ,120B ,121B | 6827 | 2017 | 124187 | 25 |
| 1lke-clean | 24,27,30,31,34,35,41,43,52,54,65,82,84,89,91,95,110,116,118,120,122 | 28A ,31A ,35A ,38A ,39A ,45A ,47A ,56A ,58A ,69A ,84A ,86A ,88A ,93A ,95A ,99A ,114A ,127A ,129A ,131A | 15647 | 3260 | 135535 | 21 |
| 1lnm-clean | 24,27,30,31,34,35,41,43,52,54,65,80,81,82,83,84,86,91,92,95,110,119,121,123 | 28A ,31A ,35A ,38A ,39A ,45A ,47A ,56A ,58A ,60A ,69A ,73A ,84A ,86A ,88A ,90A ,93A ,95A ,99A ,114A ,127A ,129A ,131A | 9306 | 2132 | 142313 | 24 |
| 1n0s-clean | 28,31,45,56,58,60,69,73,84,86,88,95,97,99,100,113,114,126,127,129,131,292 | 11A ,19A ,28A ,31A ,45A ,47A ,56A ,58A ,60A ,69A ,73A ,84A ,86A ,88A ,90A ,95A ,97A ,99A ,100A ,102A ,111A ,113A ,114A ,127A ,128A ,129A ,131A ,154A | 11272 | 2171 | 126015 | 22 |
| 2XN3-clean | 2,5,9,218,220,222,228,230,248,251,252,254,255,256,258 | 20A ,23A ,24A ,27A ,225A ,227A ,229A ,236A ,238A ,240A ,246A ,248A ,250A ,266A ,268A ,269A ,270A ,272A ,273A ,276A | 1145 | 358 | 105834 | 15 |
| 2IZL-clean | 11,12,13,15,31,32,33,34,35,37,38,42,65,67,74,76,78,80,96,98,112,116,230,231 | 23B ,24B ,25B ,27B ,29B ,43B ,45B ,46B ,47B ,49B ,50B ,54B ,56B ,75B ,77B ,79B ,86B ,88B ,90B ,92B ,106B ,108B ,110B ,124B ,128B ,130B ,118D ,120D ,121D ,124D | 7597 | 1843 | 97588 | 24 |
| 4QAC-clean | 95,97,150,151,152,153,154,187,189,196,198,247,264,310,325,327 | 19A ,85A ,87A ,89A ,139A ,142A ,143A ,144A ,145A ,146A ,183A ,185A ,190A ,192A ,194A ,36B ,53B ,55B ,73B ,99B ,102B ,104B ,106B ,112B ,114B ,116B ,164B | 4164 | 911 | 59939 | 16 |
| 4AFH-clean | 98,100,102,152,153,154,155,156,192,194,201,277,325,328,341,388 | 25A ,98A ,100A ,102A ,108A ,152A ,153A ,154A ,155A ,156A ,192A ,194A ,201A ,43B ,64B ,66B ,88B ,112B ,115B ,128B ,175B | 3767 | 1227 | 92145 | 16 |
| 3CZ1-clean | 32,33,37,43,46,47,48,50,51,54,56,57,99,100,102,103,106,107,114,115,116,117,122,125 | 13A ,17A ,34A ,35A ,39A ,48A ,49A ,50A ,52A ,53A ,58A ,59A ,70A ,73A ,74A ,92A ,101A ,102A ,104A ,105A ,108A ,109A ,115A ,116A ,117A ,118A ,119A ,9B ,12B ,16B | 2949 | 1257 | 111069 | 24 |
| 5t52-clean | 43,77,78,93,94,97,98,106,122,124,125,126,127,129 | 42A ,43A ,77A ,78A ,93A ,94A ,97A ,98A ,102A ,122A ,124A ,125A ,126A ,127A ,209A ,210A ,212A ,213A | 5968 | 1013 | 63733 | 14 |
| 3oki-clean | 21,22,27,28,31,35,36,41,44,45,48,49,52,86,87,89,90,91,92,93,94,106,109,110,113,115,120,123,126,127,201,208,212 | 264A ,267A ,268A ,273A ,274A ,277A ,281A ,287A ,290A ,291A ,293A ,294A ,295A ,298A ,299A ,302A ,332A ,333A ,335A ,336A ,337A ,338A ,339A ,340A ,344A ,352A ,355A ,356A ,359A ,361A ,366A ,369A ,372A ,373A ,390A ,447A ,454A ,458A ,473A | 5090 | 1689 | 100259 | 33 |
| 4AFG-clean | 99,151,152,153,154,155,191,192,193,197,198,199,200,275,277,278,326,329,337,338,339,386 | 25A ,100A ,152A ,153A ,154A ,155A ,156A ,194A ,198A ,199A ,201A ,41B ,64B ,66B ,86B ,88B ,115B ,116B ,118B ,120B ,126B ,128B ,165B ,175B | 1746 | 717 | 63564 | 22 |
| 4B5D-clean | 101,152,153,154,155,193,200,276,296,298,326,327,328,330,336,337,338 | 98A ,100A ,102A ,152A ,153A ,154A ,155A ,156A ,194A ,198A ,199A ,201A ,203A ,41B ,64B ,66B ,68B ,86B ,88B ,115B ,116B ,117B ,118B ,120B ,126B ,127B ,128B ,165B ,174B ,175B | 3738 | 761 | 62034 | 17 |
| 2QRY-clean | 9,10,11,65,103,107,138,144,179,180,183,196,197,199,200,203,204,225,227,261,262,263 | 27A ,29A ,30A ,59A ,83A ,84A ,120A ,121A ,125A ,128A ,156A ,161A ,162A ,197A ,198A ,201A ,205A ,213A ,214A ,215A ,217A ,218A ,221A ,222A ,243A ,245A ,275A ,279A ,280A ,281A | 827 | 395 | 58642 | 22 |
