# Supplementary material for: New computational protein design methods for de novo small molecule binding sites
Source: PLoS Comput Biol. 2020 Oct 5;16(10):e1008178. doi: 10.1371/journal.pcbi.1008178 (PMC7575090; doi:10.1371/journal.pcbi.1008178)
Supplement: S5 Table — Statistics for complementary rotamers generated and applied to forward design of complexes generated with RosettaMatch for application ligands. Match scaffold PDB ID, scaffold description, complex designable positions in Rosetta numbering, ligand chemical component identifier, and constraint file that yielded the match (as in S4 File) are provided. We also report the number of designable positions as well as the number of complementary rotamers generated, accepted (i.e. passed Rosetta energy and RMSD filters, see Methods), and applied to design for each benchmark binding site. Only the best 50 rotamers per residue type per position were applied top design. (DOCX) [file pcbi.1008178.s011.docx]

**S5 Table. Forward Design Complexes Design Details**

| Design | Scaffold (PDB ID:Chain) | Scaffold Description | Ligand | Binding Site Definition | Designable_positions (Rosetta numbering) | Total_rotamers, accepted | Total_rotamers, applied | Total_rotamers, generated | Designable_positions, count |
| --- | --- | --- | --- | --- | --- | --- | --- | --- | --- |
| ATZ_1TP6 | 1TP6:A | 1.5 A Crystal Structure of a NTF-2 Like Protein of Unknown Function PA1314 from Pseudomonas aeruginosa | ATZ | ATZ_0022-iter_0-fuzz_0-1_228_1014_2314 | 7,8,10,12,15,16,17,30,31,34,40,42,48,53,57,64,67,69,71,88,90,92,99,101,103,105,117,120,122 | 1414 | 437 | 73121 | 29 |
| ATZ_1Q40 | 1Q40:A | Crystal structure of the C. albicans Mtr2-Mex67 M domain complex | ATZ | ATZ_0018-iter_0-fuzz_0-1_655_1670_2136 | 14,15,42,48,49,50,51,67,70,77,79,103,105,133,135,136,137,138,155,156,157,158,159 | 2715 | 727 | 103619 | 23 |
| DOG_3ER7 | 3ER7:B | Crystal structure of NTF2-like protein of unknown function (YP_001812677.1) from EXIGUOBACTERIUM SP. 255-15 at 1.50 A resolution | DOG | DOG_0001-iter_0-fuzz_0-1_288_1136_2530 | 6,7,10,11,14,23,35,54,57,61,62,63,64,67,78,79,80,81,82,92,94,95,98,111,113,116 | 10809 | 2592 | 165516 | 26 |
| IBP_1JVX | 1JVX:A | Maltodextrin-binding protein variant D207C/A301GS/P316C cross-linked in crystal | IBP | IBP_0007-iter_0-fuzz_0-1_759_1061_2710 | 11,14,15,42,43,44,45,61,62,63,65,66,111,113,153,156,210,230,258,262,331,338,341,345 | 85 | 85 | 84936 | 24 |
| IBP_1TUH | 1TUH:A | Structure of Bal32a from a Soil-Derived Mobile Gene Cassette | IBP | IBP_0019-iter_0-fuzz_0-1_895_2639_2948 | 14,17,25,35,36,37,41,43,44,47,53,56,57,59,63,68,70,87,98,100,106,117,118,119,120,126,127,130 | 1025 | 418 | 98950 | 28 |
| IBP_3ECF | 3ECF:A | Crystal structure of an ntf2-like protein (ava_4193) from anabaena variabilis atcc 29413 at 1.90 A resolution | IBP | IBP_0004-iter_0-fuzz_0-1_823_1135_2552 | 8,9,11,13,15,16,21,23,24,26,30,41,44,47,48,50,52,55,61,77,79,91,93,100,103 | 3753 | 1248 | 120051 | 25 |
| IM4_1JKG | 1JKG:A | Structural basis for the recognition of a nucleoporin FG-repeat by the NTF2-like domain of TAP-p15 mRNA nuclear export factor | IM4 | IM4_0007-iter_0-fuzz_0-1_794_1164_1278 | 23,24,26,28,31,34,44,46,47,51,57,60,61,62,63,64,66,67,69,71,94,100,108,110,130,134 | 3444 | 767 | 100199 | 26 |
| IM4_1Z1S | 1Z1S:A | Crystal Structure of Putative Isomerase PA3332 from Pseudomonas aeruginosa | IM4 | IM4_0017-iter_0-fuzz_0-1_742_1081_1151 | 17,26,29,39,58,62,65,68,69,70,71,97,104,106,108,122,126,130,131,134 | 412 | 117 | 51352 | 20 |
| IM4_3EMM | 3EMM:A | X-ray structure of protein from Arabidopsis thaliana AT1G79260 with Bound Heme | IM4 | IM4_0018-iter_0-fuzz_0-1_1192_1270_1360 | 25,35,51,53,63,81,83,85,87,89,91,104,106,109,112,115,118,119,120,121,131,132,134,135,137,142,145,148 | 2302 | 737 | 88738 | 28 |
| IM4_3GZB | 3GZB:A | Crystal structure of putative SnoaL-like polyketide cyclase (YP_001182657.1) from Shewanella putrefaciens CN-32 at 1.44 A resolution | IM4 | IM4_0002-iter_0-fuzz_0-1_1083_1296_1408 | 29,30,32,34,35,36,38,41,44,53,66,67,69,70,73,74,77,79,80,82,100,102,124,126,135,140,142 | 816 | 213 | 76121 | 27 |
| LFN_2FNC | 2FNC:A | Thermotoga maritima maltotriose binding protein bound with maltotriose. | LFN | LFN_0001-iter_0-fuzz_0-1_2065_2410_2917 | 7,8,9,10,34,35,39,57,59,106,148,151,154,208,225,227,229,261,296 | 2192 | 598 | 40354 | 19 |
| LFN_2OWP | 2OWP:A | Crystal structure of a cystatin-like fold protein (bxe_b1374) from burkholderia xenovorans lb400 at 2.00 A resolution | LFN | LFN_0001-iter_0-fuzz_0-1_1291_1909_2515 | 17,20,21,23,25,32,44,47,61,67,73,76,90,92,94,103,105,107,118,121,125 | 2086 | 547 | 49014 | 21 |
| NPS_2RCD | 2RCD:A | CRYSTAL STRUCTURE OF A PROTEIN WITH UNKNOWN FUNCTION FROM DUF3225 FAMILY (ECA3500) FROM PECTOBACTERIUM ATROSEPTICUM SCRI1043 AT 2.32 A RESOLUTION | NPS | NPS_0005-iter_0-fuzz_0-1_140_1081_2929 | 20,21,23,24,26,28,30,32,35,38,39,47,50,52,54,60,61,63,64,65,67,69,72,74,95,106,108,110,119,122 | 6517 | 1869 | 130983 | 30 |
| NPS_3GZR | 3GZR:A | CRYSTAL STRUCTURE OF AN UNCHARACTERIZED PROTEIN WITH A CYSTATIN-LIKE FOLD (CC_2572) FROM CAULOBACTER VIBRIOIDES AT 1.40 A RESOLUTION | NPS | NPS_0005-iter_0-fuzz_0-1_47_259_1081 | 14,15,18,21,26,36,39,40,42,44,50,53,54,56,57,58,60,61,67,88,90,95,101,106,108,126,128 | 3974 | 1142 | 106224 | 27 |
