# Supplementary material for: New computational protein design methods for de novo small molecule binding sites
Source: PLoS Comput Biol. 2020 Oct 5;16(10):e1008178. doi: 10.1371/journal.pcbi.1008178 (PMC7575090; doi:10.1371/journal.pcbi.1008178)
Supplement: S6 Table — Counts out of 14 composite binding site matches where median metrics across 5000 designs that utilized composite RotamerSets with special_rot bonus values were improved as compared to designs generated with unmodified Packer. PackStat and shape complementarity are considered improved if the median increased relative to the unmodified Packer. Ligand SASA, binding strain (bindingstrain), residue interaction energy (residueie), and RosettaHoles are considered improved if the median decreased relative to the unmodified packer. Number of hydrogen bonds made with the ligand (hbonds) is considered improved if the median count is more than the median count for the unmodified Packer. Heavy buried unsatisfied hydrogen bond donor/acceptor count (heavyburiedunsats) is considered improved if the median count is less than the median count for the unmodified Packer. (DOCX) [file pcbi.1008178.s012.docx]

**S6 Table. Counts for Improved Metrics Across All Attempted Designs.**

| **special_rot bonus** | **bindingstrain** | **hbonds** | **heavyburiedunsats** | **holes** | **packstat** | **residueie** | **ligand_sasa** | **shapecomplementarity** |
| --- | --- | --- | --- | --- | --- | --- | --- | --- |
| 0 | 7 | 1 | 2 | 9 | 11 | 8 | 6 | 10 |
| -1.5 | 6 | 1 | 1 | 8 | 7 | 7 | 7 | 11 |
| -3.0 | 2 | 3 | 0 | 9 | 8 | 8 | 12 | 11 |
| -4.0 | 2 | 3 | 2 | 8 | 8 | 5 | 12 | 9 |
