# Supplementary material for: New computational protein design methods for de novo small molecule binding sites
Source: PLoS Comput Biol. 2020 Oct 5;16(10):e1008178. doi: 10.1371/journal.pcbi.1008178 (PMC7575090; doi:10.1371/journal.pcbi.1008178)
Supplement: S7 Table — Descriptions of individual feature vector components used to cluster residue-fragment interactions into unique contact modes using hierarchical agglomerative clustering. (DOCX) [file pcbi.1008178.s013.docx]

**S7 Table: Feature Vector Components**

| **Value** | **Description** |
| --- | --- |
| Angstroms | X component, vector from fragment centroid to closest residue atom |
| Angstroms | Y component, vector from fragment centroid to closest residue atom |
| Angstroms | Z component, vector from fragment centroid to closest residue atom |
| 0 \| 1 | Sidechain contact (1) OR backbone contact (0) |
| 0 \| 1 | Ligand Polar Contact (1) OR Ligand Non-polar Contact (0) |
| 0 \| 1 | Side chain possesses (1) or does not possess (0) hydrogen bond donor/acceptor (DEHKNQRSTY) |
| 0 \| 1 | Hydrophobic, aliphatic (AILV) contact (1) or not (0) |
| 0 \| 1 | Hydrophobic, aromatic (FWY) contact (1) or not (0) |
| 0 \| 1 | Polar (NCQMST) contact (1) or not (0) |
| 0 \| 1 | Charged, Acidic (DE) contact (1) or not (0) |
| 0 \| 1 | Charged, Basic (HKR) contact (1) or not (0) |
| 0 \| 1 | Glycine contact (1) or not (0) |
| 0 \| 1 | Proline contact (1) or not (0) |
| 0 \| 1 | Backbone carbonyl contact (1) or not (0) |
| 0 \| 1 | Backbone amino contact (1) or not (0) |
| 0 \| 1 | Backbone C/CA contact (1) or not (0) |
