# Supplementary figures and images for: New computational protein design methods for de novo small molecule binding sites
Source: PLoS Comput Biol. 2020 Oct 5;16(10):e1008178. doi: 10.1371/journal.pcbi.1008178 (PMC7575090; doi:10.1371/journal.pcbi.1008178)

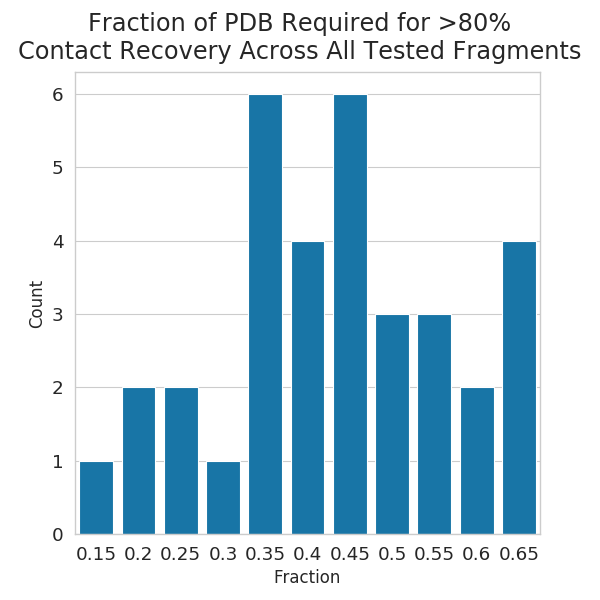

Supplement: S1 Fig — Counts for the fraction of the PDB required to achieve >80% contact recovery for all 34 tested fragments (as in S1 Table). (PNG) [file pcbi.1008178.s014.png]

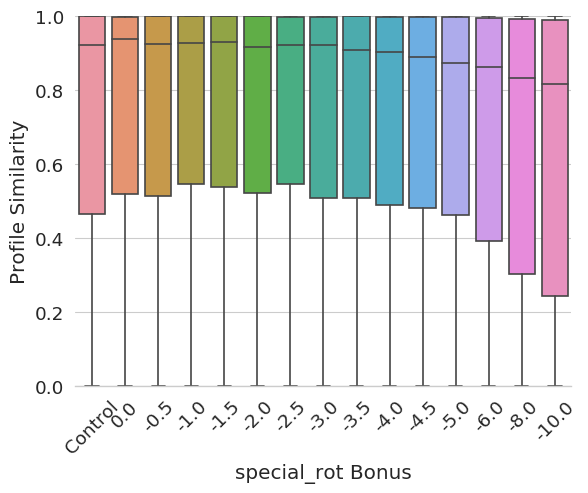

Supplement: S2 Fig — Profile similarities as depicted in Fig 4B, except profile similarities for all designable positions are included. (PNG) [file pcbi.1008178.s015.png]
